# Plasmonic MoO_3−_ *_x_*/Ag Photocatalyst for the Fixation of N_2_ from Air with the Solar Energy Conversion Efficiency Reaching over 0.28%

**DOI:** 10.1002/adma.202509652

**Published:** 2025-08-11

**Authors:** Jingtian Hu, Ke An, Yifei Ren, Feng Ryan Wang, Yanzhen Guo, Xiaopeng Bai, Degao Wang, Jianfang Wang

**Affiliations:** ^1^ Zhejiang Key Laboratory of Data‐Driven High‐Safety Energy Materials and Applications Advanced Interdisciplinary Sciences Research (AiR) Center Ningbo Institute of Materials Technology and Engineering Chinese Academy of Sciences Ningbo Zhejiang 315201 China; ^2^ Department of Physics The Chinese University of Hong Kong Shatin Hong Kong SAR 999077 China; ^3^ Department of Chemical Engineering University College London London WC1E 7JE UK; ^4^ Henan Provincial Key Laboratory of Nanocomposites and Applications Institute of Nanostructured Functional Materials Huanghe Science and Technology College Zhengzhou Henan 450006 China

**Keywords:** molybdenum oxide, nitrogen photofixation, non‐noble‐metal plasmonic nanoparticles, plasmonic photocatalysis, Schottky‐barrier‐free photocatalysts

## Abstract

Storing solar energy in chemical bonds through photocatalysis under ambient conditions is of great importance for sustainable development and carbon neutrality. In addition to the design of new photocatalysts with high activities, efficient solar energy delivery and the acceleration of reactant mass transfer kinetics are also crucial for efficient energy conversion. Herein, a new type of plasmonic Schottky‐barrier‐free MoO_3−_ *_x_*/Ag photocatalyst is designed for efficient NH_3_ production. The photocatalyst exhibits strong light absorption and utilization under sunlight illumination. The construction of a bilayer system reduces the light attenuation by water in the near‐infrared region and accelerates the N_2_ mass transfer kinetics. As a result, the photocatalytic activity is largely boosted. A high solar‐to‐chemical energy conversion efficiency of over 0.28% (±0.01%) is reached with air directly used as the feeding gas. The study offers a promising pathway for the rational design of photocatalysts and photocatalytic platforms, enabling greatly enhanced solar‐to‐chemical energy conversion.

## Graphical abstract

A plasmonic MoO_3−_ *_x_*/Ag photocatalyst is designed for efficient N_2_ photofixation. It exhibits superior light absorption, a Schottky‐barrier‐free feature, and plasmonic photothermal conversion. A diphase reaction platform is designed by loading the photocatalyst in a polymer film to suppress the light absorption of water and accelerate the mass transfer kinetics, giving a high photocatalytic efficiency of >0.28% from air.

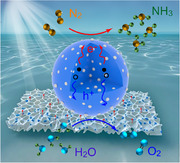

## 1 Introduction

Nitrogen fixation is essential to life as only fixed N_2_ can be utilized for the biological synthesis of all nitrogen‐containing organic compounds.^[^ ^1^, ^2^ ^]^ However, anthropogenic N_2_ fixation through the Haber–Bosch process consumes a large amount of energy and produces a large amount of CO_2_.^[^ ^3^, ^4^, ^5^, ^6^, ^7^ ^]^ Photocatalytic N_2_ fixation (PCNF), which directly converts air‐abundant N_2_ into valuable NH_3_ under mild conditions with solar energy as the sole driving force, has therefore attracted immense attention owing to its unparalleled ascendancy in theoretical zero‐energy input from the perspective of artificial photosynthesis and carbon neutrality.^[^ ^8^, ^9^, ^10^ ^]^ A photocatalytic process involves harvesting solar energy and converting it into usable chemical energy, which is characterized by the solar‐to‐chemical conversion efficiency (SCCE).^[^ ^11^ ^]^ The highest SCCE for PCNF has recently been reported to be 0.3%, which was calculated with H_2_O_2_ as the product and *∆G*_ammonia_ of 514.5 kJ mol^−1^ (see Supplementary Note 1, Supporting Information).^[^ ^12^ ^]^ Such SCCE values are insufficient for practical applications.^[^ ^11^, ^13^ ^]^

Although extensive efforts have been made to improve the efficiencies of various photocatalysts over past decades, such as surface engineering, morphology design, heterostructure construction, and elemental doping,^[^ ^14^, ^15^, ^16^, ^17^, ^18^ ^]^ the photocatalysts suffer from intrinsic imperfections, such as wide bandgaps of semiconductors,^[^ ^19^ ^]^ severe hot charge carrier recombination in plasmonic metals, commonly formed Schottky barriers at the junctions between plasmonic metals and semiconductors, and weak light absorption of plasmonic semiconductors in certain spectral regions.^[^ ^20^, ^21^, ^22^, ^23^ ^]^ A new type of semiconductor photocatalyst, named as Schottky‐barrier‐free plasmonic photocatalyst, such as MoO_3−_ *_x_*,^[^ ^12^, ^23^, ^24^ ^]^ Bi_2_O_3−_ *_x_*,^[^ ^25^ ^]^ and WO_3−_ *_x_*,^[^ ^26^ ^]^ has recently been reported. A large number of defects are introduced into the semiconductor, which greatly increases the concentration of free charge carriers so that a strong localized surface plasmon resonance (LSPR) is created. The introduced defects also serve as active sites for the chemisorption and activation of reactant molecules. The photocatalytic performance is greatly boosted without use of any hole scavenger. However, there exists a light absorption dip ≈400–500 nm, which limits the photocatalytic activity.^[^ ^27^, ^28^ ^]^ In addition, the drawbacks of photocatalytic devices and the sluggish reaction kinetics of N_2_ gas also limit the further improvement of SCCE in PCNF. For instance, the conventional slurry reactor, which has been the primary form in photocatalysis, suffers from the aggregation of photocatalyst powder, the severe light absorption in the near‐infrared (NIR) region by water, and the serious energy waste originating from continuous magnetic stirring.^[^ ^29^ ^]^ In addition, the poor saturated solubility (0.68 mM at 298 K and 1 atm) and limited diffusion coefficient (1.88 × 10**^−^** ^5^ cm^2^ s**^−^** ^1^ at 298 K) of N_2_ in water hamper the efficient mass transfer of N_2_ molecules, which in kinetics suppresses the reaction activity. Although recently proposed triphase photocatalytic platforms can greatly enhance the mass transfer kinetics of gas‐phase reactants through simple gas convection,^[^ ^30^, ^31^, ^32^ ^]^ such platforms still suffer from many constraints,^[^ ^29^, ^33^ ^],^ such as forced convection, complex instrument setups, and unsolved light absorption of water in the NIR region.

In this regard, we have designed a Schottky‐barrier‐free plasmonic MoO_3−_ *_x_*/Ag photocatalyst to not only fill up the intrinsic light absorption dip of the plasmonic MoO_3−_ *_x_* photocatalyst but also form an ohmic contact (Figure S1, Supporting Information) between MoO_3−_ *_x_* and Ag to ensure efficient hot charge carrier transportation and utilization. The photocatalyst exhibits strong light absorption from the ultraviolet to the NIR region, a greatly enhanced charge carrier density, and accelerated reaction kinetics. A floatable bilayer photocatalytic platform is further designed by employing polyfoam as the supporting material, hydrophilic porous poly(vinyl alcohol) (PVA) as the substrate, and plasmonic MoO_3−_ *_x_*/10 mol% Ag (Ag:(Ag + Mo) mol%, the loading amount of Ag nanoparticles: 57.98 mg *g*_cat_^−1^) as the photocatalyst for efficient solar energy harvesting and conversion. Located at the air–water interface, the floatable platform adsorbs an ultrathin water layer of ≈250 nm in thickness, ensuring superior light delivery with minimized water‐induced light attenuation. Moreover, the platform facilitates rapid reaction kinetics with boosted mass transfer of N_2_ molecules, breaking out the requirement for continuous stirring or forced convection. The immobilization of the MoO_3−_ *_x_*/10% Ag photocatalyst particles in the porous interlaced PVA network enables direct photofixation of N_2_ from air under one‐sun light illumination. With this system, a record‐high SCCE of 0.41% (±0.03%) is achieved, which is 1.4 times higher than the highest efficiency reported so far for PCNF (see Supplementary Note 2, Supporting Information). The platform loaded with 120 mg of the photocatalyst gives the most balanced performance in terms of photocatalyst utilization (0.30 mmol *g*_cat_ **^−^** ^1^ h**^−^** ^1^) and solar energy utilization (0.28%).

## 2 Synthesis and Characterization of the Photocatalysts

**Figure 1:**
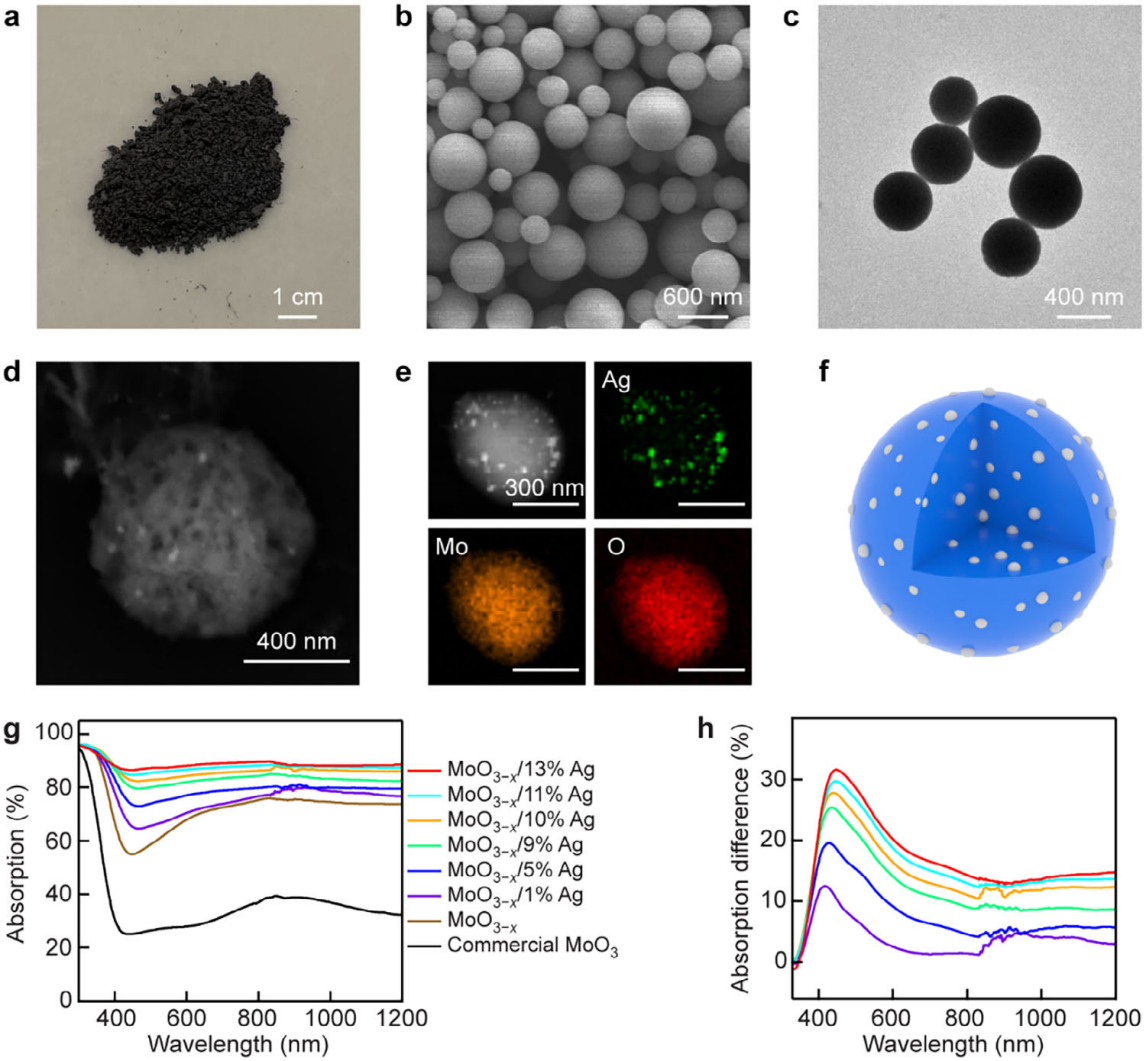
Structural and optical characterization. a) Photograph of the MoO_3−_ *_x_*/10% Ag photocatalyst. b) SEM image of the MoO_3−_ *_x_*/10% Ag nanospheres. c) TEM image of the MoO_3−_ *_x_*/10% Ag nanospheres. d) High‐magnification backscattered electron image of a MoO_3−_ *_x_*/10% Ag nanosphere. e) HAADF‐STEM image (top left) and EDX elemental mapping images of a MoO_3−_ *_x_*/10% Ag nanosphere. f) Schematic illustrating the structure of a MoO_3−_ *_x_* nanosphere loaded with Ag nanoparticles. g) Light absorption spectra of commercial intrinsic MoO_3_ nanoparticles and the MoO_3−_ *_x_*/Ag samples with 0, 1, 5, 9, 10, 11, and 13% Ag. h) Light absorption difference spectra of the MoO_3−_ *_x_*/Ag samples with 1, 5, 9, 10, 11, and 13% Ag. The difference spectra were calculated relative to the absorption spectrum of the MoO_3−_ *_x_* sample.

Plasmonic MoO_3−_ *_x_*/Ag nanospheres were synthesized facilely by aerosol spray (see the Experimental Section and Figure S2, Supporting Information). The loading amount of Ag nanoparticles was adjusted by varying the concentration of the precursor. The anoxic atmosphere in the tube furnace ensured the formation of Ag nanoparticles and MoO_3_ nanospheres with rich oxygen vacancies (OVs). The color of the collected nanospheres gradually deepens from blue to black with increasing Ag concentrations (**Figure** **1**a; Figure S3, Supporting Information), illustrating a significantly improved light absorption ability. A typical spherical shape was observed for the products under scanning electron microscopy (SEM, Figure 1b; Figure S4, Supporting Information) and transmission electron microscopy (TEM, Figure 1c). The sizes of the nanospheres vary in the range of a few hundred nanometers, with the average diameters ranging from 530 to 588 nm (Figure S5, Supporting Information). The contents of the Mo, O, and Ag elements were determined to evaluate the OV densities and Ag amounts based on the energy‐dispersive X‐ray (EDX) spectroscopy (Figure S6, Supporting Information). The average *x* values in MoO_3−_ *_x_*/Ag are ≈0.55, implying an OV percentage of ≈20%. The measured Ag molar percentages are consistent with those supplied in the precursor solutions. X‐ray photoelectron spectroscopy (XPS, Figure S7, Supporting Information) verified the oxidation states of Ag and Mo to be 0 and +4.89, indicating the successful loading of Ag nanoparticles and the existence of OVs. However, Ag nanoparticles are invisible on the SEM and TEM images (Figure 1b,c), which can be attributed to the small Ag nanoparticle sizes and the indistinguishable contrast originating from the similar atomic numbers between Mo and Ag.

To verify the successful loading of Ag nanoparticles in the MoO_3−_ *_x_* nanospheres, high‐magnification SEM imaging at the backscattered electron detection mode (Figure 1d), high‐angle annular dark‐field scanning transmission electron microscopy (HAADF‐STEM), and EDX elemental mapping (Figure 1e) were performed. A clear contrast between the Ag nanoparticles and the MoO_3−_ *_x_* nanospheres was observed. X‐ray diffraction (XRD) was also performed to determine the existence of Ag nanoparticles (Figure S8 and Table S1, Supporting Information), with typical Ag peaks observed at ≈38° and ≈44°. Based on the characterization results above, Ag nanoparticles should be dispersed randomly close to the surface of and inside the nanosphere.^[^ ^34^, ^35^ ^]^ A schematic is therefore provided for an individual MoO_3−_ *_x_*/Ag nanosphere to illustrate the structure of the photocatalyst nanoparticles (Figure 1f).

The existence of OVs and Ag nanoparticles is also reflected in the absorption spectra. The OV‐induced abundant electron charge carriers create strong LSPR (Figure S9, Supporting Information), which covers a broad range from the visible to the NIR region (Figure 1g).^[^ ^23^ ^]^ The absorption dip at ≈450 nm of the MoO_3−_ *_x_* nanospheres becomes significantly shallower with the incorporation of more Ag nanoparticles. The elimination of the absorption dip is caused by the LSPR of the Ag nanoparticles at ≈466 nm, as revealed by the calculated absorption difference spectra (Figure 1h). The redshifted plasmon peak of the Ag nanoparticles compared to that in aqueous solution is induced by the larger refractive index of MoO_3−_ *_x_* than that of water.^[^ ^36^ ^]^ The plasmon peak of the Ag nanoparticles redshifts gradually with increasing Ag amounts, which can be attributed to the fact that the Ag nanoparticles become larger. Electron paramagnetic resonance, steady‐state photoluminescence spectroscopy, and time‐resolved fluorescence decay measurements (Figure S10, Supporting Information) were further implemented to prove the existence of OVs, which play an essential role in N_2_ bond activation and charge carrier recombination inhibition.^[^ ^37^ ^]^

The Schottky‐barrier‐free nature, optical bandgaps, flatband potentials, charge carrier densities, and impedance of the samples were determined from Kelvin probe force microscopy measurements (Figure S11, Supporting Information), Tauc plots, Mott–Schottky plots, and electrochemical impedance plots (Figure S12 and Tables S2 and S3, Supporting Information), respectively. The Ag loading significantly improves the free charge carrier density and reduces the charge‐transfer resistance, which is beneficial to the generation and utilization of plasmonically generated electron–hole pairs. The specific band structures of Ag and MoO_3−_ *_x_* were thereby calculated (Figure S12d, Supporting Information). The higher working function of Ag than the conduction band (CB) edge of MoO_3−_ *_x_* causes the CB edge to bend downward at the interface (Figure S1, Supporting Information), which makes the Ag–MoO_3−_ *_x_* interface Schottky‐barrier‐free. The Schottky‐barrier‐free feature ensures the free transportation of plasmonic charge carriers, making them readily accessible for redox reactions. Moreover, the CB edge position is slightly elevated upon Ag loading, which is beneficial for the N_2_ reduction reaction.

## 3 PCNF Performance of the Photocatalysts

The PCNF experiments were performed using a home‐built photocatalytic system (Figure S13, Supporting Information) and measured using Nessler's method (Figure S14, Supporting Information). The reaction conditions were examined through the photocurrent response (Figure S15, Supporting Information) and control experiments (Figure S16, Supporting Information). Pre‐bubbling of N_2_ before light illumination for 40 min, together with stirring, was carried out for the sufficient dissolution of N_2_, the even distribution of the photocatalyst powder in the water solution, and the desorption of NH_3_ that was originally adsorbed in the photocatalyst nanoparticles. PCNF cannot proceed without any of the following conditions: light illumination as the energy source, N_2_ as the N element source, or water as the proton source. The reaction products contain NH_3_, O_2_, and H_2_, with the selectivity toward PCNF being ≈86.6% (Table S4, Supporting Information).

**Figure 2:**
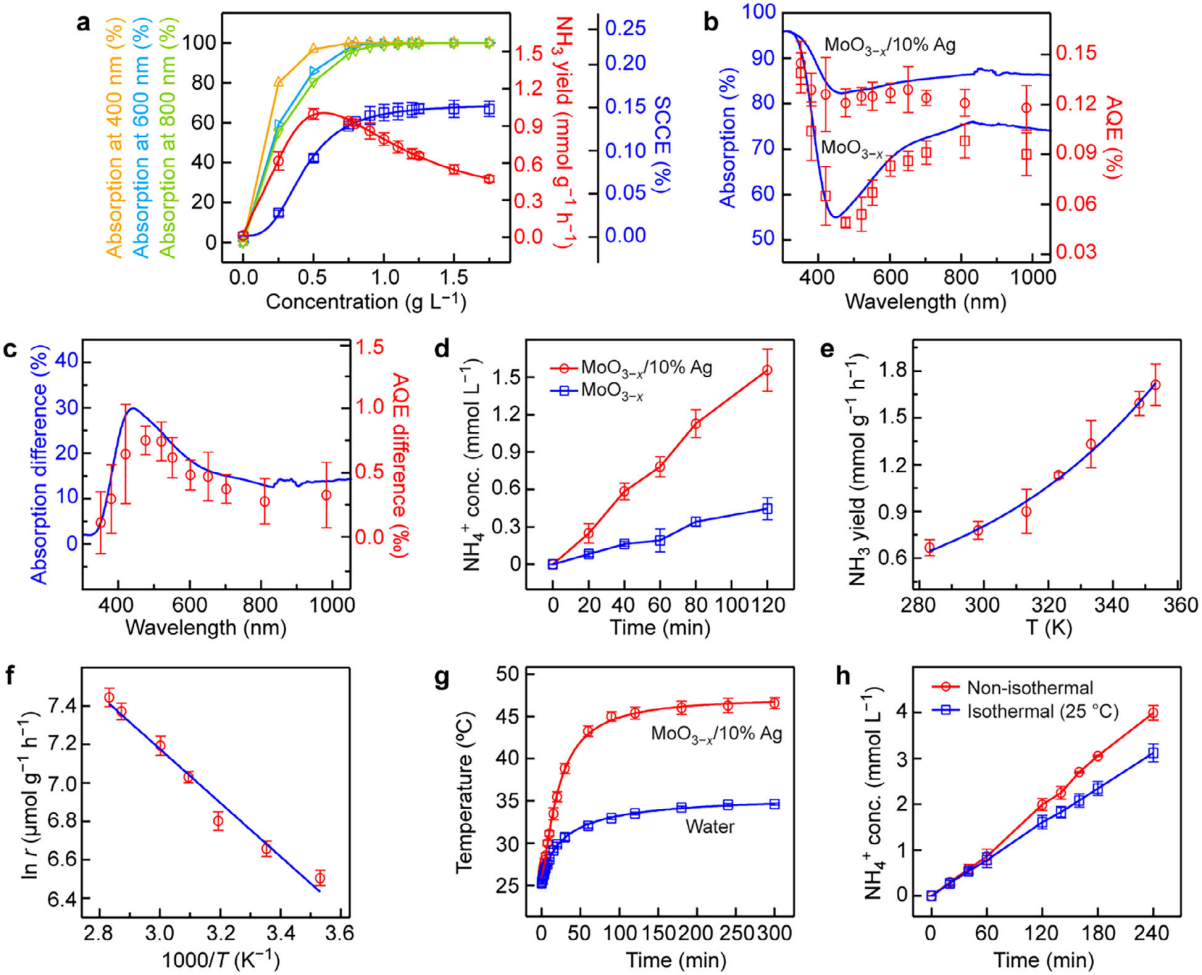
PCNF performances of the MoO_3−_ *_x_*/10% Ag sample. a) Effect of the photocatalyst concentration on the light absorption at 400, 600, and 800 nm (the left three axes), the NH_3_ yield (the first right axis), and the SCCE (the second right axis) for the MoO_3−_ *_x_*/10% Ag sample. b) Light absorption (left axis) and measured AQE (right axis) spectra of the MoO_3−_ *_x_* and MoO_3−_ *_x_*/10% Ag samples under the optimized conditions. c) Light absorption difference (left axis) and AQE difference (right axis) of the MoO_3−_ *_x_*/10% Ag sample with respect to the MoO_3−_ *_x_* sample under the optimized conditions. d) Time‐dependent production of NH_3_ for the MoO_3−_ *_x_*/10% Ag sample under the optimized conditions. e) Effect of the environmental temperature on the NH_3_ yield. f) Arrhenius plot for the NH_3_ yield and temperature. g) Time‐dependent temperature variations under non‐isothermal conditions. h) Time‐dependent production of NH_3_ under non‐isothermal and isothermal conditions. All experiments in (e–h) were performed under optimized conditions.

In comparison with MoO_3−_ *_x_*, the PCNF activities of the MoO_3−_ *_x_*/Ag samples are significantly boosted, with the maximum located at ≈10% Ag loading amount (Figure S17, Supporting Information). Higher loading amounts of Ag may increase the sizes of the Ag nanoparticles, which will reduce the plasmonic generation efficiency of hot carriers because of the increased scattering contribution to the extinction of incident light. The MoO_3−_ *_x_*/10% Ag sample was therefore selected for further investigation. The photocatalyst concentration in the water solution is vital for sufficient light absorption. A low concentration will cause incomplete solar energy utilization, while a high concentration will lead to the waste of the photocatalyst (Figure S18a,b, Supporting Information). The specific effect of the photocatalyst concentration on the light absorption, the NH_3_ yield, and the SCCE was therefore investigated (**Figure** **2**a; Figure S18c, Supporting Information). The light absorption values at three wavelengths were selected to characterize the overall light absorption abilities of the photocatalyst in aqueous solutions. The SCCE displays a similar trend to that of the light absorption, while the NH_3_ yield shows a different trend. This phenomenon can be attributed to the fact that the NH_3_ yield is normalized against the mass of the photocatalyst and is closely related to the amount of the photocatalyst. There exists an optimal concentration for a given photocatalyst. Too high concentrations will lead to the waste of the photocatalyst. However, the SCCE characterizes the photocatalytic ability of the entire solution. With increasing photocatalyst concentrations, the light absorption ability increases and gets saturated. To balance the two parameters, 1 g L^−1^ photocatalyst concentration, giving a high SCCE and a relatively high NH_3_ yield, was selected for further experiments. Other reaction parameters were then investigated in Figure S19 (Supporting Information), which gives rise to the optimized reaction conditions as: 10 sccm N_2_ flow rate during the reaction, and 5,000 rpm stirring speed.

The apparent quantum efficiency (AQE) and SCCE values were measured under the optimized conditions. The AQEs measured at different wavelengths exhibit a remarkable correlation with the light absorption spectra (Figure 2b; Table S5, Supporting Information). The different trends between the AQEs of the two photocatalysts (Figure 2c) also perfectly match the Ag absorption peak, indicating that the loaded Ag nanoparticles effectively work as light absorbers and photoelectron generators in the region at ≈466 nm for PCNF. The best PCNF performance with an NH_3_ yield of 0.78 (±0.08) mmol *g*_cat_^−1^ h^−1^ and a SCCE of 0.14% (±0.01%) was achieved (Figure 2d; Table S6, Supporting Information).

A fraction of hot electron–hole pairs generated through the Landau damping of LSPR can be utilized for redox reactions, while the remainder will undergo recombination, and their energy will finally dissipate to the environment as heat.^[^ ^38^, ^39^, ^40^ ^]^ The temperature effect was therefore investigated to further enhance the PCNF performance. The Arrhenius plot was obtained by setting the PCNF reaction at different temperatures and fixing the light illumination conditions (Figure 2e,f). With the assistance of incident photons, the apparent activation energy was determined to be 11.6 kJ mol^−1^. Specifically, the plasmonic photothermal conversion effect of the photocatalyst heated the reactor to ≈45° in 1 h, and the system reached a dynamic temperature equilibrium with the environment after 2 h (Figure 2g). Under this circumstance, the entire PCNF reaction exhibited accelerated reaction kinetics, resulting in an even higher PCNF performance with an elevated SCCE of 0.18% (±0.03%). The value represents a 28% increase in comparison with that achieved under the isothermal reaction conditions. Such a high SCCE suggests the superior solar energy utilization ability of the photocatalyst (Figure 2h; Table S7, Supporting Information).

A part of the synthesized MoO_3−_ *_x_*/10% Ag nanospheres was inevitably wasted during the reaction and recycling processes. The stability of the MoO_3−_ *_x_*/10% Ag sample was examined by reweighing the post‐reaction photocatalyst before each run. The NH_3_ yield retained ≈83% after 4 cycles (Figure S20a,b, Supporting Information). The surface morphology, crystalline structure, and existence of OVs were found to be nearly unchanged after 4 cycles (Figure S20c–e, Supporting Information). The XPS spectra revealed that the Ag nanoparticles were well protected from being oxidized, and that MoO_3−_ *_x_* was slightly oxidized (Figure S21, Supporting Information). To further assess the long‐term stability, the cycling tests were extended to 10 cycles (≈20 h). The overall structure and morphology remained stable, while the PCNF activity decreased by ≈40% (Figure S22, Supporting Information). Inductively coupled plasma mass spectrometry measurements suggested that ≈0.72% of Ag nanoparticles were lost in each cycle (Table S8, Supporting Information). Taken together, these results indicate that the MoO_3−_ *_x_*/10% Ag sample possesses relatively good chemical stability for PCNF, which, however, needs to be improved further for practical applications.

## 4 Synthesis and Characterization of the Solar Absorber Films

**Figure 3:**
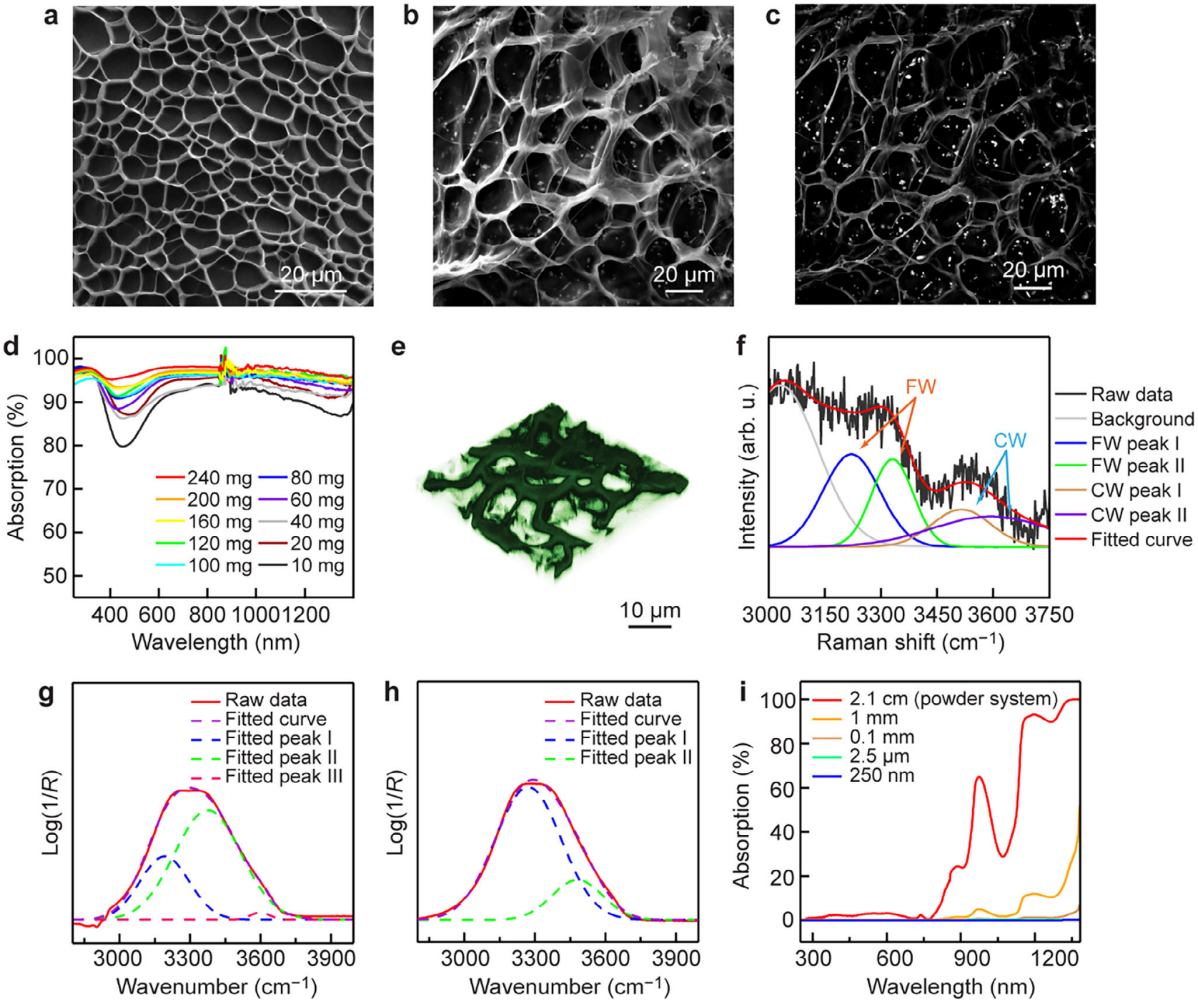
Structure and optical characterization of the solar absorber films. a) SEM image of a pure PVA film. b) High‐magnification SEM image of a solar absorber film obtained with the secondary electron detection mode. c) High‐magnification SEM image of the solar absorber film obtained with the backscattered electron detection mode at the same position as that in (b). d) Absorption spectra of the solar absorber films with various photocatalyst loading amounts. e) 3D reconstructed CLSM image of the solar absorber film. f) Raman spectrum with the fitting peaks representing free and confined water molecules in the solar absorber film. g) ATR‐FTIR spectra of the solar absorber film. h) ATR‐FTIR spectra of bulk water. i) Light absorption of water at different wavelengths in the powder and film systems. The thickness means the adsorbed water layer thickness.

To further improve the light delivery efficiency and utilization ability, the MoO_3−_ *_x_*/10% Ag nanospheres were immobilized into a porous PVA substrate with a pore volume percentage of ≈51% at varied amounts by freeze‐drying to form MoO_3−_ *_x_*/10% Ag/PVA solar absorber films (Figure S23, Supporting Information). The porous and interlaced structure of PVA (**Figure** **3**a; Figure S24a–c, Supporting Information) provides a vast specific surface area to immobilize the MoO_3−_ *_x_*/10% Ag nanospheres (Figure 3b; Figure S24d–f, Supporting Information). To further confirm the presence of the MoO_3−_ *_x_*/10% Ag nanospheres within the structure, high‐magnification SEM images obtained with the secondary and backscattered electron detection modes were carried out (Figure 3b,c; Figure S25, Supporting Information). High contrast was observed between the MoO_3−_ *_x_*/10% Ag nanospheres and PVA under backscattered electron detection. The existence of Ag nanoparticles was further verified by EDX and XRD measurements (Figures S26 and S27, Supporting Information).

The color of the solar absorber film gradually deepened as the photocatalyst loading amount was increased (Figure S28, Supporting Information), which was also reflected in the absorption spectra (Figure 3d). On the one hand, as PVA has a negligible intrinsic light absorption in the visible–NIR region (Figure S29a, Supporting Information), the absorption peaks in the NIR region for the PVA film are attributed to the presence of water in the film. A thick film held much water, exhibiting strong light absorption in the NIR region, while a thin film accommodated little water, giving negligible light absorption in the NIR region. However, a thin film has limited mechanical stability and photocatalyst loading capacity, which limits photocatalytic applications. On the other hand, the light absorption ability of the photocatalyst nanospheres was found to be lower than that in the solar absorber film (Figure S29b, Supporting Information). This discrepancy can be attributed to the unique advantage of the porous structure of the solar absorber film in terms of re‐absorbing the light scattered away from the photocatalyst nanospheres. All of these results collectively illustrate that a thin solar absorber film with a high photocatalyst loading amount is beneficial to the efficient harvesting of solar energy.

The strong hydrophilicity of the solar absorber film (Figure S30, Supporting Information), along with the presence of a large specific surface area, facilitates the penetration of water from the bottom to the top of the film through large surface tension, ensuring sufficient mass transfer of water molecules during the PCNF reaction. To investigate the interaction between water molecules and the solar absorber film, confocal laser scanning microscopy (CLSM) was performed (Figure S31, Supporting Information). The cross‐sectional fluorescence images revealed a porous interlaced network structure, with the surface being coated by an adsorbed water layer. The 3D reconstructed images (Figure 3e; Figure S32, Supporting Information) further vividly illustrated the presence of an adsorbed water layer and a large number of cavities for air accommodation. Specifically, the reason for the formation of a thin water layer is the substantial amount of hydroxyl groups on the surface of the PVA network (Figure S33, Supporting Information), which causes the formation of abundant hydrogen bonds with water molecules. To investigate the different types of water molecules, the Raman spectrum of the solar absorber film (Figure 3f; Figure S34, Supporting Information) was fitted using four Gaussian peaks, which were located at 3233, 3401, 3514, and 3630 cm^−1^, respectively.^[^ ^41^, ^42^ ^]^ The peaks of free water are located at 3233 and 3401 cm^−1^. They correspond to water molecules forming four hydrogen bonds with adjacent water molecules or hydrophilic groups, where two protons and two lone electron pairs are involved in hydrogen bonding with adjacent water molecules. On the other hand, the peaks at 3514 and 3630 cm^−1^ represent the confined water with fewer hydrogen bonds, including weakened water–water hydrogen bonding (intermediate water) and water–PVA hydrogen bonding (bound water) (Figure S35, Supporting Information).^[^ ^42^, ^43^, ^44^ ^]^ The porous structure of the solar absorber film provides ample air capacity, offering numerous opportunities for N_2_ molecules to contact the surface of the photocatalyst nanoparticles.

The thickness of the adsorbed water layer was determined from Fourier‐transform infrared (FTIR) spectroscopy with an attenuated total reflection (ATR) accessory^[^ ^45^, ^46^, ^47^ ^]^ to be ≈234.2 nm (Figure 3g,h). The corresponding light attenuation caused by the water layer at various light wavelengths was thereby calculated according to the Beer–Lambert Law (Table S9, Supporting Information) and plotted in Figure 3i. For the photocatalyst loaded at the upper surface of the film, the water layer thickness is ≈250 nm, leading to minimal light absorption by water in the entire solar light spectrum (<0.1%). For the photocatalyst loaded at the bottom of the film, the thickness of the water layer is nearly equal to that of the entire film (≈1 mm), resulting in greatly suppressed light attenuation by water compared to that in the powder system.

## 5 PCNF Performance of the Solar Absorber Films

**Figure 4:**
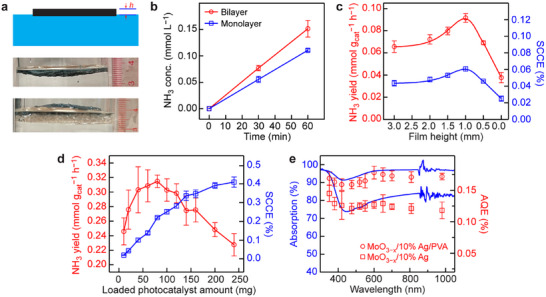
PCNF performances of the solar absorber films. a) Interface structure between the solar absorber film and water. From top to bottom: the ideal interface, monolayer interface, bilayer interface. b) Time‐dependent production of NH_3_ for the monolayer and bilayer systems. c) Effect of the height of the film above the water surface on the NH_3_ yield (left axis) and the SCCE (right axis) for the solar absorber film. d) Effect of the photocatalyst loading amount on the NH_3_ yield (left axis) and the SCCE (right axis) for the solar absorber films with 1 mm thickness. e) Light absorption (left axis) and measured AQE (right axis) spectra of the powder and film systems.

Benefitting from such a thin water layer, air with a humidity level of ≈50% and a N_2_:O_2_ volume ratio of ≈3:1 can be directly employed as the feeding gas (Figure S36, Supporting Information). The porous structure provides a large number of pores that can accommodate air meshes. N_2_ molecules in such air meshes only need to penetrate the thin water layer with ≈250 nm thickness to reach the surfaces of the photocatalyst nanoparticles. N_2_ molecules can be continuously consumed in the reaction while being quickly replenished from air, which is more direct and convenient than that in conventional slurry reactors. The other reaction conditions were further verified by the rigorous control experiments (Figures S37 and S38, Supporting Information). The experimental setup is shown in **Figure** **4**a. The upper part of Figure 4a illustrates the ideal configuration with the majority of the film being exposed above the water surface. However, in the practical setup as shown in the middle part of Figure 4a, the solar absorber film can only float on water with the main body being submerged below the water surface. A foam cushion with the proper size was therefore employed to realize the ideal configuration (Figure 4a, bottom). The majority of the film was elevated out of the water surface, with only a small part of the film staying in contact with water for spontaneous transfer of water molecules from the bottom to the top region. The necessity of incorporating the foam to create a bilayer structure was further verified by performing the PCNF reaction. The performance was significantly boosted in the bilayer system (Figure 4b), giving a ≈30% improvement in the photocatalytic NH_3_ production.

To better understand the effect of the height of the film above the water surface on the photocatalytic performance, the foam cushion was replaced by a thick Cu cylinder as the support. A solar absorber film with a thickness of 3 mm was chosen, and the height of the film above the water surface was systematically varied from 0.0 to 3.0 mm (Figure S39 and Table S10, Supporting Information). When the height of the film above the water surface reached 1 mm, the entire photocatalytic system was found to exhibit the highest NH_3_ yield and SCCE (Figure 4c; Figure S40a, Supporting Information). Therefore, after considering the factors of the light absorption by water, mechanical and loading ability, and catalytic activity, we selected the solar absorber film with a thickness of 1 mm for further experiments.

The photocatalyst powder was then loaded on the solar absorber film at varied amounts, and the photocatalytic activities were evaluated (Figure S40b and Table S11, Supporting Information). Similarly, the determined NH_3_ yields show a volcano dependence on the photocatalyst loading amount (Figure 4d), with the maximum located at ≈80 mg. The NH_3_ yields of the film systems are lower than those of the powder systems, which can be attributed to the partial coating of the surface of the photocatalyst nanoparticles by PVA and the deactivation of the photocatalyst during the film preparation process. However, the SCCEs are much higher than those in the powder system, with the maximal value reaching 0.41% (±0.03%) when 240 mg of the photocatalyst was loaded in the film. This value, to the best of our knowledge, is the highest among those that have been achieved on the world, demonstrating an ultrahigh solar energy utilization of the photocatalyst in the film system. In all, the film system demonstrates a brilliant PCNF activity with the highest NH_3_ yield of 0.31 (±0.01) mmol *g*_cat_ **^−^** ^1^ h**^−^** ^1^ and the highest SCCE of 0.41% (±0.03%) achieved at different photocatalyst loading amounts. To balance the two parameters, 120 mg photocatalyst loading amount giving a relatively high NH_3_ yield of 0.30 (±0.01) mmol *g*_cat_ **^−^** ^1^ h**^−^** ^1^ and SCCE of 0.28% (±0.01%) was selected for subsequent investigations.

At last, the AQEs were measured to illustrate the superiority of the film system (Figure 4e; Table S12, Supporting Information). All AQEs of the film system are higher than those of the powder system, illustrating the superior solar energy delivery and utilization across the entire measurement range. Furthermore, the solar absorber film system exhibits a negligible photothermal effect (Figure S41, Supporting Information) and relatively good chemical stability (Figure S42, Supporting Information) throughout the 9 h PCNF reaction.

## 6 Conclusion

In summary, a Schottky‐barrier‐free plasmonic MoO_3−_ *_x_*/Ag photocatalyst has been designed to effectively reduce N_2_ to NH_3_ under ambient conditions. The superior light absorption ability in a broad range from the ultraviolet to the NIR region, the greatly enhanced charge carrier utilization efficiency brought by the Schottky‐barrier‐free feature, the N_2_ chemisorption and activation ability by the abundant OVs, and the accelerated reaction kinetics by the plasmonic photothermal conversion effect synergistically boost the PCNF performance, giving a SCCE of 0.18% (±0.03%). A floatable diphase film is then designed by loading the MoO_3−_ *_x_*/10 mol% Ag (Ag loading amount: 57.98 mg *g*_cat_^−1^) photocatalyst in a hydrophilic PVA film. The film system, located at the air–water interface, significantly suppresses the light absorption of water in the NIR region, enhances the solar energy delivery, and boosts the reaction kinetics of N_2_ mass transfer. Consequently, without additional continuous stirring or forced convection, the solar absorber films exhibit the highest NH_3_ production rate of 0.31 (±0.01) mmol *g*_cat_ **^−^** ^1^ h**^−^** ^1^ and the highest SCCE of 0.41% (±0.03%) with air directly used as the feeding gas. The solar absorber film with 120 mg photocatalyst loading amount gives the most balanced performance, with the NH_3_ yield being 0.30 (±0.01) mmol *g*_cat_ **^−^** ^1^ h**^−^** ^1^ and SCCE being 0.28% (±0.01%). We believe that the proposed diphase reaction platform, with future developments, represents a promising direction for promoting the development of PCNF toward practical applications.

## 7 Experimental Section

### Synthesis of the Plasmonic MoO_3−x_/Ag Samples

The plasmonic MoO_3−_ *_x_*/Ag samples were prepared through an aerosol‐spray process.^[^ ^23^, ^34^, ^35^, ^48^, ^49^ ^]^ In a typical synthesis, H_2_MoO_4_ and CH_3_COOAg with a desired Ag:Mo ratio (Ag:(Ag + Mo) mol%) were sequentially dissolved in deionized water (40 mL) at room temperature. The total metal (Ag and Mo) concentration was kept at a constant value of 0.15 mol L^−1^. The precursor suspension was then transferred into an ultrasonic humidifier to be atomized into aerosol droplets with diameters of ≈3–8 µm.^[^ ^50^ ^]^ The aerosol droplets were driven by an Ar stream and a vacuum pump in a quartz tube (inner diameter: 25 mm, wall thickness: 2.5 mm) to pass through a tube furnace set at 350 °C. When the droplets were passing through the hot zone of the tube furnace, the water content was quickly evaporated while the precursor ingredients were decomposed and crosslinked to form MoO_3−_ *_x_*/Ag nanospheres.

### Synthesis of the MoO_3−x_/10% Ag Solar Absorber Films

The photocatalyst solar absorber films were fabricated by a freeze‐drying method.^[^ ^41^, ^42^ ^]^ For a typical synthesis of a solar absorber film with 1 mm thickness, PVA (0.375 g) and PEG (0.03 g) were first dissolved in deionized water (5 mL) by sonication at room temperature to form a translucent solution. The solution was further mixed with glutaraldehyde solution (46.88 µL) under ultrasonication and named solution A. Simultaneously, the photocatalyst powder with appropriate amounts (0–240 mg) was added into deionized water (295.6 µL) and sonicated to form a colloidal solution. The colloidal solution was then mixed with HNO_3_ (10 wt%, 204.4 µL) to form solution B. The solutions A and B were subsequently mixed in a beaker (50 mL). The mixture was sonicated and heated up in a water bath with a heating rate of 10 °C min^−1^ and kept at 70 °C for 30 min to initiate polymerization, yielding a gel film. The same beaker was used to fabricate other solar absorber films with different thicknesses, and the amounts of the raw materials were proportionally increased. The films were then transferred into the frozen layer of the refrigerator for 10–12 h to ensure that the liquid water was completely transformed into solid ice. The frozen products were finally placed under a smooth and flat weight and transferred to the freeze‐drying machine (TL‐FD‐1A‐50, Jiangsu Tianling Instrument Co., China) for two days until the ice was removed by sublimation under vacuum at a low temperature. The function of the weight was to keep the film flat during the freeze‐drying process. The final product was a dry porous cake with a high surface area and a low moisture content, which can be easily varied by immersing the product in water.

### ATR‐FTIR Experiment

To probe the adsorbed water molecules and measure the thickness of the thin water layer, a Thermo Nicolet iS 50 FTIR spectrometer equipped with an ATR accessory setup was employed to collect the spectra of the water layers in bulk water and on the solar absorber films. Specifically, the ATR component was composed of a diamond hemispherical ATR crystal with a 45° bevel‐cut and polished at both ends for IR entrance and exit. The diamond crystal was cleaned before each run to avoid contamination. The background spectrum was acquired using a clean diamond under an air atmosphere. During each run, deionized water (10 µL) was first dripped on the surface of the diamond. The sample was then put upright. The water layer spectra of the sample were acquired by taking the absorption spectrum against the background spectrum. The effective thickness of water for analysis on this instrument was ≈300 nm.^[^ ^51^ ^]^

The thickness of the adsorbed water layer (*d*) can be estimated by comparing the integrated areas of the adsorbed water (*A*_ads_) of the solar absorber film with the peak area of the bulk water (*A*_bulk_) when the penetration depth (*d*_bulk_) of the evanescent infrared beam is taken into account^[^ ^52^ ^]^

(1) $$d=\frac{A_{\mathrm{ads}}}{A_{\mathrm{bulk}}}d_{\mathrm{bulk}}=\frac{A_{\mathrm{ads}}}{A_{\mathrm{bulk}}}\times \frac{\lambda }{2\pi {\left(n_{1}^{2}\mathrm{si}n^{2}\theta -n_{2}^{2}\right)}^{0.5}}$$

where *λ* is the wavelength of the infrared beam, *θ* is the incidence angle of the beam (45°), and *n*_1_ and *n*_2_ are the refractive indexes of the substrate and bulk water, respectively. The exact refractive index of the adsorbed water layer is assumed to be equivalent to that of bulk water.

## Conflict of Interest

The authors declare no conflict of interest.

## Author Contributions

J.T.H. and J.F.W. conceived the project. J.T.H., K.A., Y.Z.G., and X.P.B. performed the photocatalyst synthesis, film fabrication, and PCNF experiments. J.T.H., Y.F.R., and F.R.W. performed the structure characterization. J.T.H., K.A., X.P.B., D.G.W., and J.F.W. wrote the manuscript with comments from all authors. J.F.W. and D.G.W. supervised the project. All authors discussed the results and contributed to the writing of the manuscript.

## Supporting information



Supporting Information

## Data Availability

The data that support the findings of this study are available from the corresponding author upon reasonable request.
