# Supplementary material for: Plasmonic MoO3− x /Ag Photocatalyst for the Fixation of N2 from Air with the Solar Energy Conversion Efficiency Reaching over 0.28%
Source: Adv Mater. 2025 Aug 11;37(43):e09652. doi: 10.1002/adma.202509652 (PMC12574637; doi:10.1002/adma.202509652)
Supplement: Supplementary file 1 — Supporting Information [file ADMA-37-e09652-s001.docx]

Supporting Information

**Plasmonic MoO_3–_*_x_*/Ag Photocatalyst for the Fixation of N_2_ from Air with the Solar Energy Conversion Efficiency Reaching over 0.28%**

*Jingtian Hu, Ke An, Yifei Ren, Feng Ryan Wang, Yanzhen Guo, Xiaopeng Bai,* Degao Wang,* and Jianfang Wang**

**Experimental Section**

*Chemicals*: Silver acetate (CH_3_COOAg) was purchased from Macklin. Molybdic acid (H_2_MoO_4_) was acquired from Acros Organics. Glutaraldehyde solution (C_5_H_8_O_2_, 50% in water) and nitric acid (HNO_3_, 68 wt%) were obtained from Aladdin. Polyvinyl alcohol (PVA, molecular weight: 13,000–23,000, 87%–89% hydrolyzed) was purchased from Aldrich. Polyethylene glycol (PEG, BioUltra, molecular weight: 4,000) was purchased from Sigma. Deionized water with a resistivity of 18.2 MΩ cm was used throughout the experiments.

*Characterization*: Scanning electron microscopy (SEM, FEI Quanta 400 FEG) was employed for the sample morphology characterization. The operation pressure of the SEM was 5.0 × 10^−5^ torr. The electron beam was generated by a field-emission gun operated at 10–20 kV. The atomic ratios were analyzed by the energy-dispersive X-ray (EDX) spectroscopy unit attached on the SEM system. The high-magnification backscattered electron (BSE) images were acquired with the backscattered electron detection mode attached to the SEM system. Transmission electron microscopy (TEM, FEI Tecnai Spirit 12) imaging was operated at 120 kV. High-angle annular dark-field scanning transmission electron microscopy (HAADF-STEM) characterization and elemental mapping were performed on another TEM system (FEI Tecnai F20) operated at 200 kV and equipped with an Oxford EDX system. The X-ray photoelectron spectroscopy (XPS) measurements were performed on a Thermo Fisher Escalab 250Xi system with all binding energies calibrated using contaminant carbon (C 1s at 284.6 eV) as the reference. The X-ray diffraction (XRD) patterns were recorded on an X-ray diffractometer (RU-300, Rigaku) equipped with Cu Kα radiation (wavelength: 1.5406 Å) under ambient conditions in air. The loading amount of Ag nanoparticles and the content of Mo in the catalyst sample were determined by inductively coupled plasma mass spectrometry (ICP-MS, Agilent 720). Prior to the ICP-MS measurements, the photocatalyst sample was dissolved by a mixture solution of nitric acid, hydrofluoric acid, and hydrogen peroxide at 200 ℃ for 1 h. The linear calibration curves were obtained from the corresponding salt solutions of known concentrations.

The diffuse reflectance absorption spectra were measured on an ultraviolet/visible/near-infrared spectrophotometer (PerkinElmer, Lamda 950) equipped with an integrating sphere accessory using BaSO_4_ as the reference. The sample powder was deposited on, or the film was attached to a BaSO_4_ substrate for the diffuse reflectance (%*R*) spectrum measurement. The light absorption (%*A*) was obtained according to the equation

$\%A=100-\%R$ (1)

The energy bandgap was calculated according to the *Tauc* relationship

$\alpha hv=B(hv-E_{g})^{a}$ (2)

where $hv$ is the incident photon energy, *B* is a constant, *E*_g_ is the bandgap to be determined, and *a* is a constant taking the value of 1/2 for MoO_3_. The absorption coefficient *α* was calculated according to the equation

$\alpha=\frac{1}{d}\ln[\frac{1}{1-(A/100)}]$ (3)

In our experiments, the thickness *d* of the sample was 2 mm. *A* is the light absorption (in percentage) measured at various wavelengths. The optical bandgap of each sample was determined from the intersection of the linear extrapolations above and below the knee energy point.

The single-particle dark-field scattering measurements were performed on a home-built optical system based on an upright optical microscope (Olympus, BX60). The system was equipped with a quartz-tungsten-halogen lamp (100 W), a monochromator (Acton, SpectraPro 2360i), and a charge-coupled device camera (Princeton Instruments, Pixis 400, cooled to −70 ℃).^[1,2]^ A 100× dark-field objective (Olympus) with a numerical aperture of 0.9 was used for both incident light concentration and scattered light collection.

The electron paramagnetic resonance (EPR) spectra were acquired on a Bruker E580 X-band spectrometer equipped with a Bruker ER4122-SHQE cavity under ambient conditions. The steady-state photoluminescence (PL) spectra were measured on a Hitachi F-4600 spectrophotometer at an excitation wavelength of 370 nm. The time-resolved fluorescence decay spectra were acquired on an FS980 spectrometer at an excitation wavelength of 366 nm and an observation wavelength of 435 nm.

The Kelvin probe force microscopy (KPFM) measurements were performed on a system (SPM-9700HT) with an alternating current voltage applied to the tip. A 300 W xenon lamp with an AM 1.5 filter was employed as the light source. The potential of Au (*V*_Au_) was first measured as the reference. The workfunction of the sample (*ϕ*_sample_) was calculated according to the equation

$e(V_{\mathrm{tip}}-V_{\mathrm{Au}})=\phi_{\mathrm{Au}}-\phi_{\mathrm{sample}}$ (4)

where *e* is the electron charge, *V*_tip_ is the tip potential of the sample under different conditions, and *ϕ*_Au_ is the workfunction of the Au reference, with a typical value of 5.1 eV.

The light absorption spectra of pure water and the MoO_3–_*_x_*/10% Ag aqueous solutions were recorded on an ultraviolet/visible/near-infrared spectrophotometer (PerkinElmer, Lamda 950) equipped with a two-dimensional optical detector using air as a reference. For the measurement of pure water, pure water was poured into a quartz cuvette with 1 cm path length. For the measurement of the aqueous photocatalyst solutions, the MoO_3–_*_x_*/10% Ag nanosphere samples were dispersed in water at desired concentrations. The solution was then transferred into a quartz cuvette of 1 cm path length. The light absorbance *A* values of the water layers with different thicknesses in the film systems and powder systems were obtained according to the Beer–Lambert Law

$A=\varepsilon lc$ (5)

where *ε* is the molar absorptivity of the attenuating species, *l* is the optical path length in cm, and *c* is the concentration of the attenuating species. The optical path length of the water layer in the powder system was measured to be 2.1 cm, while that in the film system varied from 250 nm to 1 mm. The absorption (%*A*) values of the water layer at specific wavelengths were calculated according to the equation

$\%A=\left( 1-{0.1}^{A} \right)\times100\%$ (6)

The light absorbance *A* and the absorption (%*A*) of the solution at specific wavelengths in the photocatalytic reactor were obtained using the same method.

The water contact angles were measured using a contact angle system (DCAT21). The photographs were taken 5 s after the water droplets were dripping down. The confocal laser scanning microscopy (CLSM) measurements were conducted using a Dragonfly 200 multi-modal confocal system (Leica SP8). The solar absorber films were first cut into small pieces (1 × 1 cm^2^) for better observation. A rhodamine 640 dye-labelled aqueous solution (0.1 mM, 100 μL) was then deposited onto a confocal dish, followed by placing the solar absorber film at the water–air interface and left undisturbed for 10 min for sufficient infiltration.^[3]^ As the rhodamine 640 dye exhibited an absorption peak centered at 575 nm and a fluorescence peak located at 630 nm, a 575 nm laser was used as the excitation light source and the observation range was set to 590–660 nm. The scan mode was XYZ, and the scan speed was 400 Hz. A 10× objective lens (HC PL FLUOTAR 10×/0.30 DRY) was employed. The three-dimensional reconstruction images were acquired by integrating the cross-sectional fluorescence images, which were continuously taken at different depths by opening the Z-wide option. These fluorescence images were plotted together with a Matlab code. Fourier-transform infrared (FTIR) spectrophotometry (Thermo Nicolet Nexus 670) was also employed to study the chemical contents of the solar absorber films. The solar absorber films were dried in air at 80 ℃ for 2 days to remove adsorbed water. The films were then ground into fine powder right before the FTIR measurements. In a typical process, the sample powder (2 mg) was mixed with KBr (98 mg) and pressed into a pellet of 1.5 cm in diameter for the FTIR measurements. The Raman spectra were measured on a spectrometer (WITec alpha300). The excitation radiation for the Raman emission was produced using an yttrium aluminium garnet laser that had a single-mode operation at 532 nm.

*Electrochemical Measurements*: The electrochemical measurements were performed with a three-electrode system on an electrochemical workstation (CHI 760E, Shanghai Chenhua). The working electrode was prepared by depositing the photocatalyst on a transparent fluorine-doped tin oxide (FTO) glass slide. In a typical process, the photocatalyst was first dispersed in an ethanolic solution of Nafion (5.0 vol%) to form a 10 mg mL^−1^ solution. A portion (0.5 mL) of the dispersion solution was then drop-cast on the conductive surface of the FTO substrate with a surface area of 1 × 1.5 cm^2^. The FTO substrate was then dried in vacuum at room temperature. A Pt plate and the standard Ag/AgCl electrode were employed as the counter and reference electrode, respectively. All measurements were carried out at room temperature in aqueous Na_2_SO_4_ (0.1 M, 80 mL) solutions that had been deoxygenated by bubbling high-purity Ar for 30 min.

The electrochemical impedance spectra (EIS) and the Mott–Schottky curves were measured without the use of any light source or gas. For the EIS measurements, the initial potential *E* was set at the value that was 0.8 V higher than the open-circuit potential. The frequency range was set at 0.01–100,000 Hz. The initial potential *E* and final *E* of the Mott–Schottky curves were set at the values that were 0.8 V higher and lower than the open-circuit potential at a potential step of 0.05 V and a frequency of 1,000 Hz. The interfacial capacitance (*C*) between the working electrode and the electrolyte and the imaginary part (*Z*_i_) of the impedance follows the equation

$C=-\frac{1}{2\pi fZ_{i}}$ (7)

where *f* is the set frequency during the impedance–potential measurement. The flatband potential (*E*_fb_) of the electrode material and the apparent carrier concentration (*n*) were then obtained according to the following relationship^[4]^

$\frac{1}{C^{2}}=\frac{2}{eA^{2}\varepsilon n}(E-E_{\mathrm{fb}}-\frac{kT}{e})$ (8)

where *A* is the interfacial area (1.5 cm^2^) between the working electrode and the electrolyte, *e* is the electron charge, *ε* is the electric permittivity (3.84 × 10^−11^ F m^−1^) of the electrode material at frequency *f*, *n* is the apparent carrier concentration, *E* is the applied voltage, *k* is the Boltzmann constant, and *T* is the temperature in Kelvin.^[5]^ A plot of *A*^2^/*C*^2^ against *E* gave a straight line, from which *E*_fb_ was determined from the intercept on the *E* axis and the electron carrier concentration *n* was calculated from the slope of the straight line.

For the photocurrent measurements, the current between the working electrode and the counter electrode was recorded and plotted against time. The photocurrent measurements were first used to examine the sluggish N_2_ diffusion kinetics. N_2_ or Ar gas was bubbled into the electrolyte solution and switched during the measurements, while the light source was kept turned on and off. The initial potential *E* was set at 1.2 V, the quiet time was set at 20 s, and the sensitivity was set at 10^−6^ A V^−1^. The photocurrent measurements were also employed to acquire the information about photogenerated electrons. In a typical process, N_2_ or Ar gas was bubbled into the electrolyte solution with the light source turned on and off every 20 s. The relative intensity of the individual sample was plotted for better comparison by assuming that the same sample had a similar photocurrent intensity in the dark no matter whether it was in the Ar atmosphere or N_2_ atmosphere.

*Photocatalytic N_2_ Fixation (PCNF) Tests*: The PCNF performance was evaluated in a home-built double-layered photocatalytic reactor. All photocatalytic experiments were performed under simulated solar light (AM 1.5G) illumination without any scavenger under isothermal conditions (25 ℃) unless otherwise specified. Deionized water (80 mL) was first added to the reactor, followed by the addition of an appropriate amount of the photocatalyst or the foam-supported solar absorber film. For the PCNF experiments with the photocatalyst powder, the solution was sonicated for 15 min for even dispersion of the photocatalyst nanoparticles and then bubbled with high-purity N_2_ (purity: ≥99.995%) at a flow rate of 50 sccm for 40 min with continuous magnetic stirring at 5,000 revolutions per minute (rpm). The concentration of NH_3_ at 40 min was selected as the concentration origin. The temperature of the reactor was kept constant at 25 ℃ by an external circulating water system. For the PCNF experiments with the photocatalyst films, the entire system was left undisturbed for 30 min for the self-wetting of the solar absorber film. The concentration of NH_3_ at 30 min was selected as the concentration origin. After the pre-treatment for the powder and film systems, a 300 W xenon lamp (PLS-SXE300C, Beijing Perfectlight Technology) was subsequently turned on. Before light illumination, an AM 1.5G filter was installed on the lamp and the light intensity was adjusted with a pyranometer (KIPP&ZONEN CMP3). The height of the detector of the pyranometer was adjusted to accurately match the top surface of the reaction solution. The light intensity was fixed at 100 mW cm^−2^. The time when the light illumination was turned on was marked as the zero-time point. An aliquot of the reaction solution (1.0 mL) was taken out with a syringe at regular time intervals and then centrifuged at a speed of 5,000 rpm for 8 min. After that, the supernatant (0.5 mL) was collected and the concentration of NH_4_^+^ was determined. The supernatant was diluted with deionized water for high NH_4_^+^ concentrations if needed. Repeated experiments were performed three times for each photocatalyst to obtain average values and standard deviations.

To investigate the effect of the Ag loading amount on the PCNF performance, the photocatalyst (40 mg) was dispersed into deionized water (80 mL), the N_2_ flow rate during the reaction was adjusted to be 10 sccm, and the magnetic stirring speed was fixed at 5,000 rpm. The effect of the photocatalyst concentration on the PCNF performance was investigated under the same N_2_ flow rate and magnetic stirring speed with the MoO_3–_*_x_*/10% Ag sample as the photocatalyst. To explore the effect of the N_2_ flow rate, the MoO_3–_*_x_*/10% Ag sample (80 mg) was dispersed into deionized water (80 mL) to form the photocatalyst solution at 1 g L^−1^ concentration. The magnetic stirring speed was adjusted to 5,000 rpm. The effect of the magnetic stirring speed was finally studied under the following conditions: 80 mg MoO_3–_*_x_*/10% Ag sample, 80 mL deionized water, and 10 sccm N_2_ flow rate.

The AQE spectra were acquired by performing the PCNF experiments under monochromatic light. A 300 W xenon lamp (PLS-SXE300C, Beijing Perfectlight Technology) equipped with bandpass filters at different wavelengths (350, 380, 420, 475, 520, 550, 600, 650, and 700 nm, full width at half maximum values for all: 20 nm, Beijing Perfectlight Technology) and a laser (Diode Laser System, OsTech Electro Optical Instruments, MDL-H-980-5W, PSU-H-LED) were used to create the required monochromatic light. The power intensity of the monochromatic light was determined using a pyranometer (KIPP&ZONEN CMP3) or a power meter (Thorlabs, PM100USB). The AQE was calculated according to

$\mathrm{AQE}=\frac{N_{\mathrm{re}}}{N_{\mathrm{in}}}\times100\%=\frac{3 \times number of the produced NH_{3}\mathrm{molecules}}{E_{\mathrm{in}}\lambda_{\mathrm{in}}/\left( hc \right)}\times100\%$ (9)

where *N*_re_ is the number of reacted electrons, which can be calculated from the molar amount of the produced NH_3_ as each mole of NH_3_ requires 3 moles of electrons participating in the reaction, *N*_in_ is the number of incident photons, *E*_in_ is the optical energy of the monochromatic light, *λ*_in_ is the wavelength of the monochromatic light, *h* is the Planck’s constant, and *c* is the speed of light in vacuum.

Water absorbs light significantly in the NIR region. For the AQEs measured in this region, photons absorbed by water should be deducted. The light absorption of water (*α*_water_) at a certain wavelength was calculated according to

$P_{\mathrm{water}}=P_{0}e^{-\alpha_{\mathrm{water}}L}$ (10)

where *P*_water_ is the transmitted light power after passing through a water layer with a thickness of *L*, and *P*_0_ is the power of the incident light. In our experiments, the thickness of the water layer was that of the quartz cuvette (1 cm). The relationship between *P*_water_ and *P*_0_ was estimated by the following equation

$\frac{P_{\mathrm{water}}}{P_{0}}=1-\%A$ (11)

where $\%A$ is the absorption of the solution measured with an ultraviolet/visible/near-infrared spectrophotometer (PerkinElmer, Lamda 950) equipped with a two-dimensional detector accessory using air as the reference. The light absorption of the photocatalyst (*α*_cat_) was determined in a similar way by replacing air with pure water as the reference.

For a PCNF reaction with a water solution thickness of *L'* (~2.1 cm), the equivalent power of the transmitted light ($P_{\mathrm{water}}^{'}$) after passing through pure water was determined according to Equation (2). The incident light power (*P*_0_) was measured experimentally. The transmitted light power after passing through the photocatalyst solution ($P_{water+cat}^{'}$) was calculated according to the following equation

$P_{water+cat}^{'}=P_{0}e^{-\alpha_{\mathrm{water}}L^{'}}e^{-\alpha_{\mathrm{cat}}L^{'}}$ (12)

The power of the light absorbed by the photocatalyst was thereby determined according to the equation

$P_{\mathrm{cat}}=P_{\mathrm{water}}^{'}-P_{water+cat}^{'}$ (13)

The SCCE was calculated according to the equation

$\mathrm{SCCE}=\frac{{\Delta G}_{\mathrm{ammonia}} n_{\mathrm{ammonia}}}{P_{\mathrm{light}} t}\times100\%$ (14)

where *∆G*_ammonia_ is the change in the Gibbs free energy for NH_3_ generation (339 kJ mol^−1^) with N_2_ and H_2_O as the reactants and NH_3_ and O_2_ as the products, *n*_ammonia_ is the amount of the produced NH_3_, *P*_light_ is the simulated solar light power (100 mW cm^−2^), and *t* is the reaction time.

*Detection of the Photocatalytic Products*: The concentration of the produced ammonia was determined by the chromogenic method with Nessler’s reagent.^[6^^]^ Nessler’s reagent is composed of K_2_HgI_4_ (0.09 mol L^−1^), KOH (2.5 mol L^−1^), and deionized water. It reacts with the produced NH_4_^+^ according to the reaction

NH_4_^+^ + 2[HgI_4_]^2−^ + 4OH^−^ → HgOHg(NH_2_)I + 7I^−^ + 3H_2_O (15)

HgOHg(NH_2_)I is a reddish-brown complex and turns the color of the solution from pale to deep yellow. In a typical process, the analyte solution (0.5 mL) was first mixed with KNaC_4_H_4_O_6_ solution (0.2 mol L^−1^, 0.25 mL), which would minimize the interference of other ions, such as Fe^3+^, Co^2+^, Ni^2+^, Cr^3+^, Ag^+^, and S^2−^, in the subsequent reaction. Nessler’s reagent (0.25 mL) was then added to the mixture, and the entire mixture was left undisturbed for 15 min. The ammonia concentration was determined by monitoring the absorbance at 425 nm according to the pre-established linear relationship between the ammonia concentration and the absorbance on an ultraviolet/visible/near-infrared spectrophotometer. The other products in the gas form (H_2_ and O_2_) were directly measured on a gas chromatograph (CEAULIGHT GC-7900, TCD detector, Ar carrier gas). The production of N_2_H_4_ was detected by a kinetic spectrophotometric determination method.^[7]^

*Temperature Effect in the Powder System*: The temperature effect on PCNF was investigated under the optimal conditions by removing the external circulating water system. The heat generated in the photocatalytic reactor was directly transferred to the surrounding air under ambient conditions. A home-built setup was used to directly monitor the temperature change of the photocatalyst dispersion under simulated solar light illumination. The thermocouple detector was completely immersed in the dispersion solution to characterize the overall heating effect of the plasmonic photocatalyst. In a typical process, the MoO_3–_*_x_*/10% Ag photocatalyst (80 mg) was dispersed into deionized water (80 mL). After sonicating and N_2_ pre-bubbling, the reactor was illuminated by simulated solar light under 10 sccm N_2_ flow and 5,000 rpm magnetic stirring. The produced NH_3_ was probed regularly, while the NH_3_ yields and SCCE values were calculated subsequently.

*Photothermal Effect in the Film System*: The photothermal effect was investigated in the same setup employed for the photocatalytic reactions. A thermocouple detector was attached to the bottom of the solar absorber film. The entire structure was supported by a foam with a suitable size and then placed at the air–water interface. The temperature evolution was recorded at regular time intervals. Infrared imaging was performed to directly monitor the temperature change of the solar absorber film under simulated solar light illumination.

*Statistical Analysis*: All statistical data were raw data without pre-processing. The size distributions of the photocatalysts were presented as mean ± standard deviation (SD) and acquired from 100 randomly selected nanospheres by the software named Nano Measurer 1.2. The photocatalytic properties were obtained from at least three independent experiments, and the variables were presented as mean ± SD. The data were analyzed using a one-way ANOVA analysis of variance and the Bonferroni’s multiple comparison test by GraphPad Prism9.5. Statistical significance was set at *p* < 0.05. Asterisks indicate significance on the graphs. The symbols *, **, ***, and **** represent *p* < 0.05, *p* < 0.01, *p* < 0.001, and *p* < 0.0001, respectively.

**Supplementary Note 1**

*Calculation of ∆G*_ammonia_ *in the work*:^[8]^ The overall reaction for photocatalytic nitrogen fixation with H_2_O_2_ as the product is

$$\frac{1}{2}N_{2}\left( g \right)+3H_{2}O\left( l \right)\to NH_{3}\left( g \right)+\frac{3}{2}{H_{2}O}_{2}(l)$$

The standard Gibbs free energy change ($\Delta G^{\circ}$) for the reaction is

$$\Delta G^{^{\circ}}=\sum\Delta G_{f}^{^{\circ}}(\mathrm{products})-\sum\Delta G_{f}^{^{\circ}}(\mathrm{reactants})$$

The standard Gibbs free energies of formation ($\Delta G_{f}^{^{\circ}}$, in kJ mol^−1^) are

$N_{2}\left( g \right)$: 0

$H_{2}O\left( l \right)$: −237.2

$NH_{3}\left( g \right)$: −16.45

${H_{2}O}_{2}(l)$: −120.4

Therefore, $\Delta G^{\circ}$ for the formation of 1 mole of $NH_{3}\left( g \right)$ can be calculated as

$$\Delta G^{^{\circ}}=\sum\Delta G_{f}^{^{\circ}}(\mathrm{products})-\sum\Delta G_{f}^{^{\circ}}\left( \mathrm{reactants} \right)=\left[ 1\times\Delta G_{f}^{^{\circ}}\left( NH_{3} \right)+\frac{3}{2}\times\Delta G_{f}^{^{\circ}}\left( {H_{2}O}_{2} \right) \right]-\left[ \frac{1}{2}\times\Delta G_{f}^{^{\circ}}\left( N_{2} \right)+3\times\Delta G_{f}^{^{\circ}}\left( H_{2}O \right) \right]=\left( -16.45 \right)+[\frac{3}{2}\times\left( -120.4 \right)]-\left[ 3\times\left( -237.2 \right) \right]=514.5 kJ \mathrm{mol}^{-1}$$

**Supplementary Note 2**

*Comparison of SCCEs*: In the work,^[8]^ an AM 1.5G lamp was used as the light source. The light intensity was adjusted to 1000 W m^−2^. The irradiation area was 7.85 × 10^−3^ m^−2^. The temperature of the system was 25 °C. The N_2_ flow rate was 300 mL min^−1^. The amount of the photocatalysts was 3 g. The volume of the solution was 700 mL. The photocatalyst concentration was 4.29 g L^−1^.

For more accurate comparison, the experimental conditions in our own work is also outlined here. A 300 W xenon lamp with an AM 1.5G filter was used as the light source. The light intensity was adjusted to 1000 W m^−2^. The irradiation area was 4.072 × 10^−3^ m^−2^. The temperature of the system was 25 °C. The N_2_ flow rate was 10 sccm (10 mL min^−1^). The amount of the photocatalyst varied from 20 to 240 mg. The volume of the solution was 80 mL. The photocatalyst concentration was correspondingly varied from 0.25 to 3 g L^−1^.

The major differences lie in the N_2_ flow rate and photocatalyst concentration. As demonstrated in Figure S19, the N_2_ flow rate had been proven to have limited influence on the photocatalytic activity. Figure 2a and Figure S18 show that the SCCE values gradually increase and get saturated with increasing photocatalyst concentrations. As a result, the SCCE value at a 4.29 g L^−1^ photocatalyst concentration should be no smaller than that at 3 g L^−1^ (≥ 0.41%), which is 1.4 times larger than the value reported in the work.^[8]^

**Supplementary Note 3**

*Evaluation of the solar absorber films in terms of the scalability and technoeconomic implications*: The MoO_3–_*_x_*/10% Ag samples were prepared by an aerosol-spray process. This method has a clear preparation principle and an uncomplicated preparation process. Through the increase of the size of the tube furnace and adjustment of the spray efficiency and carrier gas flow rate, the preparation can be readily upgraded. However, the relatively high synthesis temperature (350 ℃) is no doubt a burden on the electricity. 1 g of the precursor can yield ~100 mg of the nanospheres. The conversion ratio is ~10%, which is primarily determined by the inefficient cross-linking and the inevitable deposition on the walls of the quartz tube. At the same time, the relatively high cost of the Ag precursor (CH_3_COOAg, ~2 USD g^−1^) will also significantly impact the overall cost of the photocatalysts. Silver acetate was selected owing to its superior solubility and lower decomposition temperature compared to alternative Ag precursors. Therefore, the scalability of the photocatalysts is feasible, but the cost reduction is a key challenge.

Compared to the expensive photocatalysts, the fabrication of the polymer films is of low cost. The raw materials are readily available and the preparation method is facile. The freeze-drying method is relatively straightforward and can be easily replicated on a large scale. We also note that the process of freeze-drying is time-consuming (~2 days per batch), which is the main issue regarding the technoeconomic implications.

The solar absorber films are biocompatible, non-toxic, and chemically stable, which facilitate recycling and end-of-life disposal. The solar absorber films with a high SCCE (0.28%) can yield ~148 μmol NH_3_ in 9 h, which demonstrates good productivity and energy utilization ability.

In all, our photocatalytic system shows great promise for scalability and technoeconomic implications in terms of the relatively simple synthesis method and the great photocatalytic performance. However, the cost of the precursor, the time-consuming process, and the photocatalyst deactivation are key challenges that need to be addressed for large-scale implications.


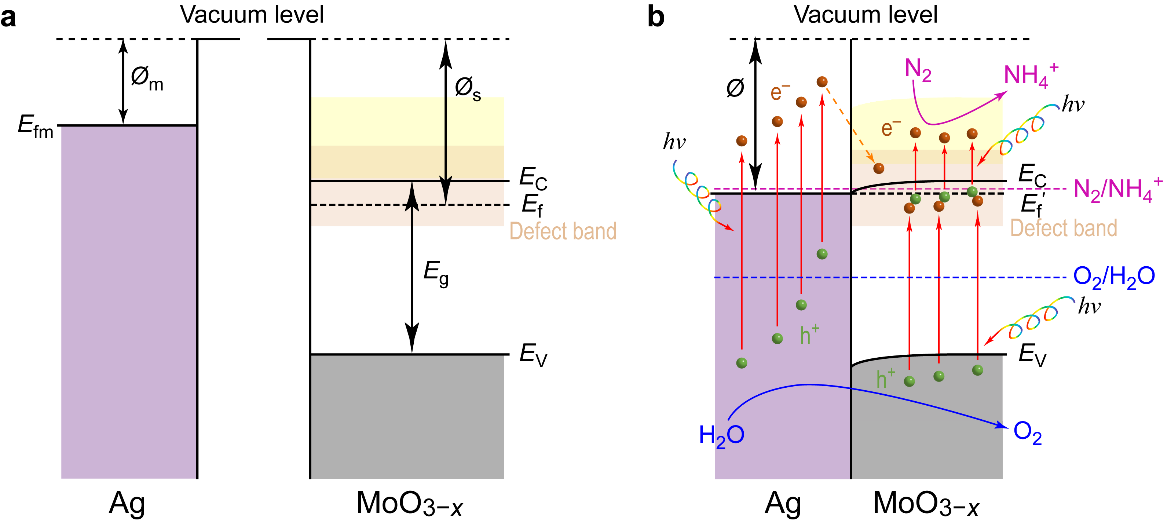


**Figure** **S1.** Band alignment. a) Band alignment of plasmonic Ag and MoO_3–_*_x_* without contact. Abundant OVs in MoO_3–_*_x_* lead to the formation of a defect band, which overlaps with the conduction band. b) Band alignment of plasmonic Ag and MoO_3–_*_x_* in contact. The plasmonic generation of hot charge carriers, their subsequent transfer and redox reactions are also illustrated.


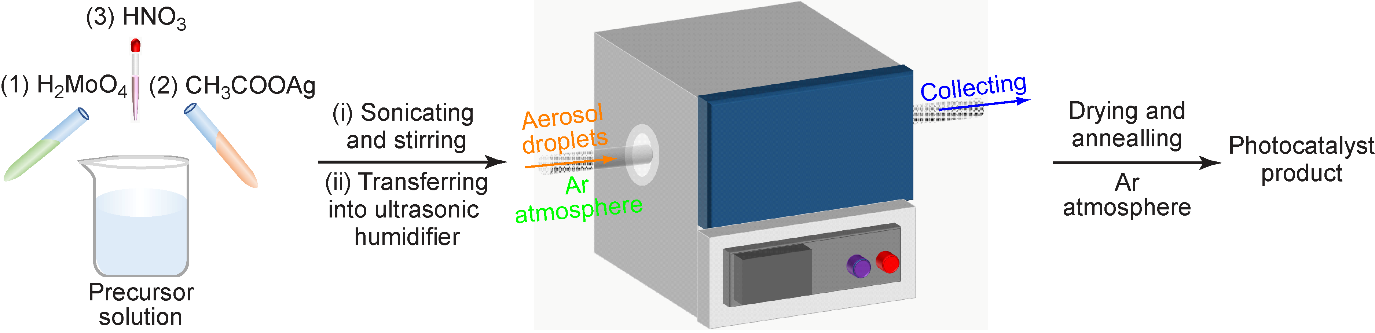


**Figure** **S2.** Schematic illustrating the synthesis process.


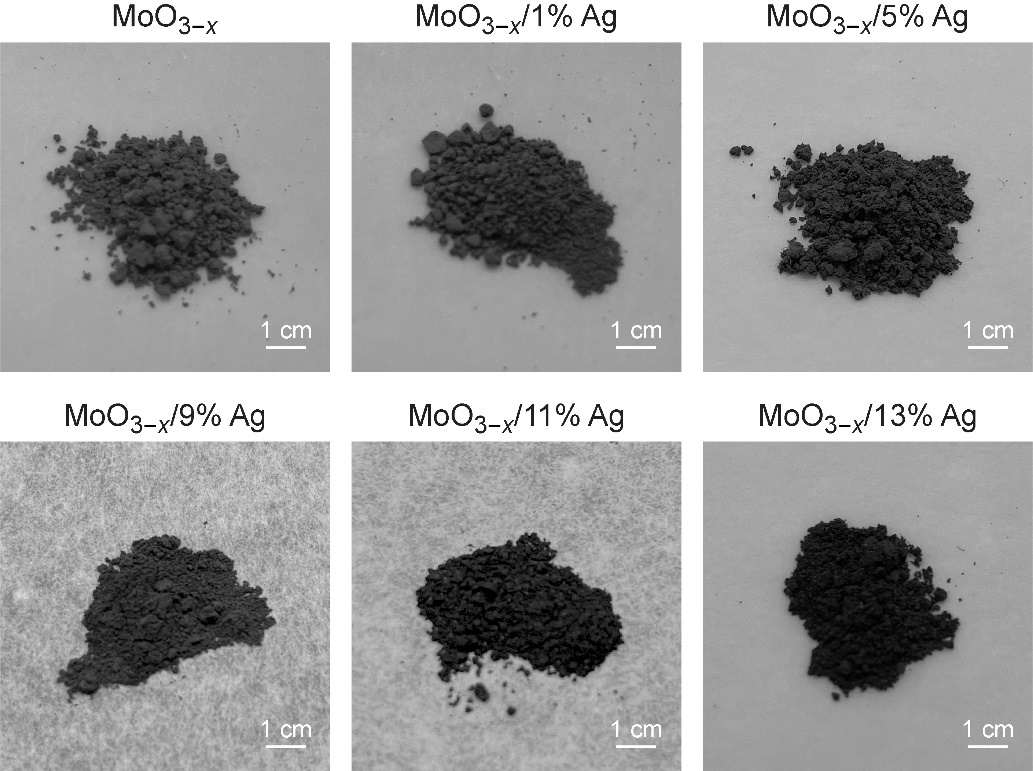


**Figure** **S3.** Photographs of the different MoO_3–_*_x_*/Ag samples.


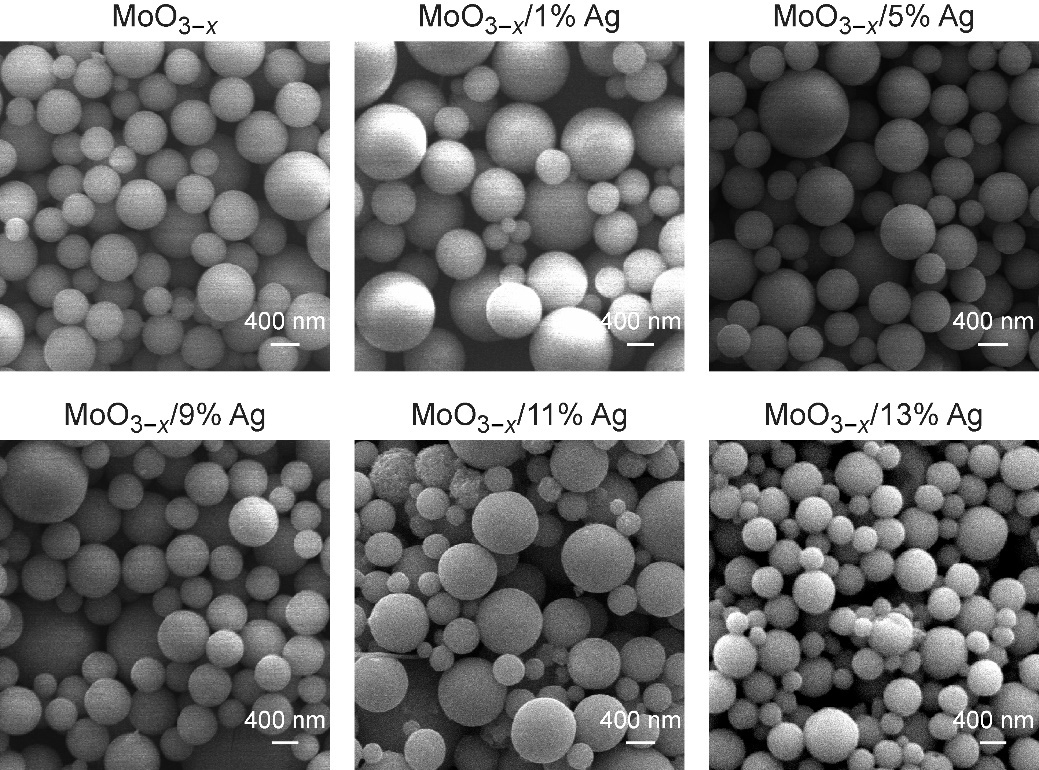


**Figure** **S4.** SEM images of the different MoO_3–_*_x_*/Ag samples.


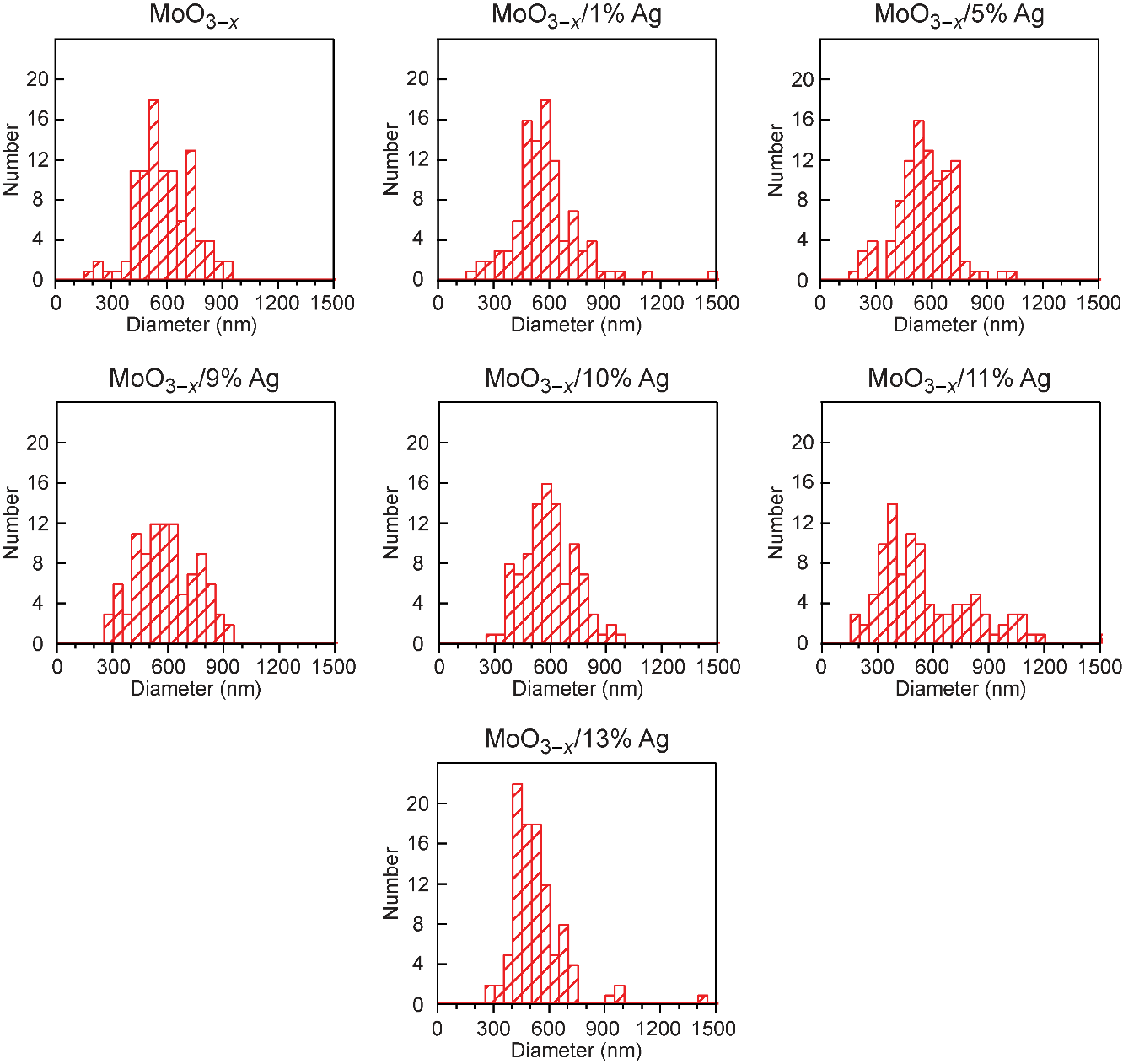


**Figure** **S5.** Size distributions of the different MoO_3–_*_x_*/Ag samples. The size distributions were measured from the SEM images. The average diameters are 579 ± 149, 574 ± 181, 559 ± 157, 584 ± 161, 589 ± 141, 561 ± 263, 531 ± 152 nm, respectively.


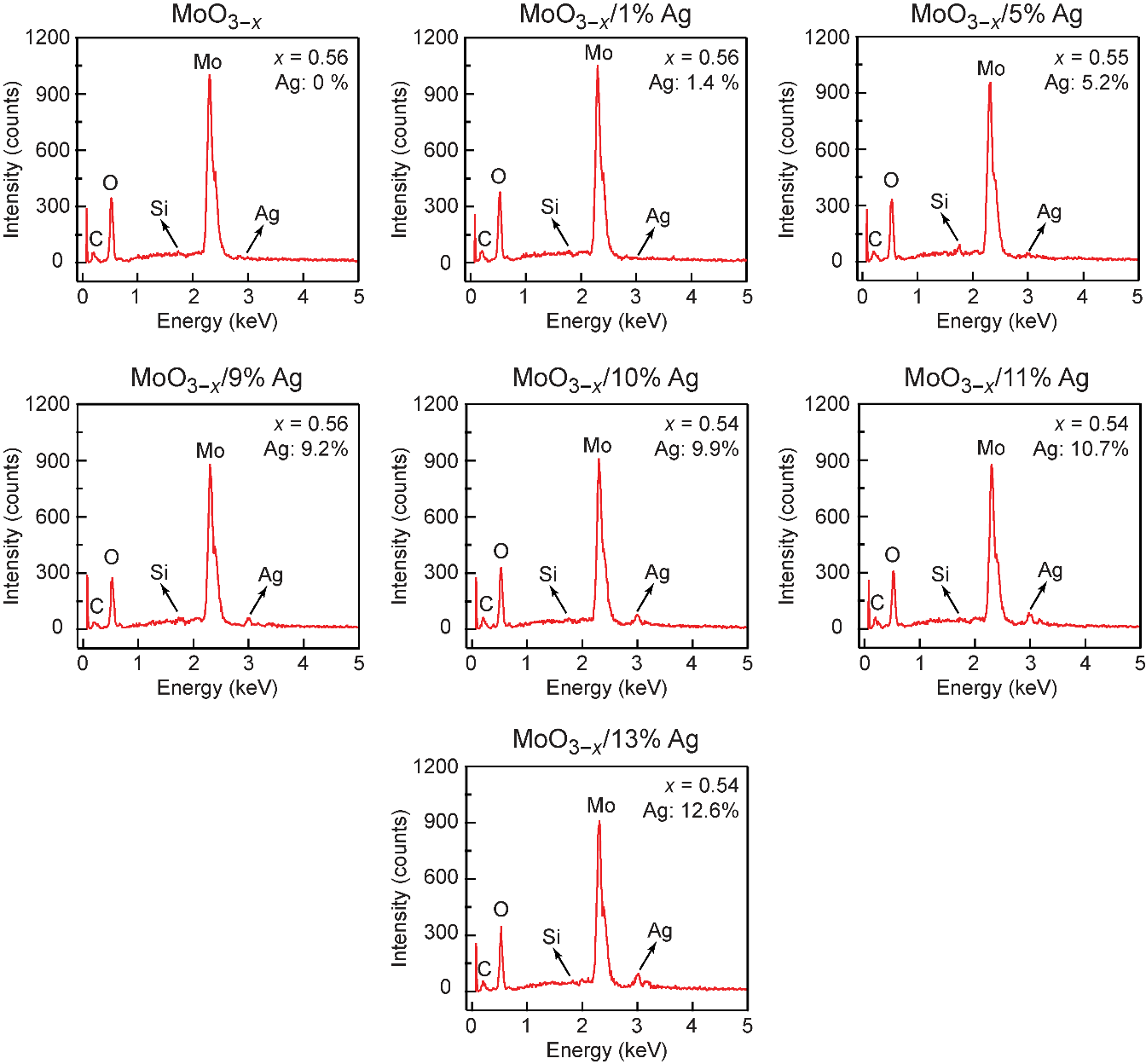


**Figure** **S6.** EDX spectra of the different MoO_3–x_/Ag samples. The EDX spectra were measured during SEM imaging. The measured Ag molar percentages and *x* values in the samples are given in the plots.


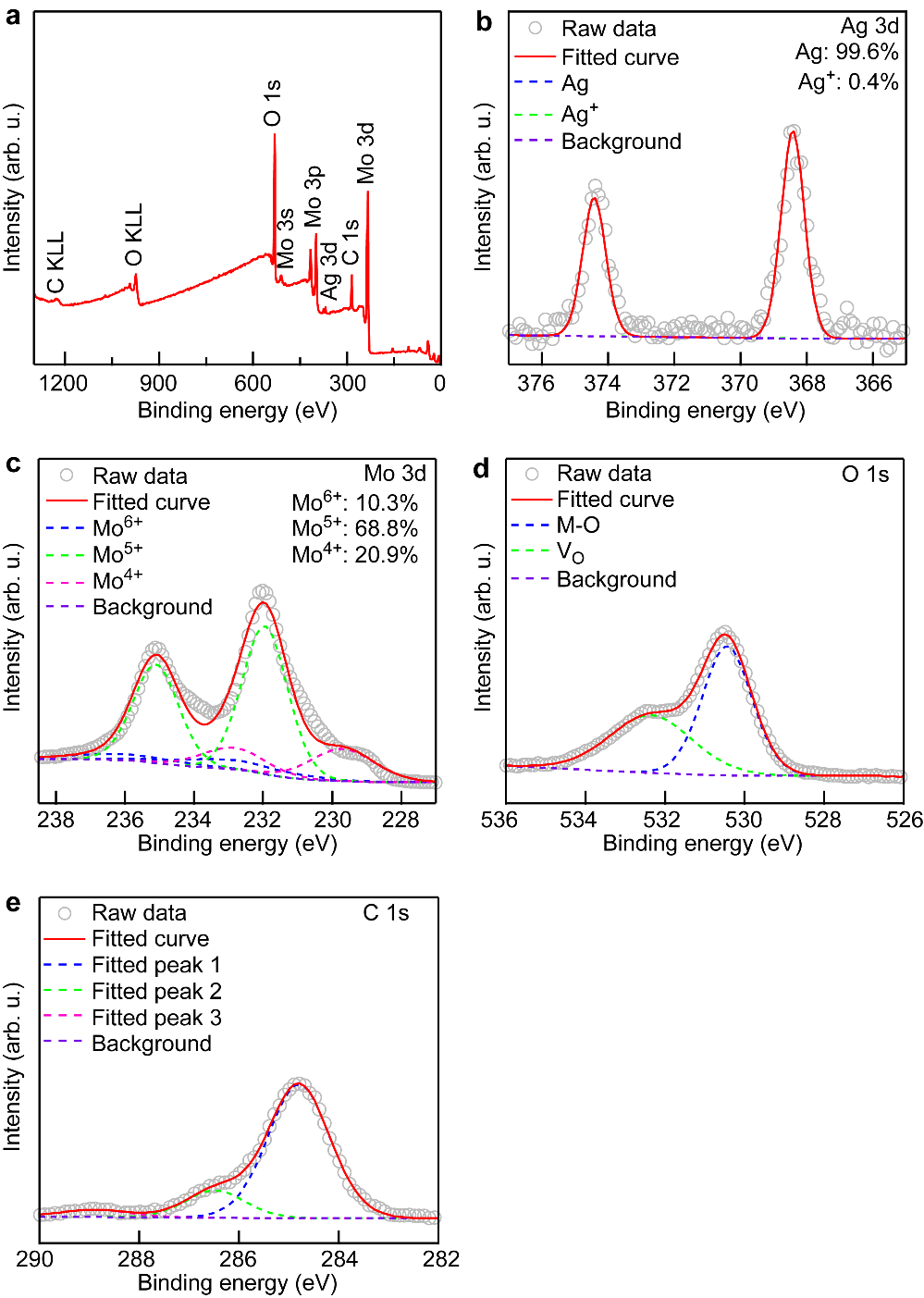


**Figure** **S7.** XPS spectra of the MoO_3–_*_x_*/10% Ag sample. a) Survey XPS spectrum. b–e) High-resolution XPS spectra of Ag 3d (b), Mo 3d (c), O 1s (d), and C 1s (e). The blue dashed lines in (b) overlap with the red lines. The valence state of Mo was found from XPS to be 4.89.


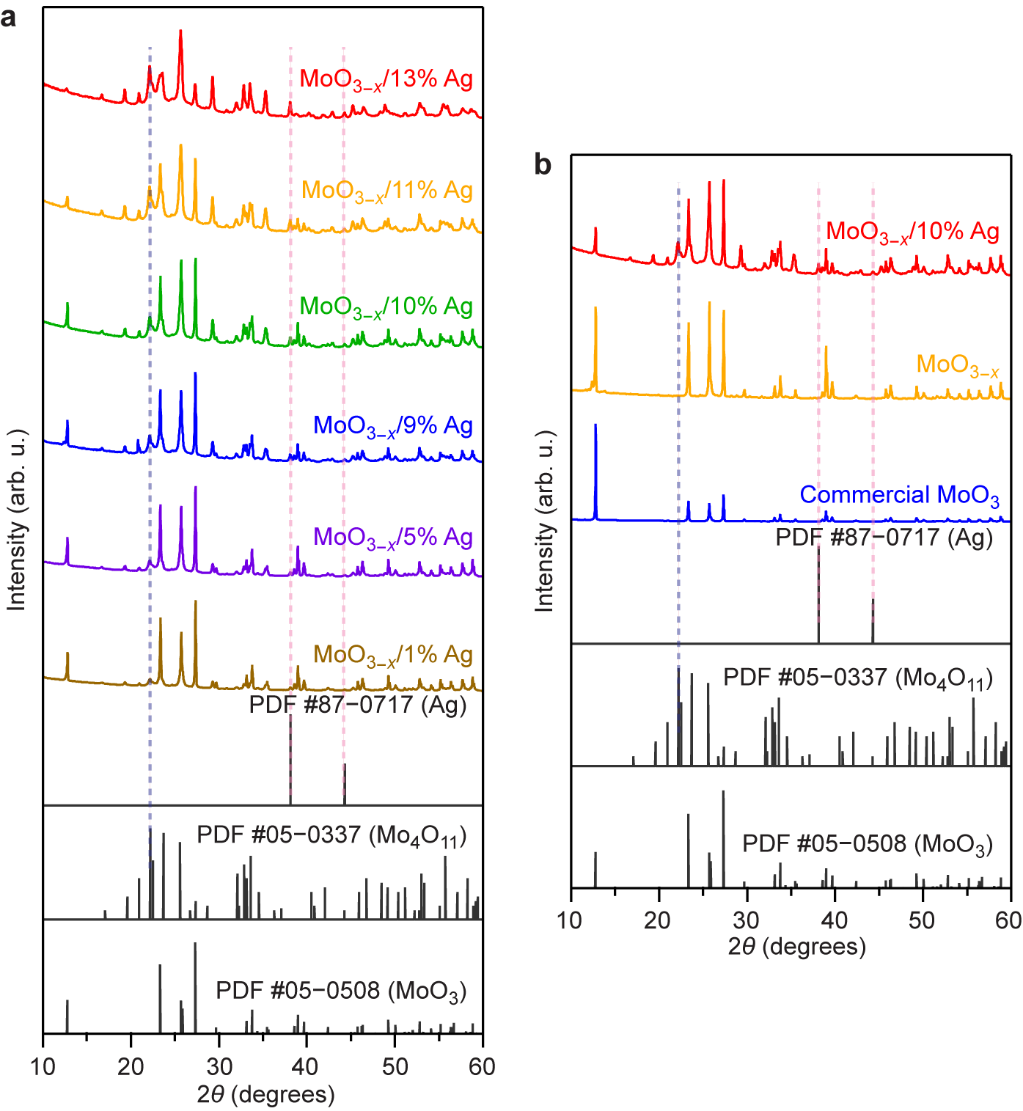


**Figure** **S8.** XRD patterns of the different MoO_3–_*_x_*/Ag samples. a) XRD patterns of the MoO_3–_*_x_*/Ag samples with 1, 5, 9, 10, 11, and 13% Ag. The standard powder diffraction patterns (JCPDS No. 05–0508 for orthorhombic α-MoO_3_, JCPDS No. 05–0337 for the Magneli Mo_4_O_11_ phase, and JCPDS No. 87–0717 for face-centered cubic Ag) are also displayed as references. b) XRD patterns of commercial intrinsic MoO_3_ nanoparticles, the MoO_3–_*_x_* and MoO_3–_*_x_*/10% Ag samples. The standard powder diffraction patterns (JCPDS No. 05–0508 for orthorhombic α-MoO_3_ and JCPDS No. 05–0337 for the Magneli Mo_4_O_11_ phase) are also displayed for references.


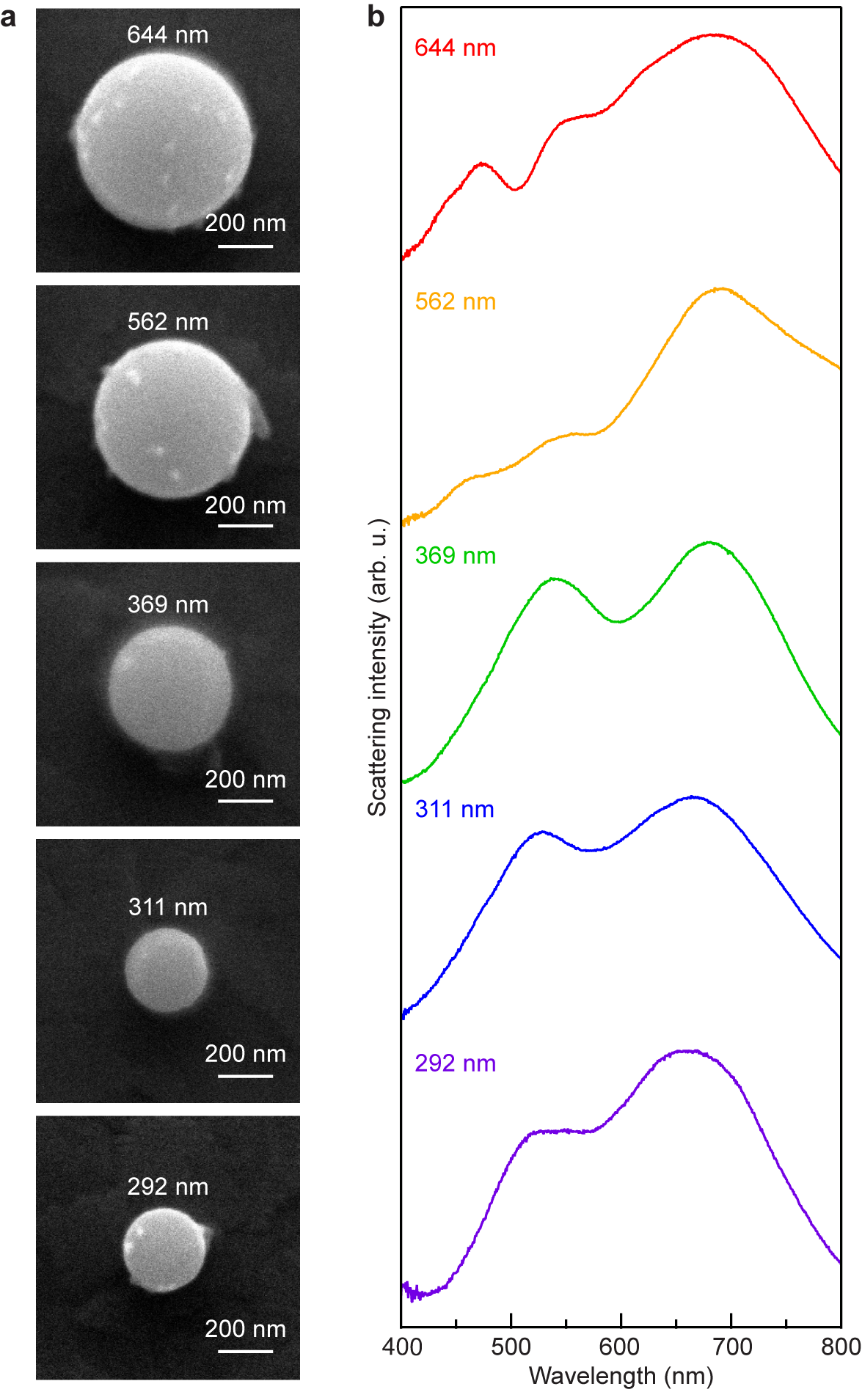


**Figure** **S9.** Single-particle dark-field scattering spectra of the MoO_3–_*_x_*/10% Ag nanospheres. a) SEM images of 5 representative MoO_3–_*_x_*/10% Ag nanospheres with different diameters. b) Measured scattering spectra of the nanospheres shown in (a). The scattering intensity of the Ag nanoparticles is too weak to be observed because of the small nanoparticle sizes. Most incident light is absorbed rather than scattered on the small Ag nanoparticles.


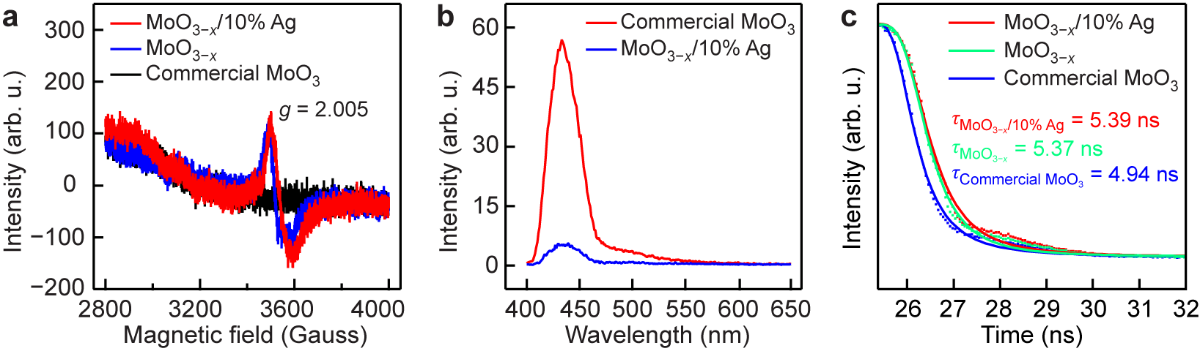


**Figure** **S10.** OV characterization. a) EPR spectra of commercial intrinsic MoO_3_ nanoparticles, the MoO_3–_*_x_* and MoO_3–_*_x_*/10% Ag samples at the ambient temperature and pressure. b) Steady-state PL spectra of commercial intrinsic MoO_3_ nanoparticles and the MoO_3–_*_x_*/10% Ag nanospheres. The excitation wavelength was 370 nm. c) Time-resolved fluorescence decay spectra. The excitation wavelength was 366 nm. The observation wavelength was 435 nm.


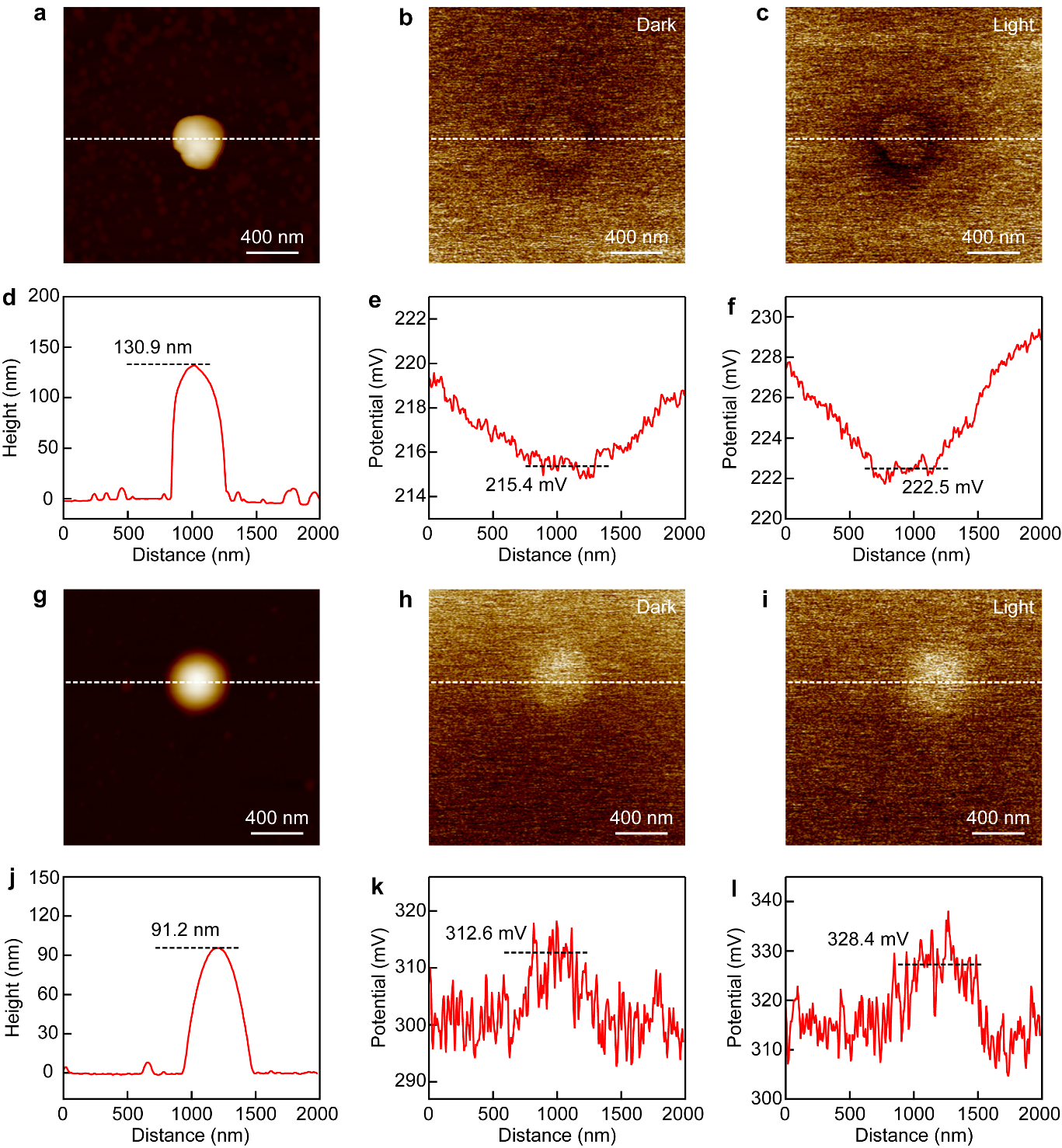


**Figure** **S11.** KPFM measurements. a) Atomic force microscopy image of a MoO_3–_*_x_* nanosphere. b) Surface potential of the MoO_3–_*_x_* nanosphere in the dark. c) Surface potential of the MoO_3–_*_x_* nanosphere under light illumination. d) Height value of the MoO_3–_*_x_* nanosphere along the white dashed line. e) Surface potential of the MoO_3–_*_x_* sample in the dark along the white dashed line. f) Surface potential of the MoO_3–_*_x_* nanosphere under light illumination along the white dashed line. g) Atomic force microscopy image of a MoO_3–_*_x_*/10% Ag nanosphere. h) Surface potential of the MoO_3–_*_x_*/10% Ag nanosphere in the dark. i) Surface potential of the MoO_3–_*_x_*/10% Ag nanosphere under light illumination. j) Height value of the MoO_3–_*_x_*/10% Ag nanosphere along the white dashed line. k) Surface potential of the MoO_3–_*_x_*/10% Ag nanosphere in the dark along the white dashed line. l) Surface potential of the MoO_3–_*_x_*/10% Ag nanosphere under light illumination along the white dashed line.

According to Equation (4), the workfunctions of the pristine MoO_3–_*_x_* nanosphere were calculated to be 5.189 eV (dark) and 5.181 eV (light), respectively. The slight decrease in the workfunction (8 meV) under light illumination suggests a rise in the Fermi level of the MoO_3–_*_x_* sample. This Fermi level shift is primarily caused by electron excitation to the conduction band (CB) in the n-type semiconductor. The excited electrons populate the CB orbitals, resulting in a rise in the Fermi level. For the MoO_3–_*_x_*/10% Ag nanosphere, the workfunctions of the MoO_3–_*_x_* host were found to be 5.091 eV (dark) and 5.076 eV (light), respectively. The decrease in the workfunction under dark conditions upon Ag incorporation confirms electron transfer from Ag to MoO_3–_*_x_* after close contact, validating the Schottky-barrier-free nature of the MoO_3–_*_x_*/10% Ag sample. Under light illumination, the larger decrease (15 meV) in the workfunction for the hybrid sample than that (8 meV) for the pristine MoO_3–_*_x_* sample suggests additional hot electron injection from the plasmonic Ag nanoparticles into the CB of MoO_3–_*_x_*, further populating its CB orbitals.


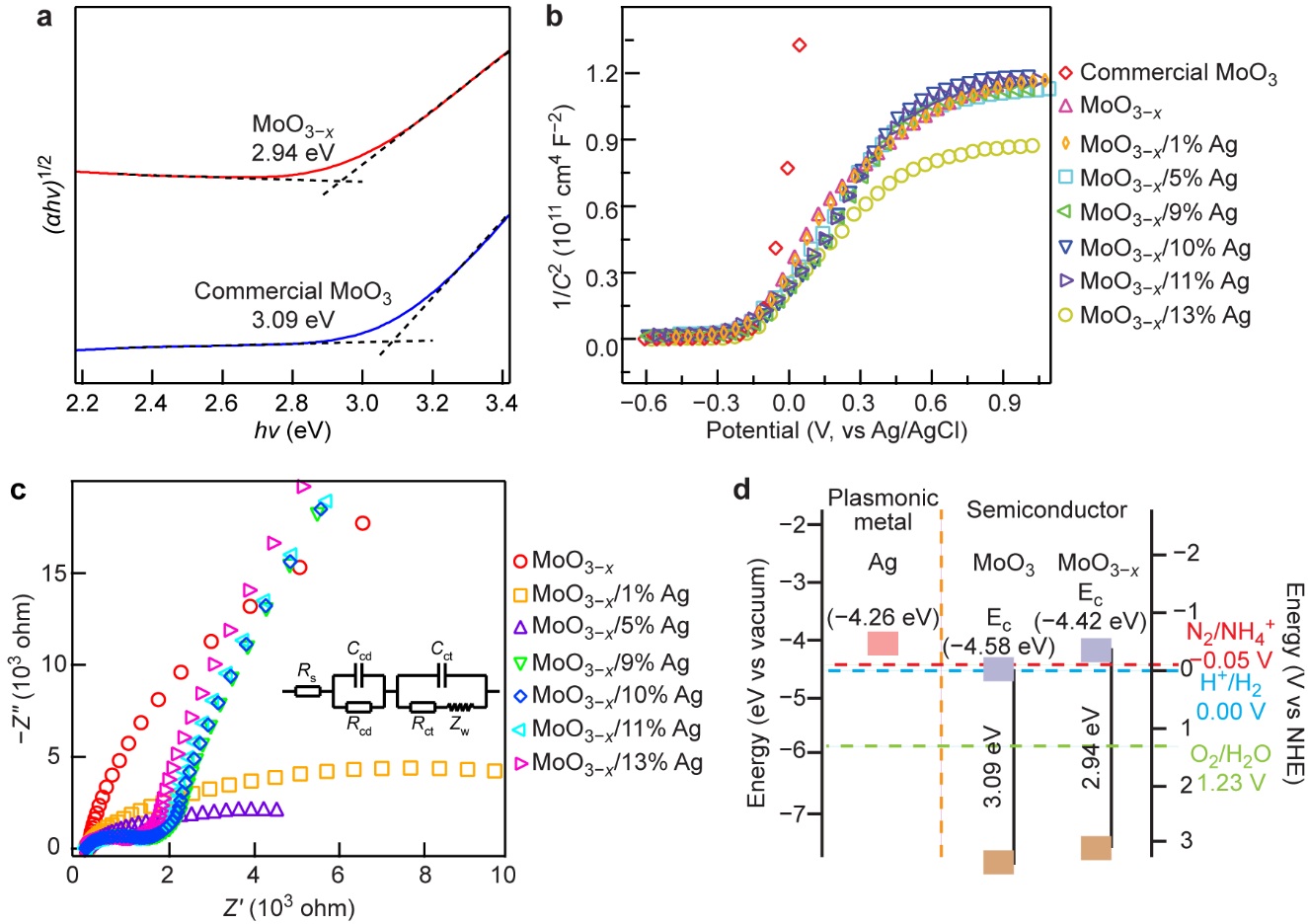


Figure S12. Band structures of the different MoO_3–_*_x_*/Ag samples. a) Tauc plots and determined energy bandgaps for commercial intrinsic MoO_3_ nanoparticles and the MoO_3–_*_x_* nanospheres. b) Mott–Schottky plots of commercial intrinsic MoO_3_ nanoparticles and the MoO_3–_*_x_*/Ag samples with 0, 1, 5, 9, 10, 11 and 13% Ag. c) Impedances of the MoO_3–_*_x_*/Ag samples with 0, 1, 5, 9, 10, 11 and 13% Ag. The inset shows the equivalent circuit used to fit the impedance data. *R*_s_ is the series resistance, which includes the material resistance and contact resistance of the test system, *R*_cd_ is the charge-transfer resistance at the nanosphere/electrode interface, *R*_ct_ is the charge-transfer resistance at the nanosphere/electrolyte interface, *C*_cd_ is the capacitance of the nanosphere/electrode interface, i.e., the internal capacitance of the material, *C*_ct_ is the capacitance of the nanosphere/electrolyte interface, and *Z*_w_ is the Warburg impedance, i.e., the mass transfer resistance. d) Electronic band energy levels of plasmonic Ag and MoO_3–_*_x_* synthesized in this work.


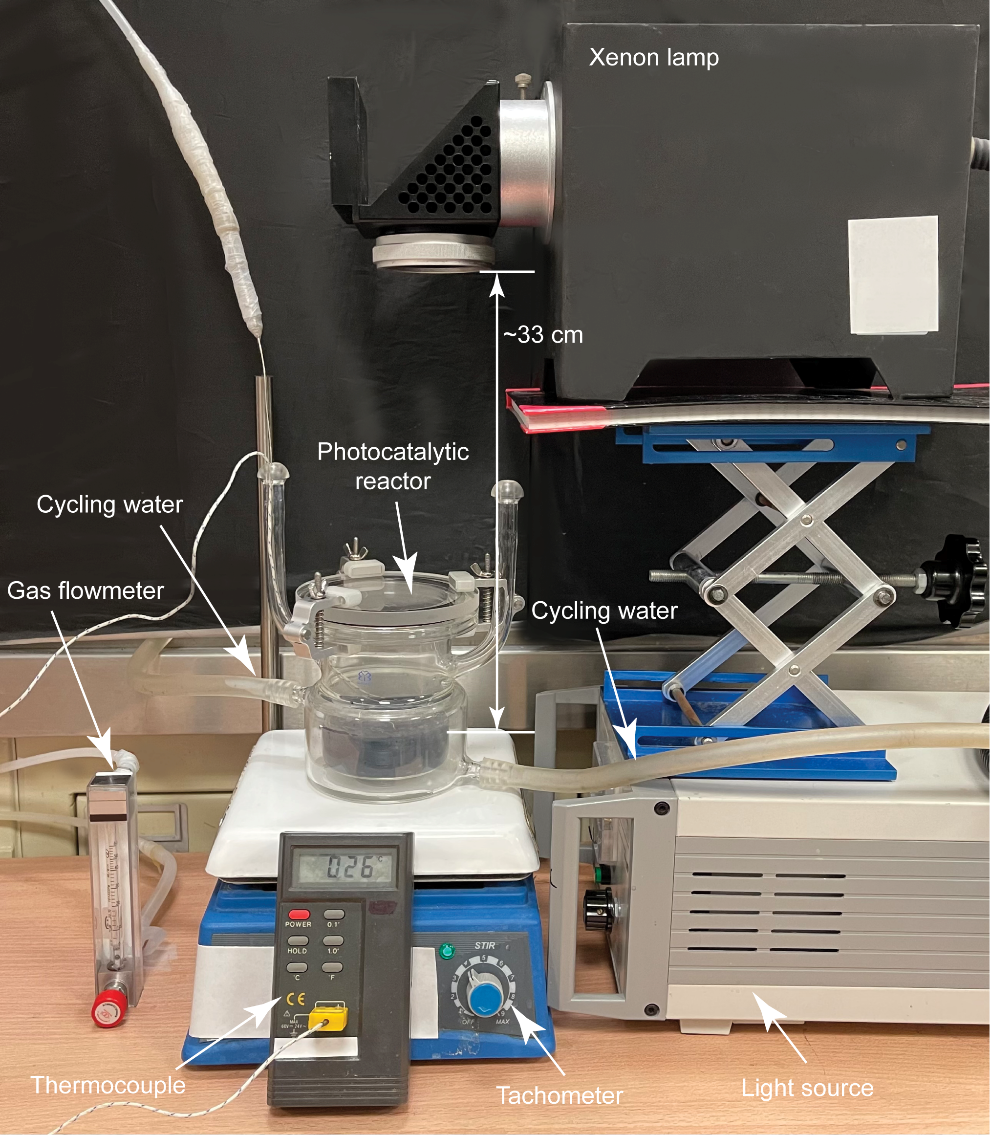


**Figure** **S13.** Photograph of the experimental setup for the PCNF tests. The distance between the xenon lamp and the water surface was ~33 cm for AM 1.5 sunlight irradiation.


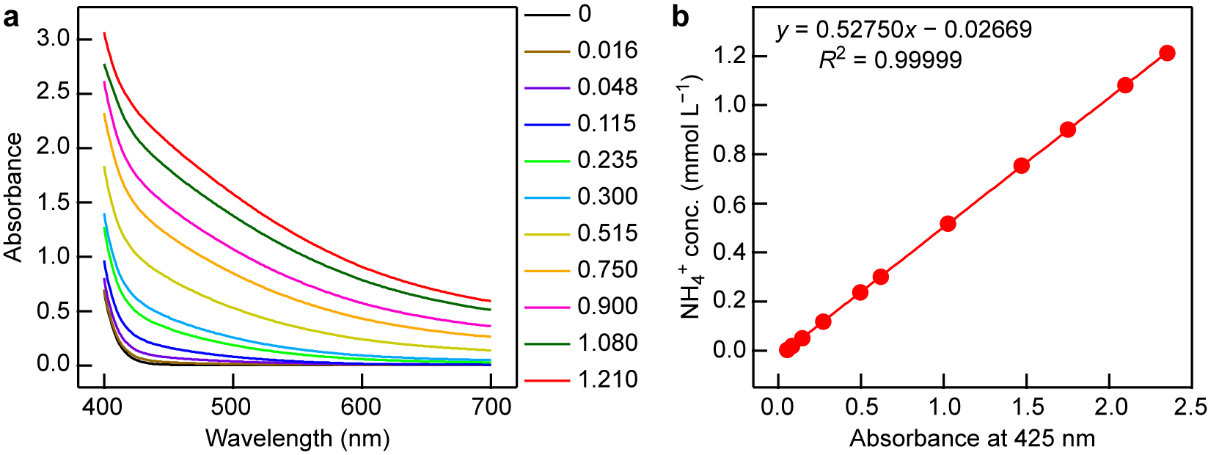


**Figure** **S14.** Dependence of the absorbance on the NH_4_^+^ determination. a) Absorption spectra of the standard NH_4_^+^ solutions at different concentrations. b) Linear relationship between the absorbance at 425 nm and the NH_4_^+^ concentration.


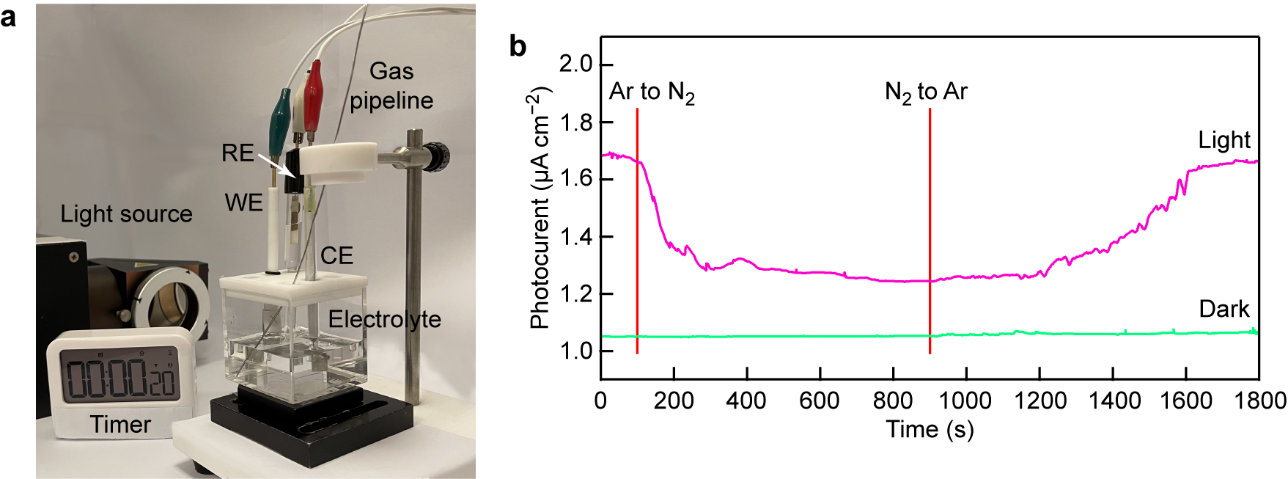


Figure S15. Photocurrent measurements. a) Photograph of the setup for the photocurrent measurements under different conditions. b) Photocurrent response of the MoO_3–_*_x_*/10% Ag sample when the bubbling gas was suddenly switched between N_2_ and Ar in both light and dark.


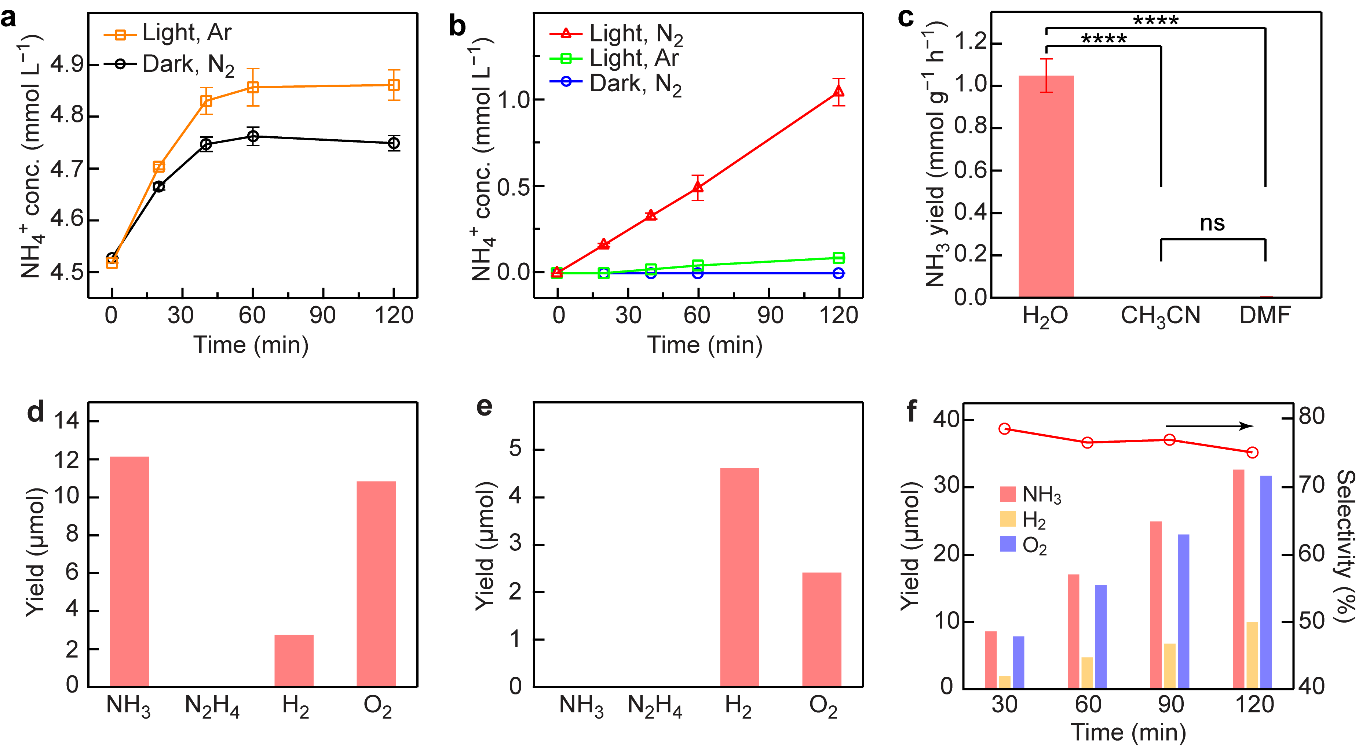


Figure S16. Control experiments for PCNF. a) Time-dependent production of NH_3_ for the MoO_3–_*_x_*/10% Ag sample under different conditions without pre-treatment. b) Time-dependent production of NH_3_ for the MoO_3–_*_x_*/10% Ag sample under different conditions after 40 min pre-treatment. The error bars on the data points for the NH_4_^+^ concentrations obtained from the reactions without either light illumination or N_2_ gas bubbling are too small to be seen. c) NH_3_ yields of the MoO_3–_*_x_*/10% Ag sample in the different solvents under light illumination. The error bars on the data points for the NH_3_ yields obtained from the reactions with either CH_3_CN or DMF as the solvent are too small to be seen. **** represents *p* < 0.0001, and “ns” means not significant. d) Photocatalytic product amounts of NH_3_, N_2_H_4_, H_2_, and O_2_ in a sealed reactor with the MoO_3–_*_x_*/10% Ag sample and N_2_ under solar light illumination for 0.5 h. e) Photocatalytic product amounts of NH_3_, N_2_H_4_, H_2_, and O_2_ in a sealed reactor with the MoO_3–_*_x_*/10% Ag sample and Ar under solar light illumination for 0.5 h. f) Photocatalytic product amounts (left axis, bars) and time-dependent selectivities of NH_3_ (right axis, empty circles) for the MoO_3–_*_x_*/10% Ag sample under solar light illumination.


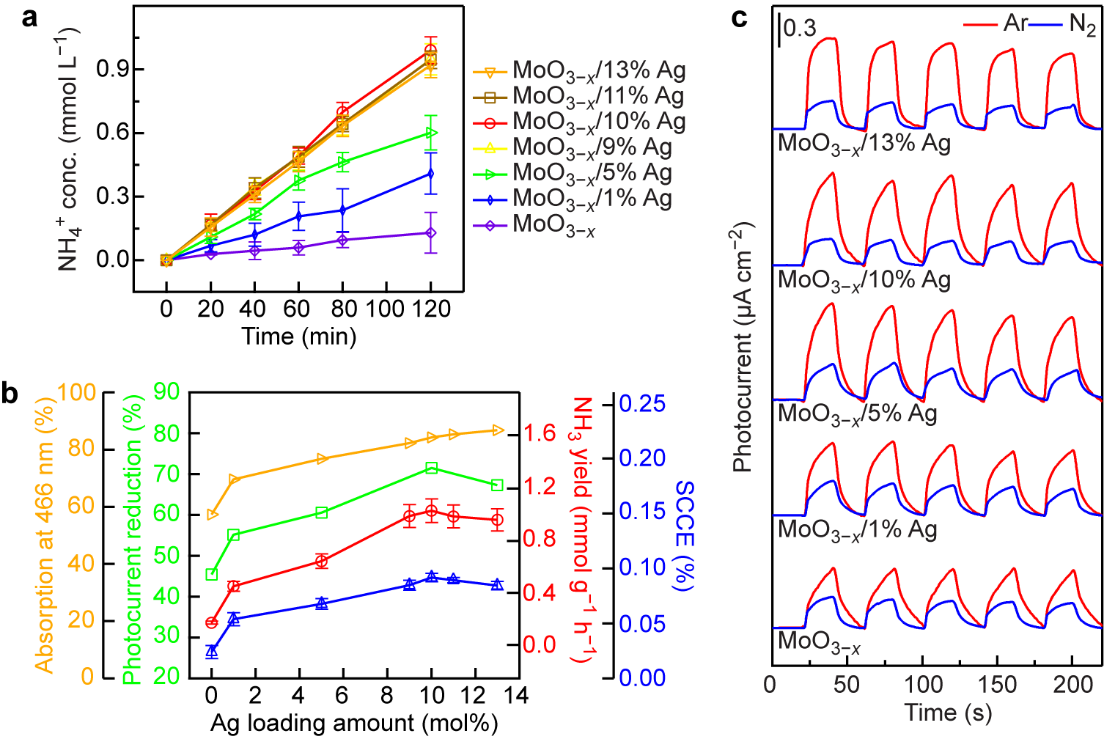


Figure S17. Photocatalytic performance and photocurrent responses of the MoO_3–_*_x_*/Ag samples. a) Time-dependent production of NH_3_ for the MoO_3–_*_x_*/Ag samples with 0, 1, 5, 9, 10, 11 and 13% Ag. b) Effect of the Ag loading amount on the light absorption at 466 nm (the first left axis), the photocurrent reduction (the second left axis), the NH_3_ yield (the first right axis), and the SCCE (the second right axis) for the MoO_3–_*_x_*/Ag samples. c) Photocurrent responses of the MoO_3–_*_x_*/Ag samples with 0, 1, 5, 9, 10, 11 and 13% Ag under identical light illumination conditions. The measurements were performed in both Ar and N_2_ atmospheres. The light illumination was switched on and off repeatedly.

The photocurrent intensities were greatly enhanced in both N_2_ and Ar atmosphere when the light was turned on, indicating that a large number of electrons were generated upon light illumination. The shark-fin-like shape of the photocurrent signal was caused by the trapping effect of OVs, where the generation and diminution of the photocurrent signal were delayed.^[9]^ Compared to that under Ar bubbling, the reduced photocurrent intensity of each photocatalyst under N_2_ bubbling suggests electron consumption by PCNF.


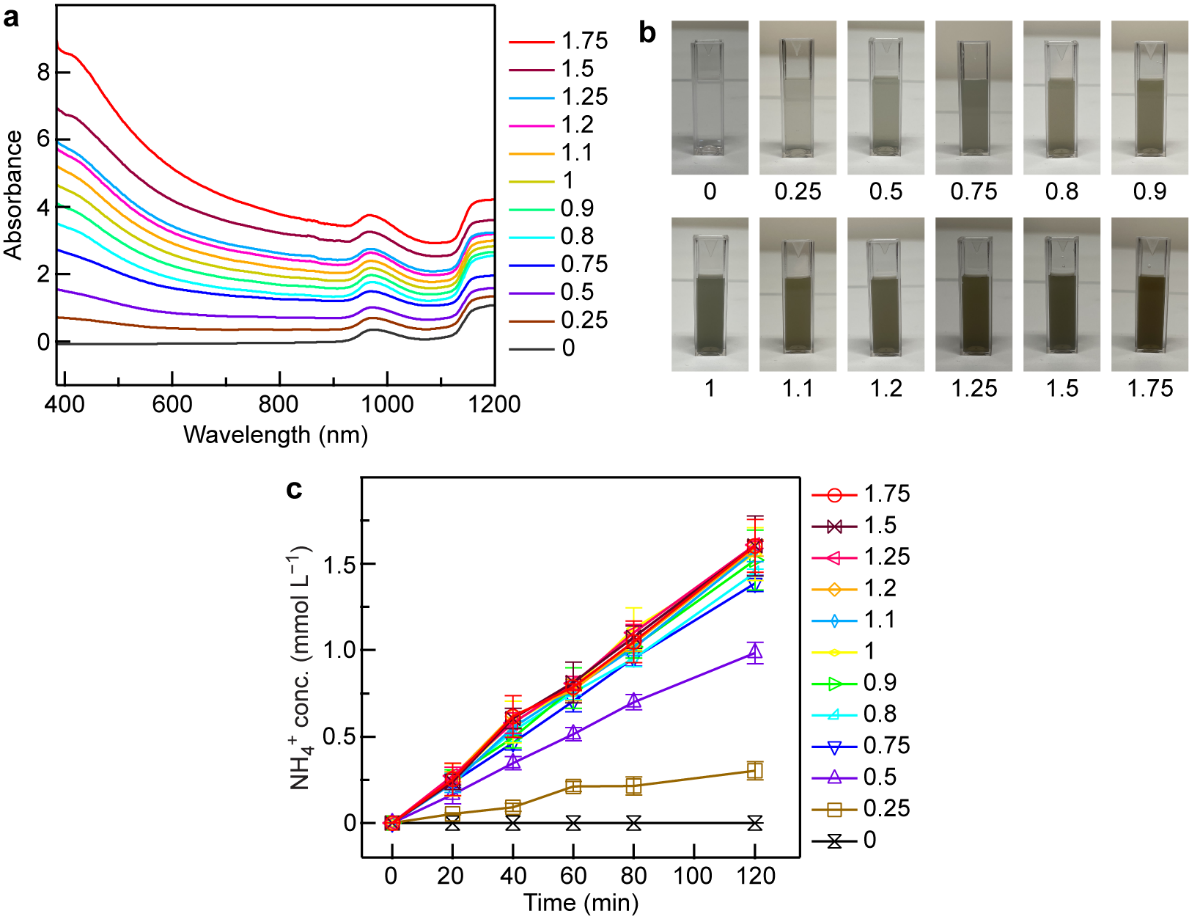


Figure S18. Light absorption spectra and photocatalyitc activities of the aqueous photocatalyst solutions. a) Light absorbance spectra of the MoO_3–_*_x_*/10% Ag sample with different mass concentrations in water. b) Photographs of the MoO_3–_*_x_*/10% Ag sample with different mass concentrations in water. The unit of the concentration is g L^−1^. c) Time-dependent production of NH_3_ at different photocatalyst concentrations. The used photocatalyst was MoO_3–_*_x_*/10% Ag. The unit of the concentration is g L^−1^.


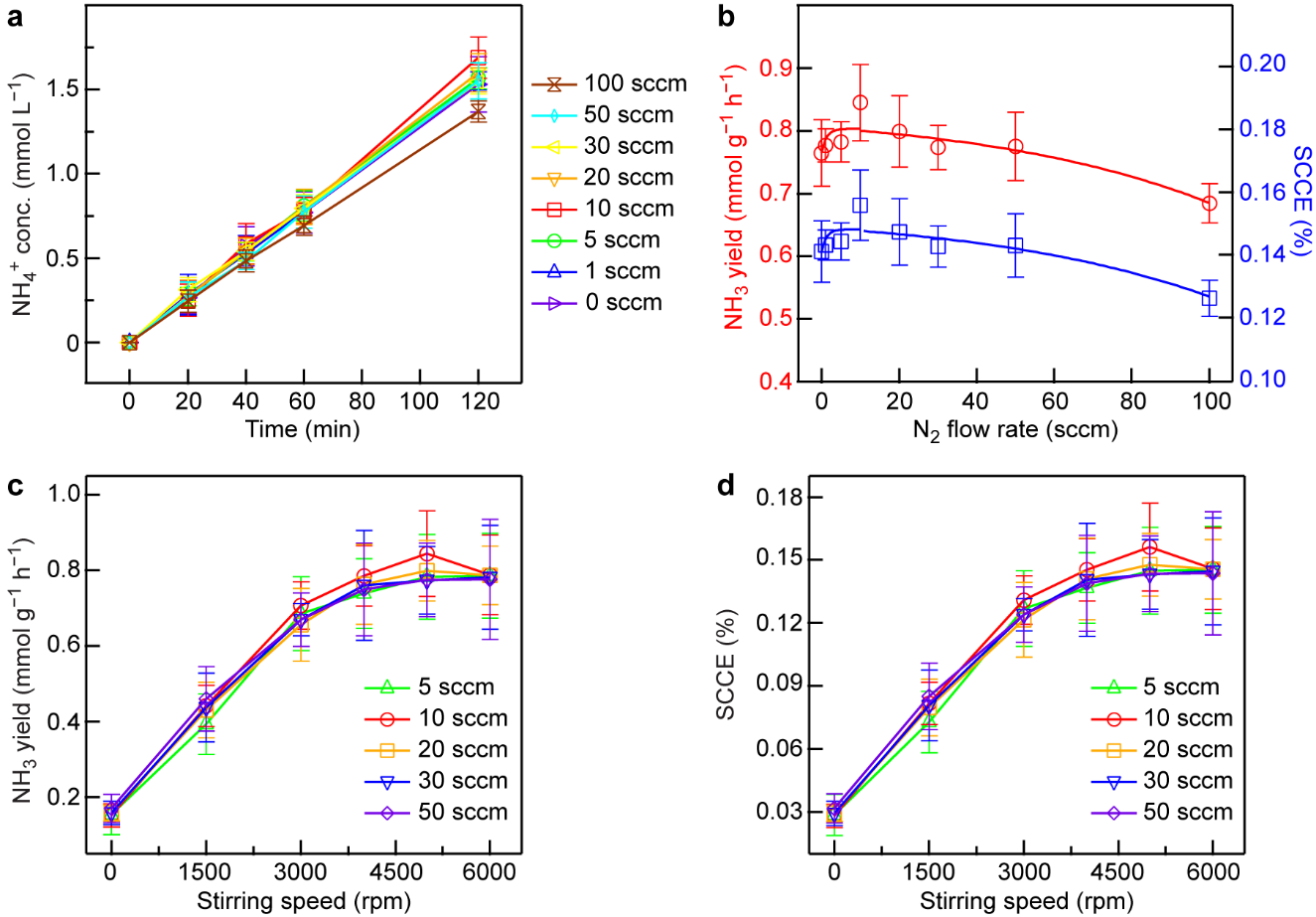


Figure S19. Photocatalytic activities of the MoO_3–_*_x_*/10% Ag sample under various N_2_ flow rates and stirring speeds. a) Time-dependent production of NH_3_ at different N_2_ flow rates. The photocatalyst was the MoO_3–_*_x_*/10% Ag sample. Its concentration was fixed at 1 g L^−1^. b) Effect of the N_2_ flow rate on the NH_3_ yield (left axis) and the SCCE (right axis) for the MoO_3–_*_x_*/10% Ag sample with 1 g L^−1^ photocatalyst concentration. c) Effect of the stirring speed (rpm: revolutions per minute) and N_2_ flow rate on the NH_3_ yield for the MoO_3–_*_x_*/10% Ag sample at 1 g L^−1^ concentration. d) Effect of the stirring speed and N_2_ flow rate on the SCCE for the MoO_3–_*_x_*/10% Ag sample at 1 g L^−1^ concentration.

As mentioned in the main text, N_2_ pre-bubbling and stirring were employed to obtain a N_2_-saturated suspension because of the sluggish N_2_ diffusion kinetics. The specific effect of this dull dynamics during PCNF was therefore investigated by controlling the kinetics-associated N_2_ flow rate and stirring speed.

The stirring speed was first fixed at 5,000 rpm, and the MoO_3–_*_x_*/10% Ag sample at 1 g L^−1^ concentration gave similar NH_3_ production rates at various N_2_ flow rates (Figure S19a,b).

To determine if the PCNF reaction was reaction- or diffusion-controlled when the solution was saturated with N_2_ before light illumination, the diffusion kinetics were analyzed.

Take the N_2_ flow rate of 10 sccm as an example. The reaction rate of N_2_ can be estimated from the PCNF experiment to be *r*_total_ = 9.4 × 10^−3^ μmol s^−1^. As the reaction was conducted in an aqueous solution, H_2_O should be in excess and we assume that N_2_ is diffusion-limited. When the solution was saturated with N_2_, the concentration of N_2_ can be estimated to be 6.8 × 10^−4^ mol L^−1^, which is the maximal solubility of N_2_ at 25 ℃ and atmospheric pressure.

The transition from convection to diffusion is gradual upon approaching the photocatalyst surface, but it is convenient to model this situation as two distinct regions: a convectively supplied reservoir of N_2_, and a diffusive boundary layer between this reservoir and the photocatalyst surface. The reactant concentration *n*_A_ just over the surface is always lower than that in the reservoir *n*_∞_. This concentration gradient drives the reactant diffusion flux *J*_A_. The edge of the concentration boundary layer in which this diffusion occurs, *δ*_n_, is arbitrarily but usually defined as the plane at which the reactant concentration drops by 1% from *n*_∞_. We therefore have *n*_∞_ = 6.8 × 10^−4^ mol L^−1^ and 99%*n*_∞_ = 6.732 × 10^−4^ mol L^−1^.

The diffusion flux toward the surface of an individual photocatalyst nanosphere *J*_A_ can be calculated from the following formula

$J_{A}=-D\frac{{99\%n}_{\infty}-n_{A}}{\delta_{n}}$ (16)

where $D$ is the diffusion coefficient at the atmospheric pressure and a given temperature. In our work, the value of $D$ for N_2_ in water at 25 ℃ and atmospheric pressure was 2.00 × 10^−5^ cm^2^ s^−1^.

If the reaction is diffusion-controlled, all diffused N_2_ molecules would be consumed at the surface of the photocatalyst nanosphere (*n*_A_ = 0). The reaction rate of an individual nanosphere can be calculated to be

$r=-J_{A}\times4\pi R^{2}$ (17)

where *R* is the radius of the nanosphere. The reaction rate of the entire solution is

$r_{\mathrm{total}}=r\times the number of the nanospheres$ (18)

In our work, the average size of the nanospheres was ~550 nm. We assume that these nanospheres were dispersed evenly in the reaction solution and the density *ρ* of MoO_3_ (4.69 g cm^−3^) can represent that of the MoO_3–_*_x_*/10% Ag nanospheres.

For 80 mg MoO_3–_*_x_*/10% Ag nanospheres, the number of the nanospheres = $\frac{V_{\mathrm{total}}}{V_{\mathrm{individual}}}=\frac{\frac{m}{\rho}}{\frac{4\pi R^{3}}{3}}=\frac{\frac{{80\times10}^{-3}}{4.69}}{\frac{4\pi\times\left( 550/2\times{10}^{-7} \right)^{3}}{3}}$ = 1.96 × 10^11^, where *V*_total_ is the total volume of the nanospheres, *V*_individual_ is the volume of an individual nanosphere, and *m* is the mass of the nanosphere sample added in the reaction solution.

The thickness of the boundary layer ($\delta_{n}$) can be calculated from Equations (16), (17), and (18) to be *δ*_n_ = 2.67 cm, which is too thick in a common PCNF reaction test. Such a large number also indicates that the process is reaction-controlled rather than diffusion-controlled.

As shown in Figure S19a,b, the photocatalytic activities rise slightly and then drop slightly with increasing N_2_ flow rates. When the N_2_ flow rate is slow, O_2_ can diffuse into the reaction system, which causes the decrease in the photocatalytic performance. When the N_2_ flow rate is high, the slight reduction is believed to result from the complexity of the real reaction situation. We reason that a high N_2_ flow rate will induce the formation of a large number of bubbles, which will in turn reduce the contact area between the photocatalyst and the proton source (H_2_O) and thus results in the decrease in the photocatalytic performance.

Different from the effect of the N_2_ flow rate, the stirring speed had a significant influence on the PCNF activity (Figure S19c,d). A low stirring speed led to the flocculation of the photocatalyst nanoparticles and reduced the contact probability between N_2_ molecules and the photocatalyst nanoparticles. The flocculation could be diminished by increasing the stirring speed. It became negligible when the stirring speed was higher than ~4,500 rpm regardless of how the N_2_ flow rate was changed. Under these conditions, the sluggish N_2_ diffusion kinetics had only a negligible effect on the photocatalytic performance.


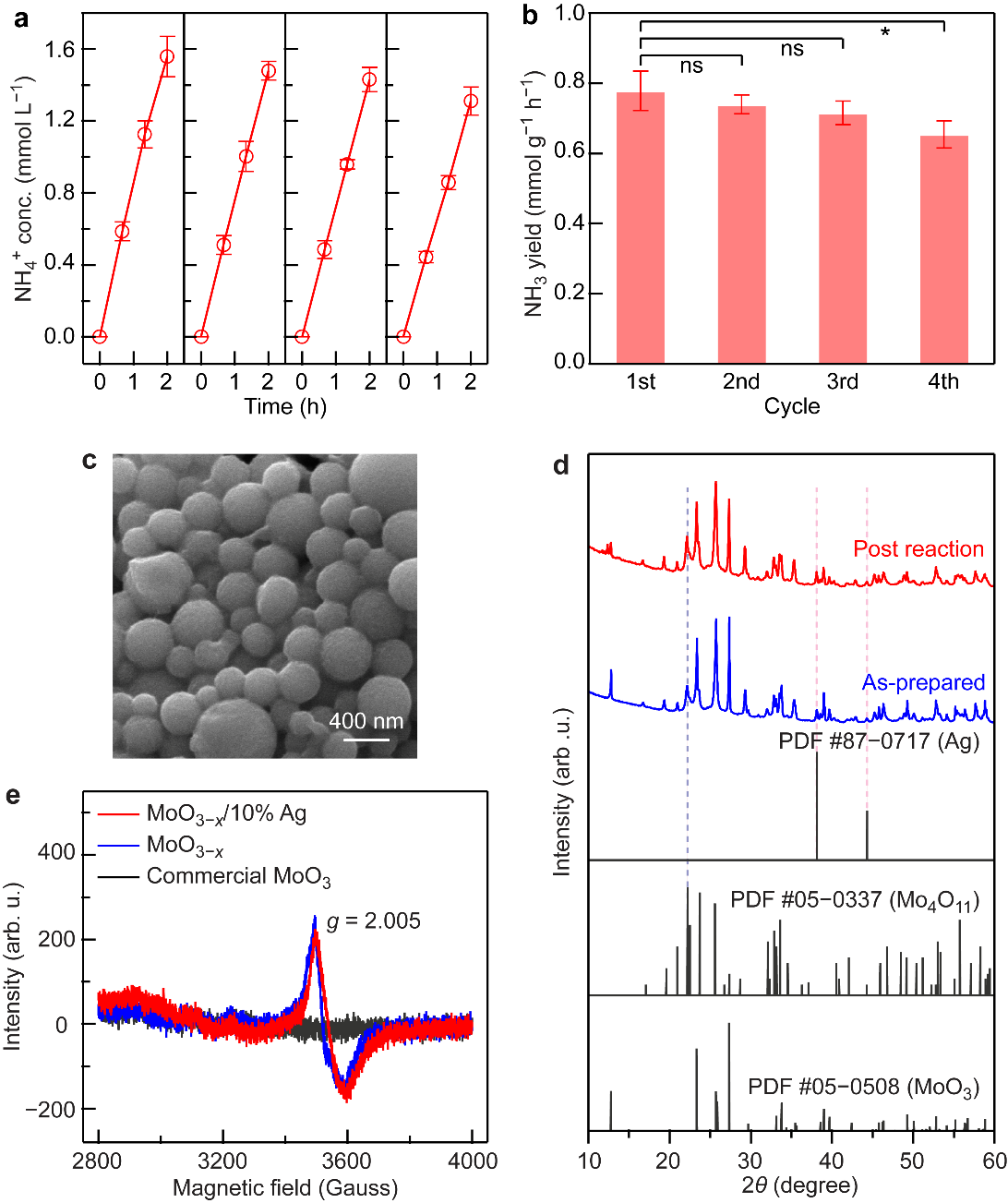


Figure S20. Cycling tests for PCNF. a) Time-dependent NH_3_ production in 4 cycles with the MoO_3–_*_x_*/10% Ag sample. b) NH_3_ yields obtained at each cycle. * represents *p* < 0.05, and “ns” means not significant. c) SEM image of the photocatalyst sample after 4 cycles of test. d) XRD patterns of the MoO_3–_*_x_*/10% Ag sample before and after 4 cycles of test. The standard powder diffraction patterns (JCPDS No. 05-0508 for orthorhombic α-MoO_3_, JCPDS No. 05-0337 for the Magneli Mo_4_O_11_ phase, and JCPDS No. 87-0717 for face-centered cubic Ag) are also displayed as references. e) EPR spectra of the commercial intrinsic MoO_3_ nanoparticle, MoO_3–_*_x_* nanosphere, and MoO_3–_*_x_*/10% Ag nanosphere samples. All the three samples were subjected to the cycling test conditions.


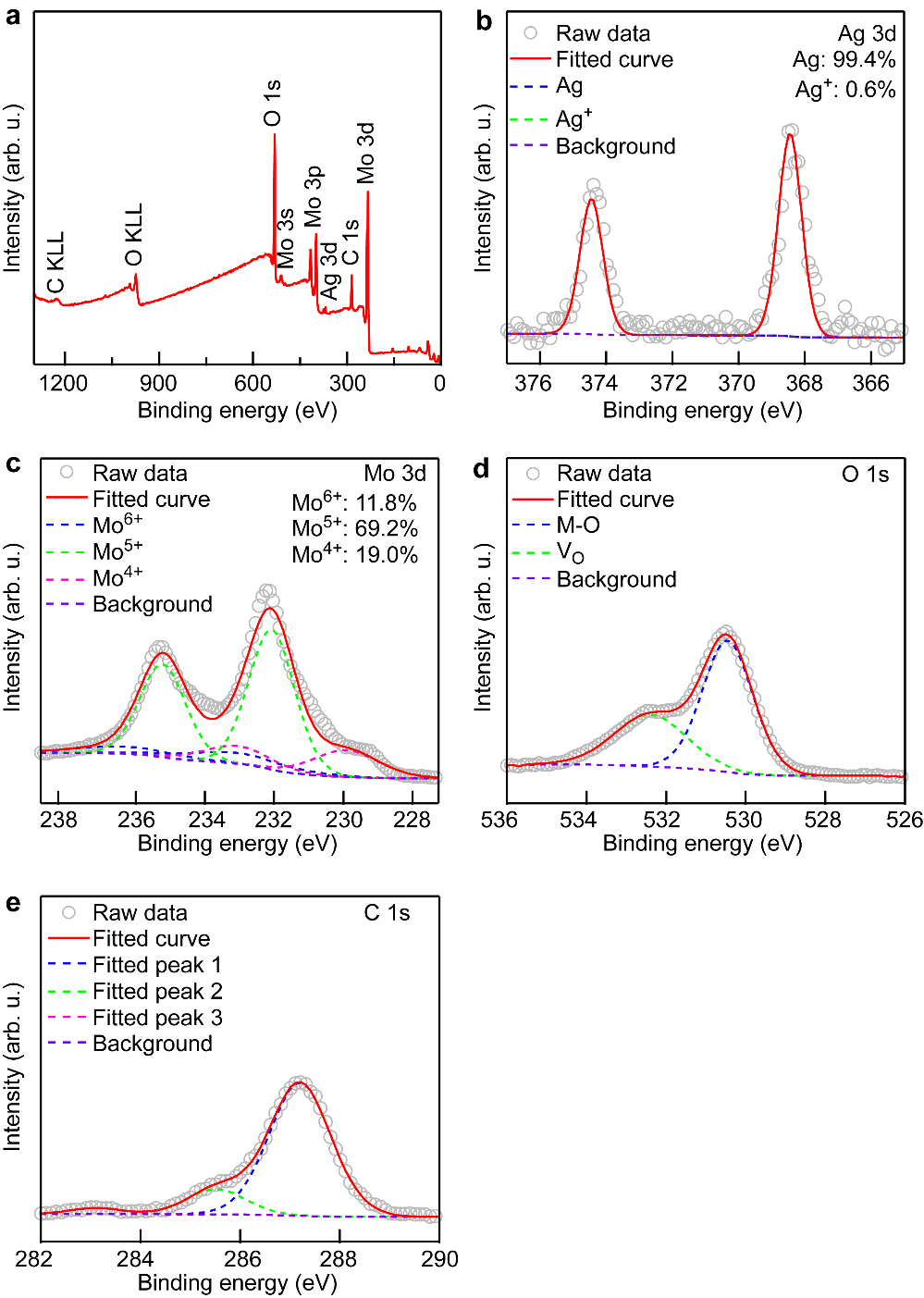


**Figure** **S21.** XPS spectra of the MoO_3–_*_x_*/10% Ag sample after 4 cycles of test. a) Survey XPS spectrum. b–e) High-resolution XPS spectra of Ag 3d (b), Mo 3d (c), O 1s (d), and C 1s (e). The blue dashed lines in (b) overlap with the red lines. The valence state of Mo was found from XPS to be 4.93.


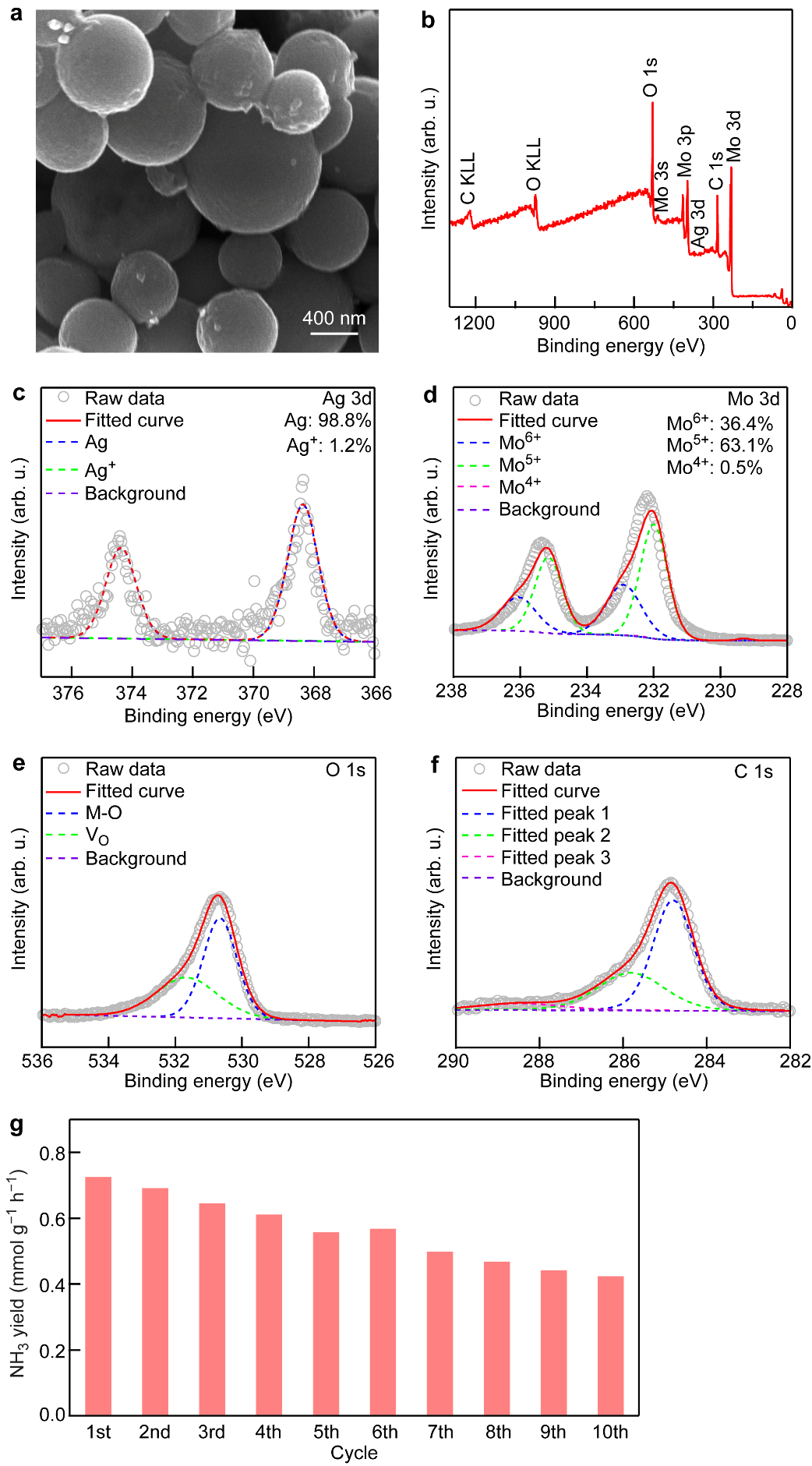


Figure S22. Extended cycling tests (10 cycles) with the MoO_3–_*_x_*/10% Ag sample. a) SEM image. b) Survey XPS spectrum. c–f) High-resolution XPS spectra of Ag 3d (c), Mo 3d (d), O 1s (e), and C 1s (f). g) NH_3_ yields obtained at each cycle. The blue dashed lines in (c) overlap with the red lines. The valence state of Mo was found from XPS to be 5.36.


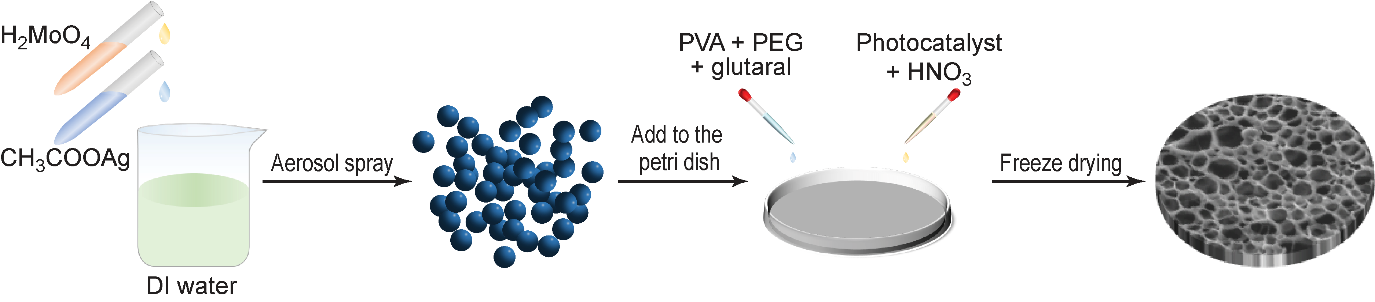


Figure S23. Schematic showing the synthesis process of the MoO_3–_*_x_*/10% Ag/PVA solar absorber film.


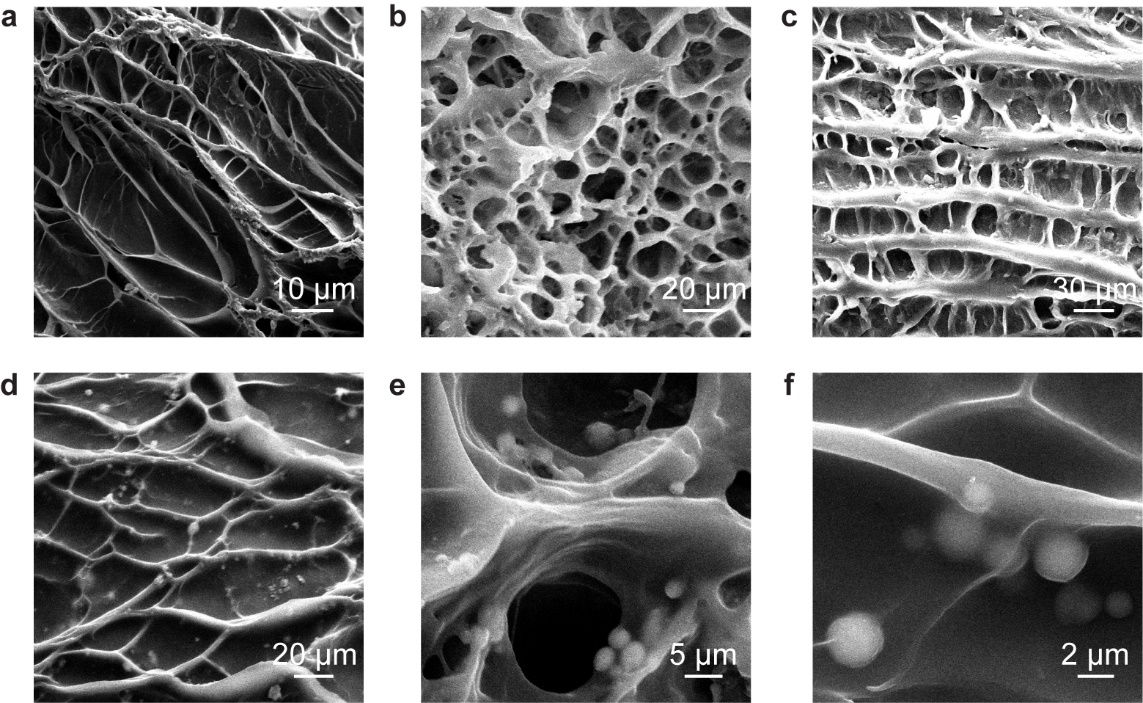


**Figure** **S24.** SEM images. a–c) Pure PVA films. d–f) MoO_3–_*_x_*/10% Ag/PVA solar absorber film.

The density of a non-porous PVA film was measured to be 1.061 g cm^−3^. The apparent densities of the solar absorber films were found to be 0.514 g cm^−3^ (without the photocatalyst) and 0.602 g cm^−3^ (with the photocatalyst). The corresponding pore volume percentages were calculated to be 51% and 43%, respectively.


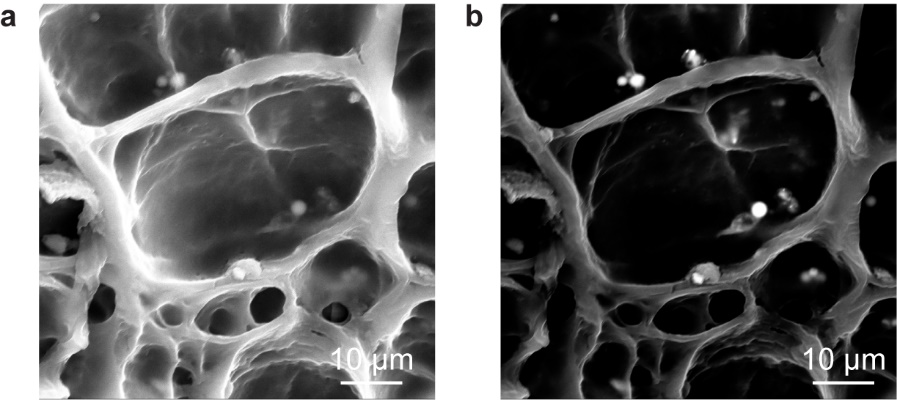


**Figure** **S25.** High-magnification SEM images of the MoO_3–_*_x_*/10% Ag/PVA solar absorber film. a) Secondary electron detection mode. b) Corresponding backscattered electron detection mode at the same position.


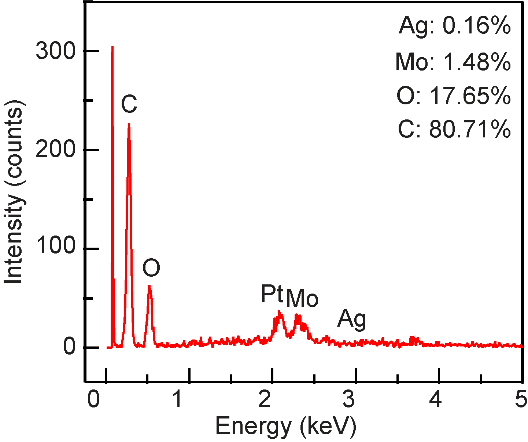


**Figure** **S26.** EDX spectrum of the MoO_3–_*_x_*/10% Ag/PVA solar absorber film. The EDX spectrum was measured during SEM imaging. The measured Ag, Mo, C, and O contents are labeled in the plot. The Pt peak was originated from the sputtered Pt nanoparticles before SEM observation to enhance the electrical conductivity of the solar absorber film.


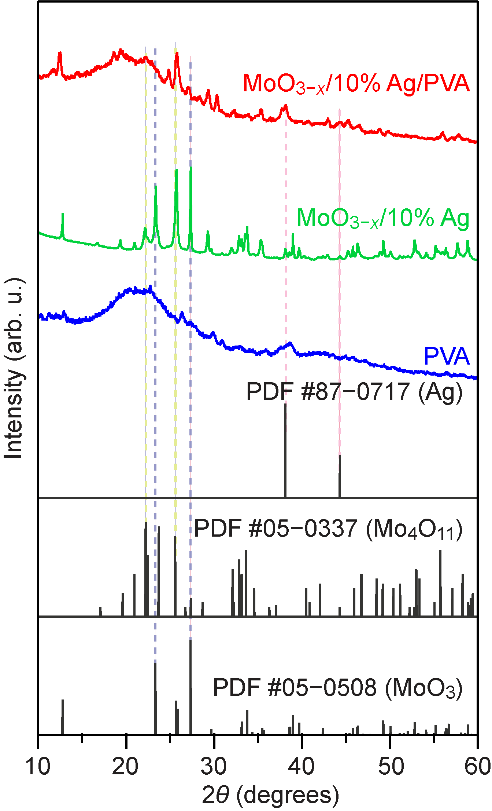


**Figure** **S27.** XRD patterns. The MoO_3–_*_x_*/10% Ag/PVA solar absorber film, the MoO_3–_*_x_*/10% Ag nanospheres, and the PVA film were measured for comparison.


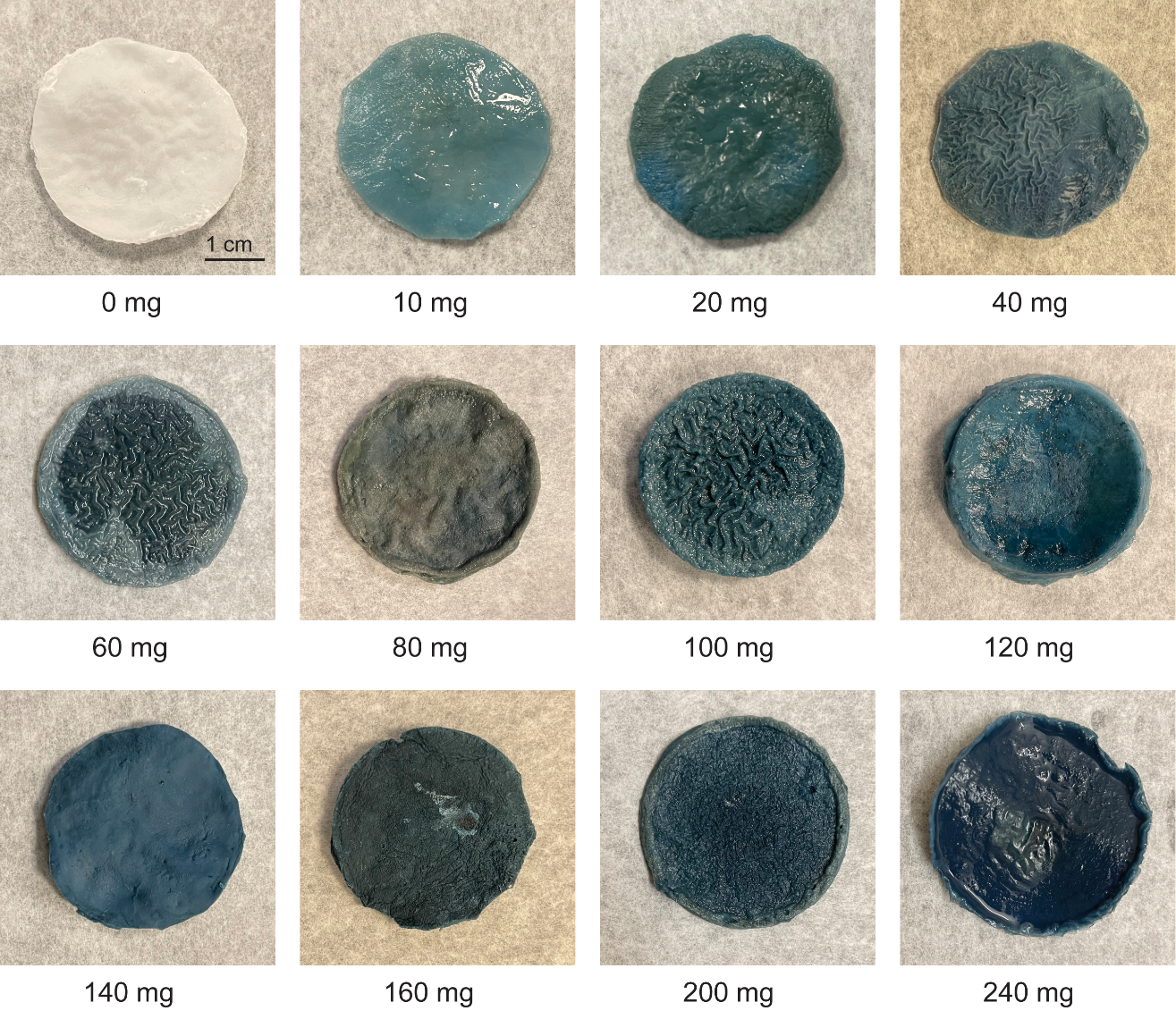


**Figure** **S28.** Photographs of the MoO_3–_*_x_*/10% Ag/PVA solar absorber films with different photocatalyst loading amounts.


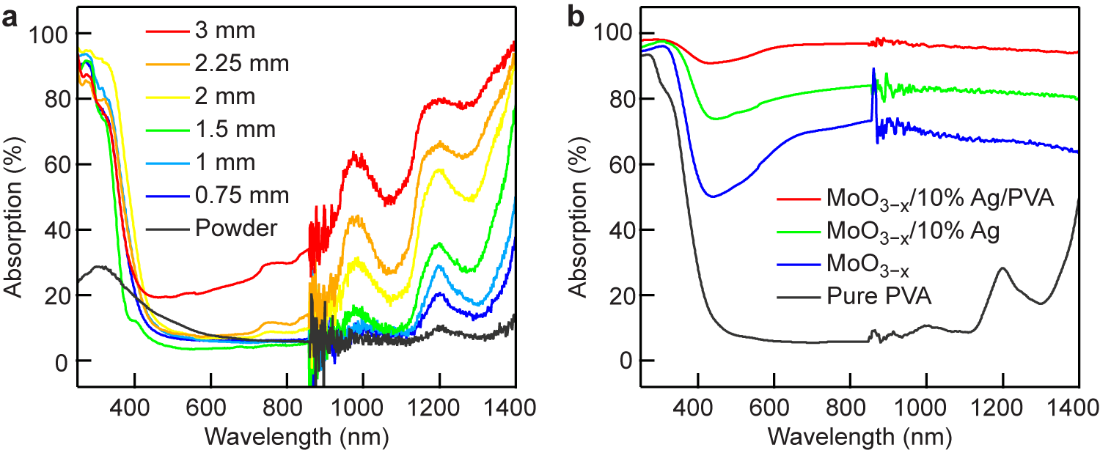


**Figure** **S29.** Light absorption spectra of the film samples. a) Pure PVA films with different thicknesses. b) Pure PVA film with 1 mm thickness, MoO_3–_*_x_* photocatalyst powder, MoO_3–_*_x_*/10% Ag photocatalyst powder, and MoO_3–_*_x_*/10% Ag/PVA solar absorber film with 1 mm thickness. The loading amount of the MoO_3–_*_x_*/10% Ag photocatalyst in the solar absorber film is 80 mg.


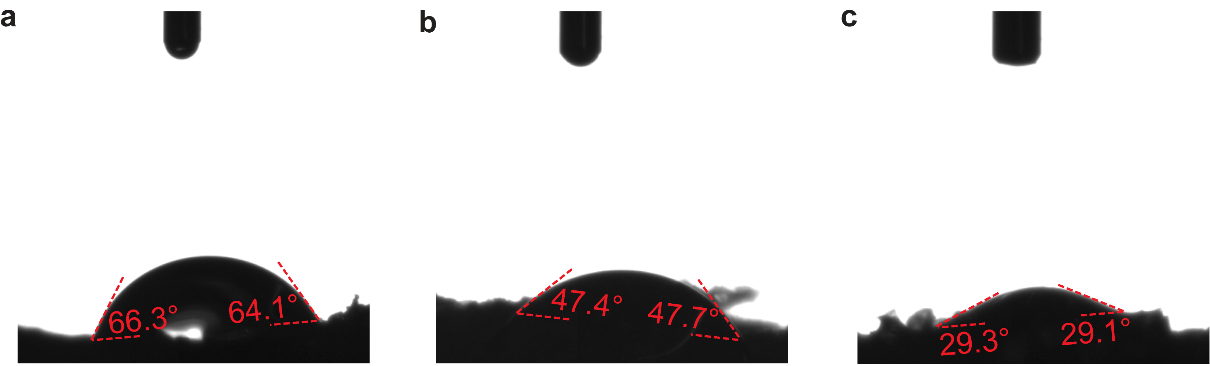


**Figure** **S30.** Photographs of water droplets on the different samples. a) Pure PVA film. b) Solar absorber film with 30 mg photocatalyst loading amount. c) Solar absorber film with 80 mg photocatalyst loading amount. Because of the inherent surface roughness of the solar absorber films, the baseline for water contact angle calculation was determined from the droplet contour and film profile.


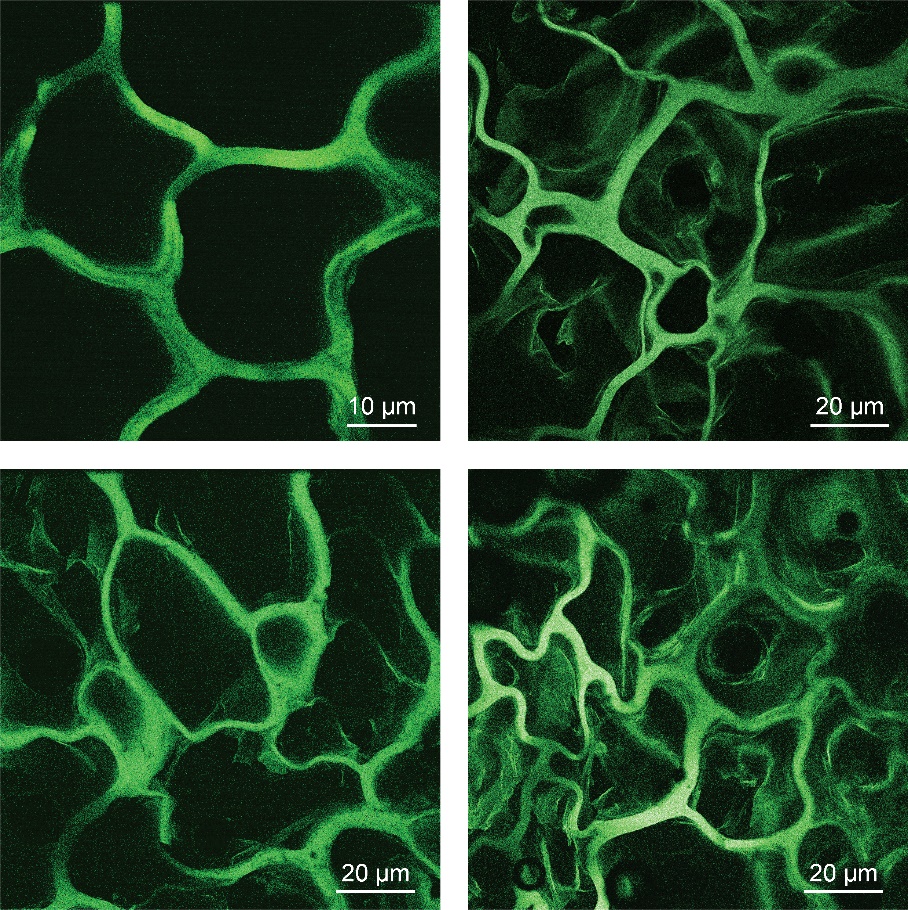


**Figure** **S31.** CLSM images of the MoO_3–_*_x_*/10% Ag/PVA solar absorber film.


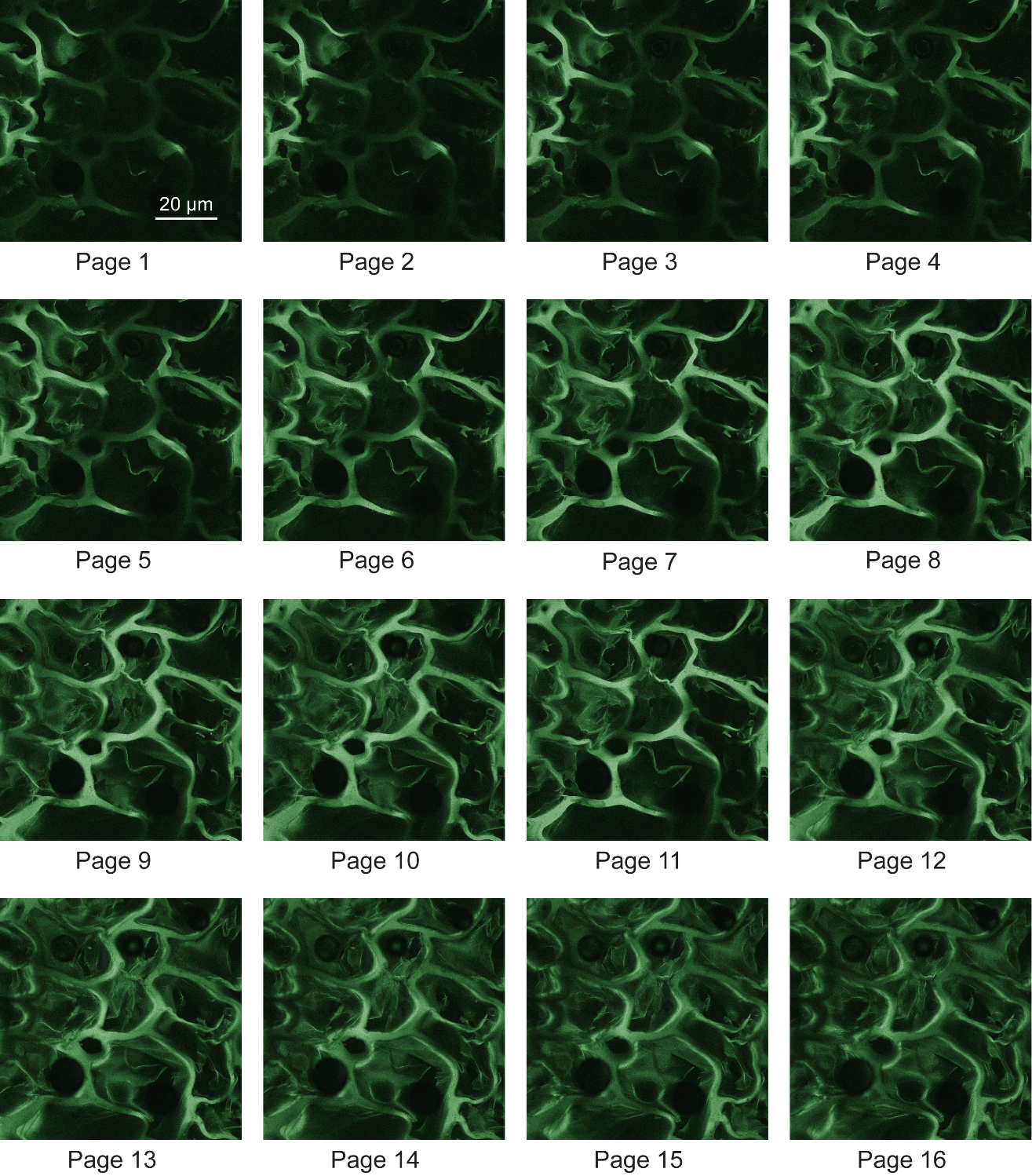


**Figure** **S32.** CLSM images of the MoO_3–_*_x_*/10% Ag/PVA solar absorber film recorded at different depths.


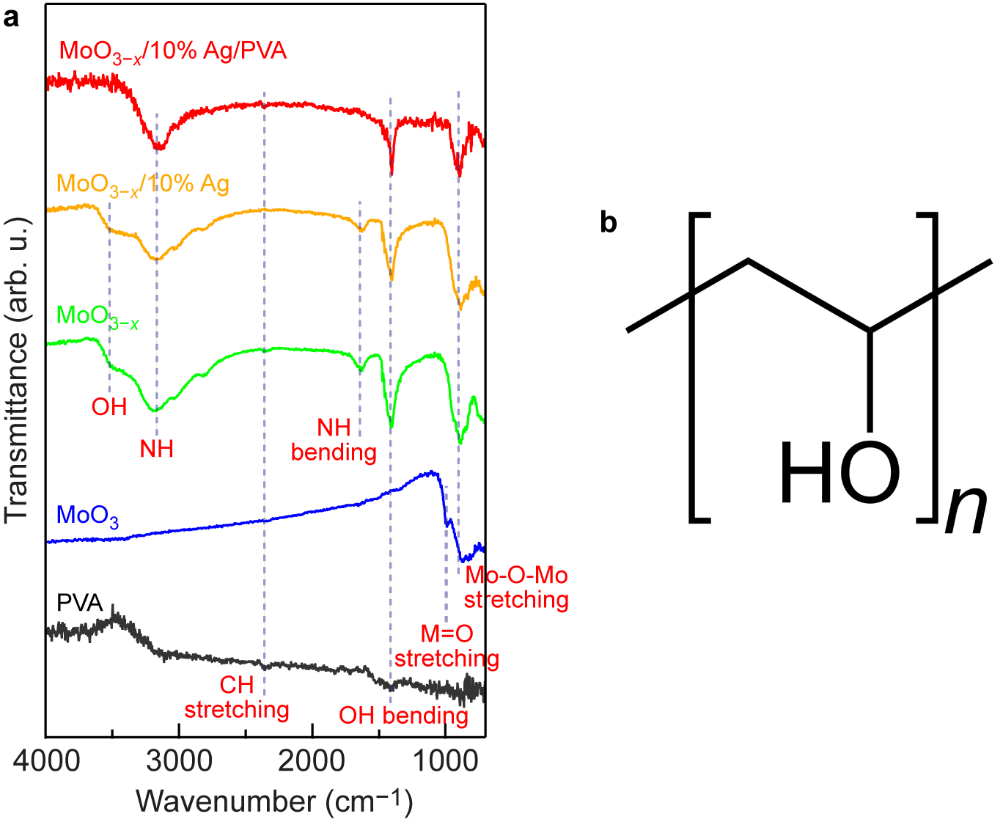


**Figure** **S33.** FTIR spectra. a) FTIR spectra of the pure PVA film, intrinsic commercial MoO_3_, MoO_3–_*_x_*, MoO_3–_*_x_*/10% Ag, and MoO_3–_*_x_*/10% Ag/PVA solar absorber film. b) Molecular structure of PVA molecules.


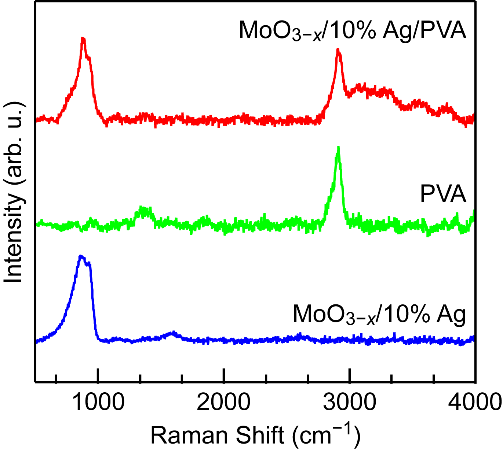


**Figure** **S34.** Raman spectra of the MoO_3–_*_x_*/10% Ag sample, pure PVA powder, and MoO_3–_*_x_*/10% Ag/PVA solar absorber film.


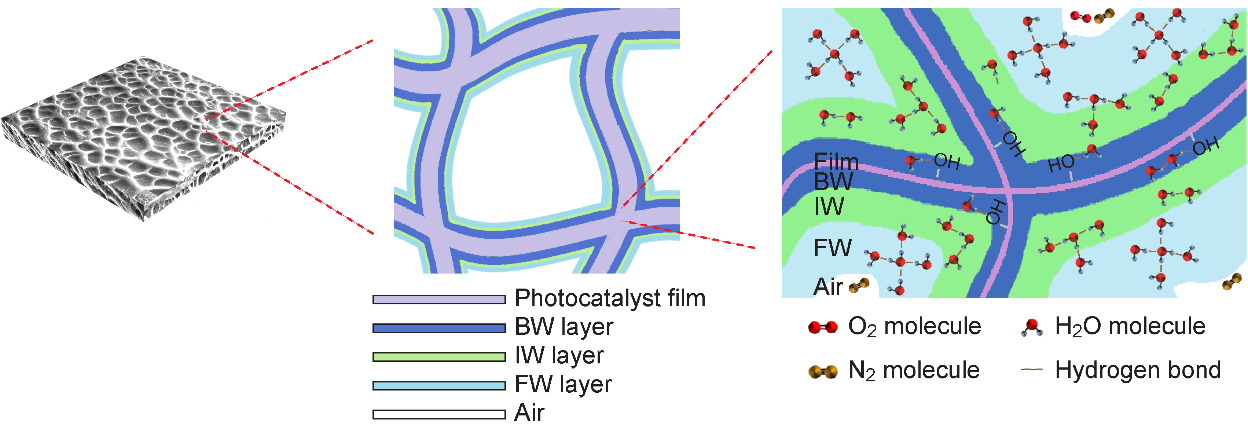


**Figure** **S35.** Schematic of the water states in the porous network of the solar absorber film. It shows water–PVA bonding, weakened water–water bonding, and normal water–water bonding. Free water (FW, light blue color) has no interaction with the PVA network. Bound water (BW, dark blue color) exhibits strong interaction with the functional groups on the surface of the PVA network. In the intermediate region between BW and FW exists intermediate water (IW, green color). BW, together with IW, forms the confined water (CW) layer.

The adsorbed interfacial water molecules near the surface were probed by ATR-FTIR experiments. As shown in Figure 3g, the presence of water results in a broad vibrational spectrum between 3,000 and 3,800 cm**^−^**^1^ against the background spectrum of the clean diamond, which can be attributed to the OH bond stretching. The spectrum can be deconvoluted into three peaks centered at ~3,200, ~3,400, and ~3,600 cm**^−^**^1^, which are ascribed to the OH stretching mode associated with the tetrahedral structure of bulk water molecules, hydrogen-bonded OH straddling the interface, and non-hydrogen-bonded OH pointing toward the surface, respectively.^[10,11]^ Based on the comparison, the peak at ~3,200 cm**^−^**^1^ (peak I) represents free water (FW), which means ordered or networked water owing to the strong hydrogen bond interaction. The peak at ~3,400 cm**^−^**^1^ (peak II) represents confined water (CW), which means disordered water that is affected by the interactions with PVA. The peak at ~3,600 cm**^−^**^1^ (peak III) indicates the hydroxyl functional group at the surface of PVA. The fabricated solar absorber film shows a larger intensity of peak II compared to peak I and III, indicating that CW with weakly hydrogen-bonded structures is dominant.^[12]^ Comparison with the ATR–FTIR spectrum of bulk water (Figure 3h) revealed that the structural configuration of the adsorbed thin water layer in the solar absorber film is drastically different from that of liquid water.^[10]^ First, the disappearance of the peak III at ~3,600 cm**^−^**^1^ indicates that there are no non-hydrogen-bonded OH in bulk water.^[13]^ Second, a distinct redshift of the two fitted peaks was observed,^[14]^ which can be attributed to the abundance of hydrogen bonding interaction in bulk water. The exact position and shape of the peaks are a complicated function of the hydrogen bond dynamics.^[12]^ With the increasing strength of hydrogen bonding, the OH stretching peak position will gradually redshift. Third, the intensity of peak I is much larger than that of peak II, indicating that FW with strong hydrogen bond interaction is dominant in bulk water.


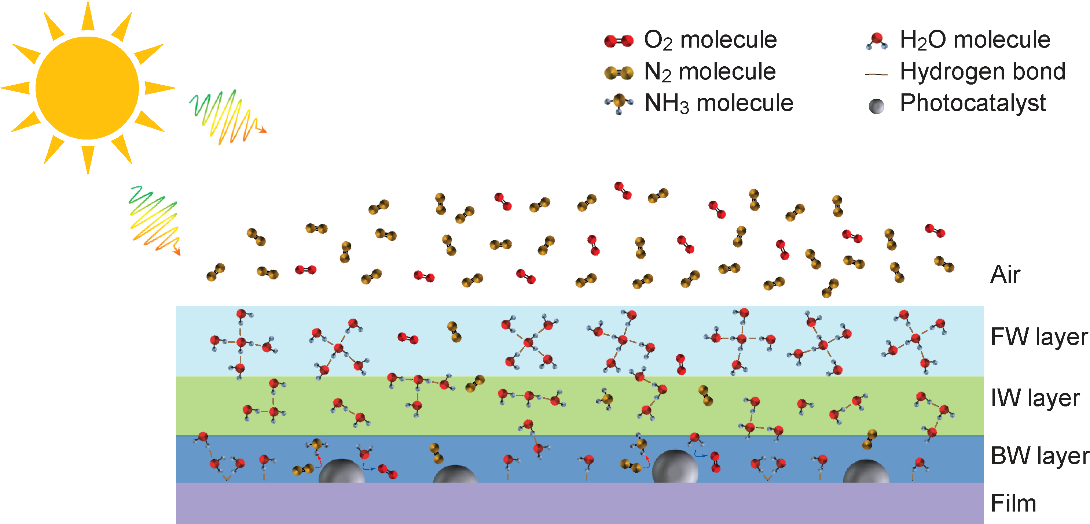


Figure S36. Schematic displaying the PCNF reaction in the film system.


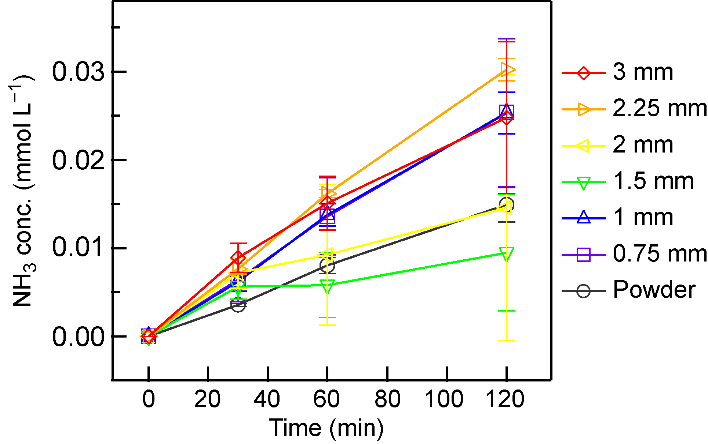


**Figure** **S37.** Time-dependent production of NH_3_ with the pure PVA powder and PVA films with different thicknesses.


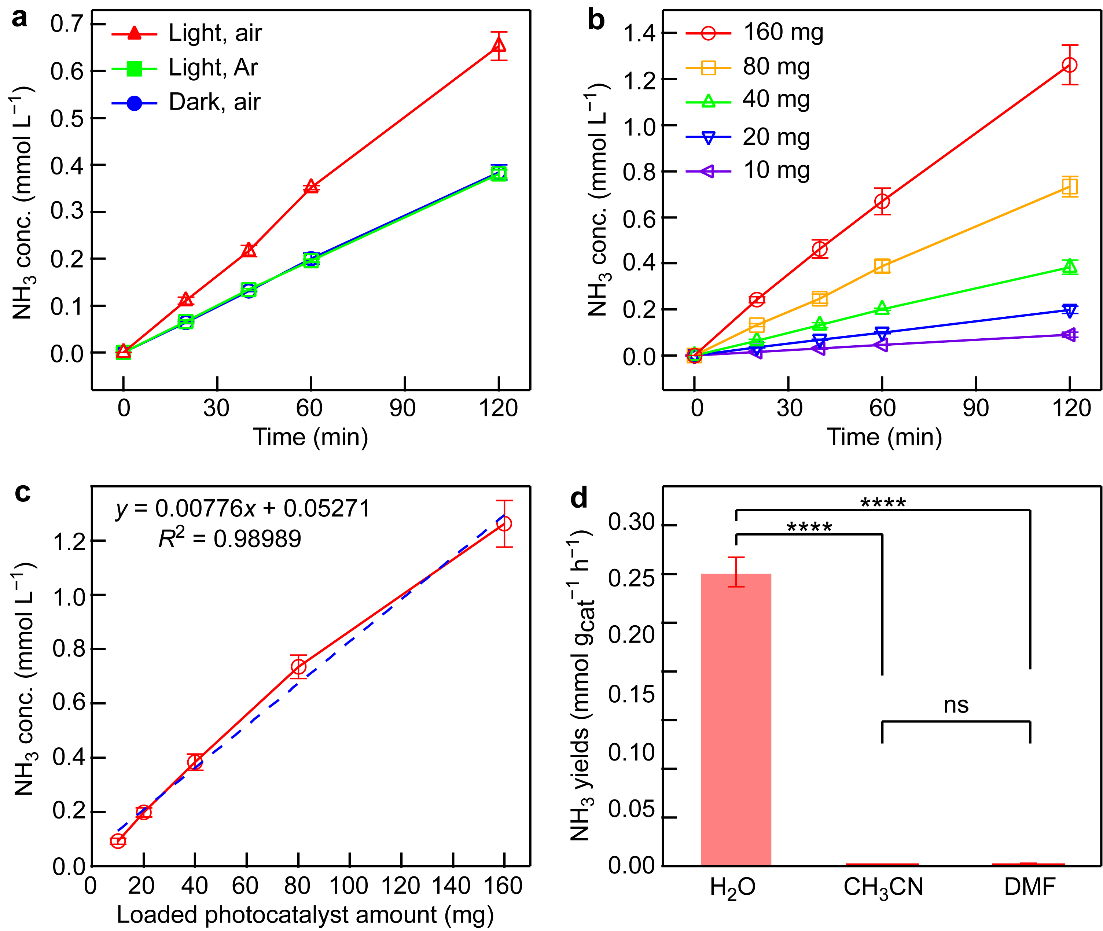


**Figure** **S38.** Control experiments of the photocatalyst solar absorber films for PCNF. a) Time-dependent production of NH_3_ under different conditions. b) Time-dependent release of NH_3_ with different photocatalyst loading amounts under the dark conditions. c) Linear relationship between the NH_3_ concentration after 2 h and the photocatalyst loading amount under the dark conditions. d) Photocatalytic NH_3_ yields in the different solvents under light illumination. The error bars on the data points for the NH_3_ yields obtained from the reactions with either CH_3_CN or DMF as the solvent are too small to be seen. **** represents *p* < 0.0001, and “ns” means not significant.

The photocatalyst showed NH_3_ production in Ar atmosphere and under the dark conditions (Figure S16a), which is believed to result from the gradual release of NH_3_ that was pre-adsorbed on the MoO_3–_*_x_*/10% Ag photocatalyst in water. Similar phenomena have also been observed in Figure S38a. The pre-adsorbed NH_3_ comes from the molybdic acid precursor as commercial molybdic acid is synthesized by an ammonium molybdate nitric acid neutralization method and contains a certain amount of ammonium molybdate.^[15]^ During the photocatalyst preparation, the thermal decomposition of ammonium molybdate produced NH_3_. The nanograins in the photocatalyst nanosphere adsorbed NH_3_ molecules, causing NH_3_ to be encapsulated in the photocatalyst nanospheres. For the powder system in Figure S16a, the strong sonication and stirring accelerated the NH_3_ release kinetics. While for the film system in Figure S38a, the adsorbed NH_3_ was challenging to be removed and was gradually released during the photocatalytic process owing to the absence of proper driving methods. To exclude the effect of NH_3_ pre-adsorbed in the photocatalyst, the plasmonic photocatalyst was loaded into PVA films at different amounts and the relationship between the NH_3_ release and the photocatalyst loading amount under the dark conditions was established (Figure S38b). A well-fitted linear relationship was observed (Figure S38c), providing the criterion for distinguishing the photocatalytic and non-photocatalytic contributions to the NH_3_ production. All the photocatalytic performances of the subsequent experiments for the film systems in this work were the results after the deduction of the non-photocatalytic NH_3_ release unless otherwise specified.


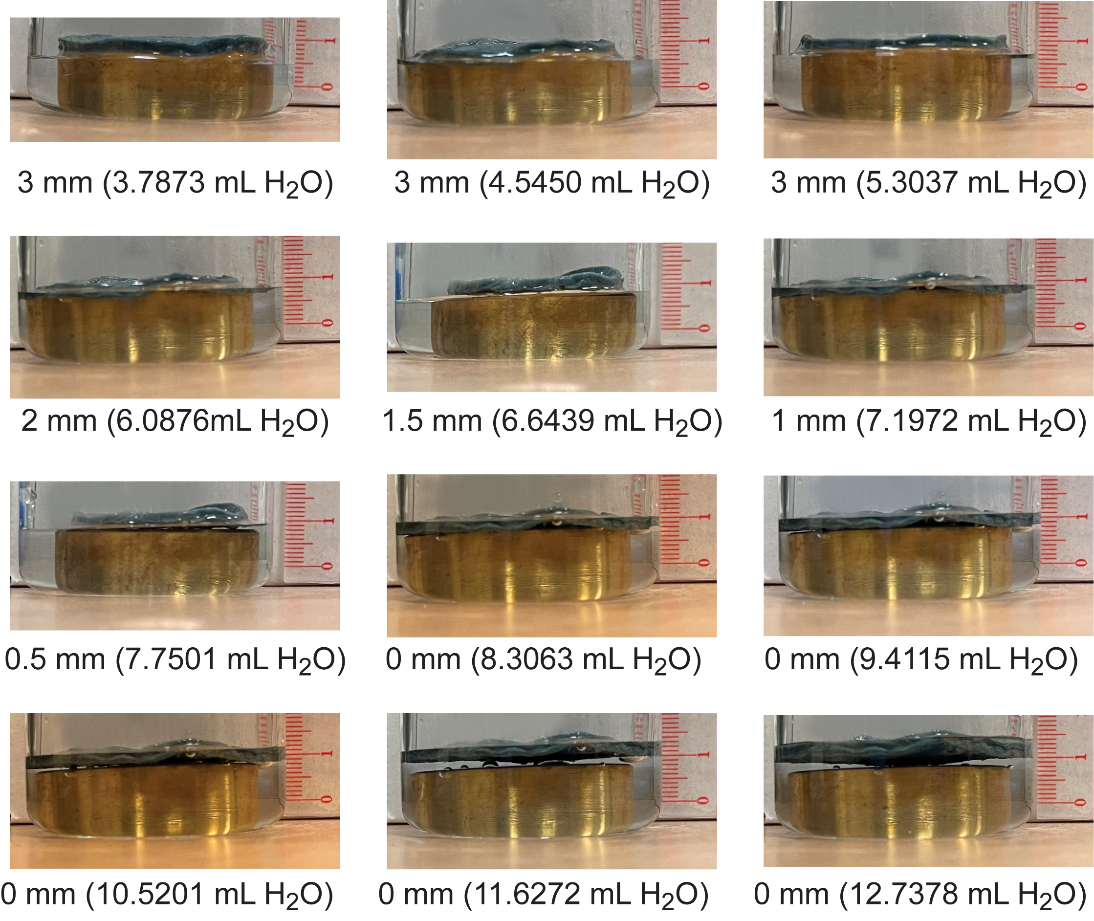


**Figure** **S39.** Photographs of the experimental setup to study the effect of the height of the film above the water surface on the photocatalytic performance. The solar absorber films were supported by a thick Cu cylinder. The numbers before the parentheses indicate the heights of the film above the water surface.


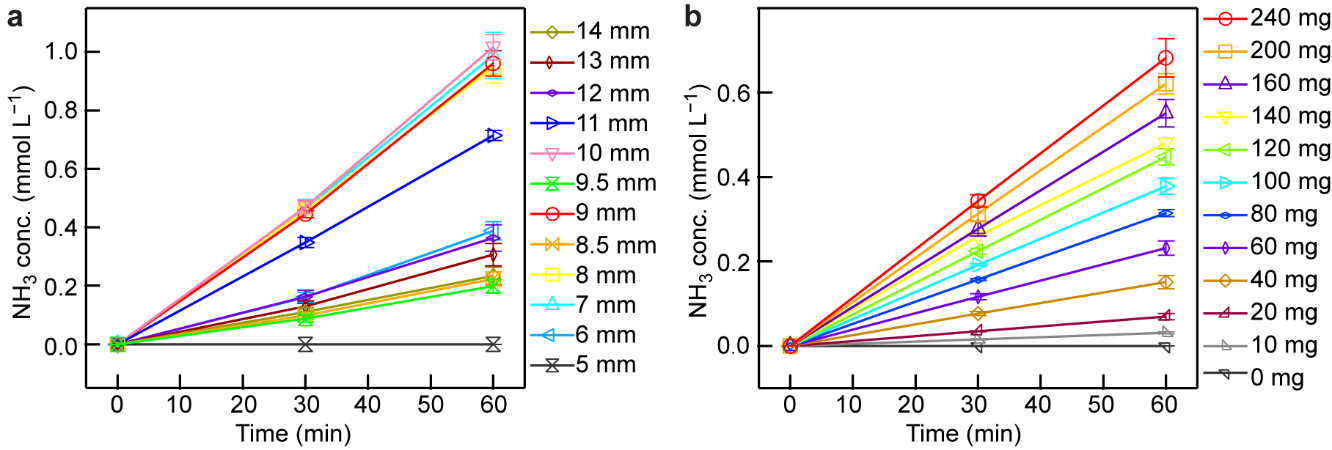


**Figure** **S40.** Time-dependent production of NH_3_ under different conditions. a) For the solar absorber film systems with water heights of 5–14 mm. b) For the bilayer solar absorber film systems with different photocatalyst loading amounts.

A smaller value of the height of the film above the water surface indicates that the majority of the film was submerged below the water surface and the most pores inside the film were filled with water. This situation significantly impeded the mass transfer process of N_2_ molecules because of the relatively low solubility and diffusion coefficient of N_2_ in water. Although a higher value of the height of the film above the water surface ensured the effective contact between N_2_ molecules and the surface of the photocatalyst nanoparticles, the film under this condition could not support efficient water from the bottom to reach the top surface region of the film because of gravity, thereby limiting the efficient conversion of absorbed solar energy into chemical energy on the photocatalyst at the upper portion of the film.


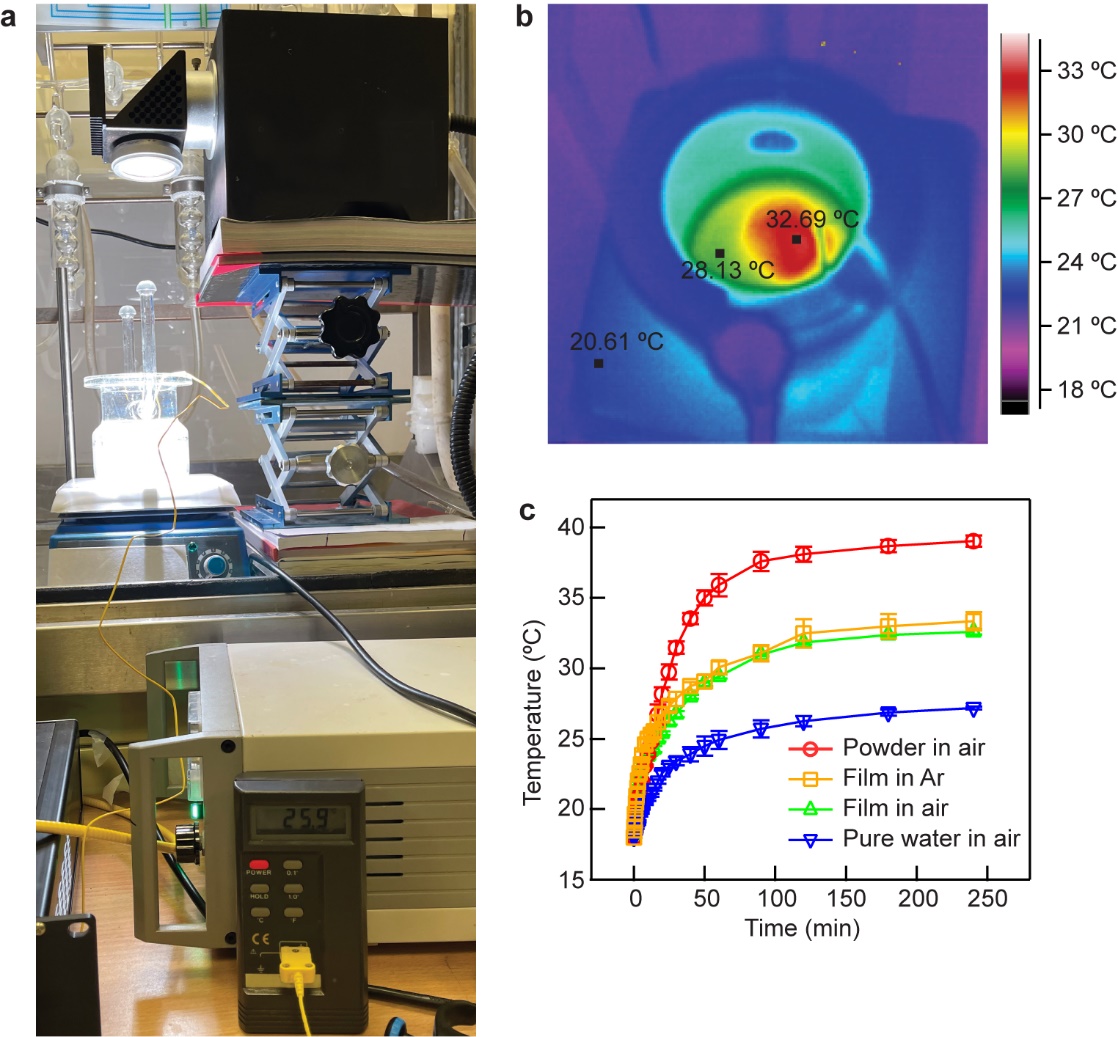


**Figure** **S41.** Temperature evolution experiments. a) Photograph of the experimental setup. b) Infrared image of a photocatalyst solar absorber film floating in the photocatalytic reactor during the reaction. c) Time-dependent temperature evolutions of deionized water, the powder system in water, and the film system in water under air or Ar atmosphere.


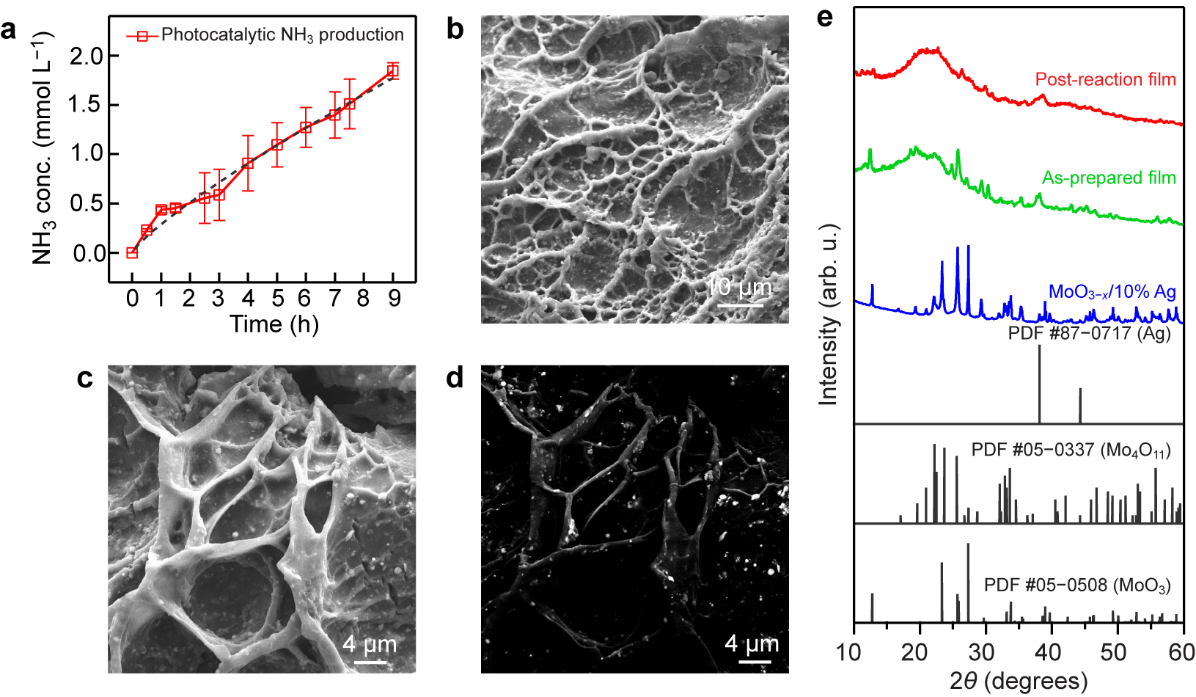


**Figure** **S42.** Photocatalysis and structure characterization of the MoO_3–_*_x_*/10% Ag/PVA solar absorber film after 9 h photocatalytic nitrogen fixation reaction. a) Time-dependent photocatalytic production of NH_3_ for the solar absorber film. b) SEM image. c) High-magnification SEM image. d) High-magnification backscattered electron image. e) XRD patterns of the solar absorber film before and after the reaction. The standard powder diffraction patterns (JCPDS No. 05–0508 for orthorhombic α-MoO_3_, JCPDS No. 05–0337 for the Magneli Mo_4_O_11_ phase, and JCPDS No. 87–0717 for face-centered cubic Ag) are also displayed as references.

**Table** **S1.** Calculated grain sizes for the MoO_3–_*_x_*/10% Ag sample.

| 2*θ*  (°) | FWHM  (°) | Grain size  (nm) | 2*θ*  (°) | FWHM  (°) | Grain size  (nm) |
| --- | --- | --- | --- | --- | --- |
| 12.76916 | 0.34094 | 23.18604 | 32.68248 | 0.34048 | 24.0447 |
| 16.7792 | 0.34045 | 23.325 | 33.87318 | 0.34072 | 24.10249 |
| 19.51642 | 0.34052 | 23.40935 | 35.26916 | 0.34094 | 24.17843 |
| 22.07573 | 0.34055 | 23.50341 | 39.10128 | 0.34045 | 24.48718 |
| 20.70712 | 0.34074 | 23.43738 | 38.12097^a)^ | 0.19079 | 43.56473 |
| 23.4854 | 0.34061 | 23.55759 | 39.99088 | 0.34093 | 24.52104 |
| 25.72993 | 0.34102 | 23.63 | 43.04288 | 0.34049 | 24.80186 |
| 27.30383 | 0.34053 | 23.74071 | 46.24544 | 0.34062 | 25.07854 |
| 29.34307 | 0.3405 | 23.84966 | 49.31113 | 0.34091 | 25.35591 |
| 31.82026 | 0.34044 | 23.99527 | 52.67792 | 0.34047 | 25.74696 |

^a)^This diffraction peak belongs to Ag.

The grain size can be calculated according to

$\tau=\frac{K\lambda}{\beta_{\mathrm{FWHM}}\cos\theta}$ (19)

where *τ* is the grain size, *K* is a dimensionless shape factor, with a a typical value of 0.89, *λ* is the X-ray wavelength, *β*_FWHM_ is the line broadening at half the maximal intensity (FWHM) in radian, and *θ* is the Bragg angle in radian.

In this work, the average grain size of MoO_3–_*_x_* is ~24.1 nm, and that of Ag nanoparticles is ~43.6 nm.

**Table** **S2.** Charge carrier concentrations and electronic band structures of commercial MoO_3_ and the MoO_3–_*_x_*/Ag nanosphere samples with different Ag molar percentages.

|  | Charge carrier concentration  (cm^−3^) | Flatband potential  (V vs Ag/AgCl) | Conduction band potential  (V vs Ag/AgCl) | Conduction band potential  (V vs NHE) |
| --- | --- | --- | --- | --- |
| Commercial MoO_3_ | 1.02 × 10^21^ | –0.021 | –0.121 | 0.076 |
| MoO_3–_*_x_* | 1.06 × 10^22^ | –0.176 | –0.276 | –0.079 |
| MoO_3–_*_x_*/1% Ag | 1.11 × 10^22^ | –0.176 | –0.276 | –0.079 |
| MoO_3–_*_x_*/5% Ag | 1.34 × 10^22^ | –0.180 | –0.280 | –0.083 |
| MoO_3–_*_x_*/9% Ag | 1.56 × 10^22^ | –0.184 | –0.284 | –0.087 |
| MoO_3–_*_x_*/10% Ag | 1.62 × 10^22^ | –0.185 | –0.285 | –0.088 |
| MoO_3–_*_x_*/11% Ag | 1.66 × 10^22^ | –0.185 | –0.285 | –0.088 |
| MoO_3–_*_x_*/13% Ag | 1.80 × 10^22^ | –0.188 | –0.288 | –0.091 |

**Table** **S3.** Specific values of the equivalent circuit used to fit the impedance data.

|  | *R*_s_ (Ω) | *R*_cd_ (Ω) | *R*_ct_ (Ω) | *C*_cd_ (μF) | *C*_ct_ (μF) | *Z*_w_ (Ω) |
| --- | --- | --- | --- | --- | --- | --- |
| MoO_3–_*_x_* | 70.60 | 85.10 | 45999 | 5.27 | 1.50 |  |
| MoO_3–_*_x_*/1% Ag | 38.49 | 64.47 | 3458 | 11.91 | 11.36 |  |
| MoO_3–_*_x_*/5% Ag | 37.74 | 56.59 | 1236 | 17.08 | 28.48 |  |
| MoO_3–_*_x_*/9% Ag | 25.70 | 18.52 | 1220 | 9.32 | 2.28 | *Z*_w_-R^a)^: 2116  *Z*_w_-T^b)^: 35.89  *Z*_w_-P^c)^: 0.29 |
| MoO_3–_*_x_*/10% Ag | 29.80 | 10.54 | 1057 | 0.54 | 0.23 | *Z*_w_-R: 1685  *Z*_w_-T: 21.77  *Z*_w_-P: 0.39 |
| MoO_3–_*_x_*/11% Ag | 25.11 | 12.61 | 1024 | 0.76 | 1.83 | *Z*_w_-R: 1405  *Z*_w_-T: 12.88  *Z*_w_-P: 0.25 |
| MoO_3–_*_x_*/11% Ag | 28.63 | 9.91 | 886 | 0.55 | 0.23 | *Z*_w_-R: 1024  *Z*_w_-T: 12.04  *Z*_w_-P: 0.39 |

^a)^Z_w_-R is the diffusion impedance. ^b)^Z_w_-T is the diffusion interpretation. ^c)^*Z*_w_-P is the exponential factor.

**Table** **S4.** Product composition for the N_2_ photofixation with the MoO_3–_*_x_*/10% Ag sample under simulated sunlight for 0.5 h.

| Product | Amount in N_2_ atmosphere (µmol) | Amount in Ar atmosphere (µmol) |
| --- | --- | --- |
| NH_3_ | 12.198 | 0.002 |
| N_2_H_4_ | 0.003 | 0.001 |
| H_2_ | 2.827 | 4.635 |
| O_2_ | 10.903 | 2.436 |

The reaction conditions were as follows: 20 mg MoO_3–_*_x_*/10% Ag sample, 50 mL deionized water, 10 sccm N_2_ flow rate, and 5,000 rpm stirring speed. The reaction equations for PCNF and the hydrogen evolution reaction in pure water were

$2N_{2}+6H_{2}O\to4NH_{3}+3O_{2}$ (20)

$2H_{2}O\to2H_{2}+O_{2}$ (21)

The detected reduction products, 12.198 µmol NH_3_ and 2.827 µmol H_2_, were expected to correspond to 10.562 µmol O_2_. The expected amount of the oxidation product agreed well with the measured amount at a difference of 3%. The reaction products were therefore in a good stoichiometric ratio.

**Table** **S5.** Measured AQEs of the MoO_3–_*_x_*/10% Ag and MoO_3–_*_x_* samples under the optimal conditions.

|  | MoO_3–_*_x_*/10% Ag sample | | | | MoO_3–_*_x_* sample | | | |
| --- | --- | --- | --- | --- | --- | --- | --- | --- |
| Wave-length (nm) | Produced NH_3_  (μmol L^−1^ h^−1^) | Light power density  (mW cm^−2^) | Light power  *P* (mW) | AQE  (%) | Produced NH_3_  (μmol L^−1^ h^−1^) | Light power density  (mW cm^−2^) | Light power  *P* (mW) | AQE  (%) |
| 350 | 3.89 | 1.5 | 61.08 | 0.145 | 3.21 | 1.3 | 52.94 | 0.139 |
| 380 | 10.73 | 4.3 | 175.10 | 0.129 | 7.79 | 3.9 | 158.81 | 0.104 |
| 420 | 27.57 | 10.2 | 415.34 | 0.126 | 14.21 | 10.1 | 411.27 | 0.065 |
| 475 | 36.17 | 12.4 | 504.93 | 0.121 | 14.66 | 12.4 | 504.93 | 0.049 |
| 520 | 45.31 | 13.7 | 557.86 | 0.125 | 19.35 | 13.4 | 545.65 | 0.054 |
| 550 | 43.52 | 12.4 | 504.93 | 0.125 | 20.37 | 10.8 | 439.78 | 0.067 |
| 600 | 47.41 | 12.2 | 496.78 | 0.127 | 21.88 | 8.6 | 350.19 | 0.083 |
| 650 | 57.06 | 13.3 | 541.58 | 0.129 | 25.48 | 8.9 | 362.41 | 0.086 |
| 700 | 58.97 | 13.3 | 541.58 | 0.124 | 28.66 | 8.8 | 358.34 | 0.091 |
| 808 | 7.73 | / | 63.14 | 0.121 | 6.68 | / | 67.62 | 0.098 |
| 980 | 2.81 | / | 52.75 | 0.118 | 2.28 | / | 56.83 | 0.090 |

Take the determination of the AQE at 350 nm for the MoO_3–_*_x_* /10% Ag sample as an example. The diameter of the reactor was 7.2 cm. The illumination area of the solution was

$$S=\pi\times(\frac{d}{2})^{2}=3.14\times{(\frac{7.2}{2})}^{2}=40.72 cm^{2}$$

The light power was

$$P=I\times S=1.5\times40.72=61.08 \mathrm{mW}$$

The number of incident photons was

$$N_{\mathrm{incident}}=\frac{E_{\mathrm{in}}\lambda_{\mathrm{in}}}{hc}=\frac{Pt\lambda_{\mathrm{in}}}{hc}=\frac{61.08\times{10}^{-3}\times3600\times350\times{10}^{-9}}{6.63\times{10}^{-34}\times3\times{10}^{8}}=3.87\times{10}^{20}$$

In the equation above, *P* is the light power, *t* is the illumination time (1 h = 3,600 s), *λ*_in_ is the light wavelength, *h* is Planck’s constant, and *c* is the speed of light in free space. The number of reacted electrons was

$$N_{\mathrm{re}}=3\times number of the produced NH_{3}\mathrm{molecules}$$

$$=3\times3.89\times{10}^{-6}\times80\times{10}^{-3}\times6.02\times{10}^{23}$$

$$=5.62\times{10}^{17}$$

The AQE was calculated as

$$\mathrm{AQE}=\frac{N_{\mathrm{re}}}{N_{\mathrm{in}}}\times100\%=\frac{5.62\times{10}^{17}}{3.87\times{10}^{20}}\times100\%=0.145\%$$

**Table** **S6.** SCCE values of the MoO_3–_*_x_* and MoO_3–_*_x_*/10% Ag samples under the optimal conditions.

| Sample | NH_3_ yield  (mmol g_cat_^−1^ h^−1^) | SCCE  (%) |
| --- | --- | --- |
| MoO_3–_*_x_* (isothermal) | 0.22 (± 0.04) | 0.04 (± 0.01) |
| MoO_3–_*_x_*/10% Ag (isothermal)  MoO_3–_*_x_*/10% Ag (non-isothermal) | 0.78 (± 0.08)  1.00 (± 0.14) | 0.14 (± 0.01)  0.18 (± 0.03) |

Take the determination of the SCCE of the MoO_3–_*_x_*/10% Ag sample under non-isothermal conditions as an example. The produced NH_3_ amount after temperature stabilization in 2 h was

$$n_{\mathrm{ammonia}}={\Delta c}_{\mathrm{ammonia}}V_{\mathrm{solution}}=\left( 3.99863-1.9965 \right)\times80\times{10}^{-3}=0.16 \mathrm{mmol}$$

The diameter of the reactor was 7.2 cm. The illumination area of the solution was

$$S=\pi\times(\frac{d}{2})^{2}=3.1415926 \times{(\frac{7.2}{2})}^{2}=40.72 cm^{2}$$

The total input energy under simulated solar light illumination was

$$P=IS=100 mW cm^{-2}\times40.72 cm^{2}=4.07 W$$

The SCCE was calculated as

$$\mathrm{SCCE}=\frac{{\Delta G}_{\mathrm{ammonia}} n_{\mathrm{ammonia}}}{P_{\mathrm{light}} t}\times100\%=\frac{339\times{10}^{3}\times0.16\times{10}^{-3}}{4.07\times7200}\times100\%=0.18\%$$

**Table** **S7.** AQEs and SCCEs reported in representative PCNF works.

| Year | | Catalyst | Light source | | Reaction conditions | NH_3_ yield  (mmol g_cat_^−1^ h^−1^) | AQE (wavelength) | SCCE | Ref. |
| --- | --- | --- | --- | --- | --- | --- | --- | --- | --- |
| 2025 | MoO_3–_*_x_*/10% Ag/PVA film | 300 W xenon lamp, AM 1.5 | 120 mg catalyst, 80 mL H_2_O, 25 °C, air | | 0.30 | 0.178%  (600 nm) | 0.28% | This work |  |
| 2025 | MoO_3–_*_x_*/10% Ag | 300 W xenon lamp, AM 1.5 | 80 mg catalyst, 80 mL H_2_O, non-isothermal, N_2_ | | 1.00 | N/A | 0.18% | This work |  |
| 2025 | MoO_3–_*_x_*/10% Ag | 300 W xenon lamp, AM 1.5 | 80 mg catalyst, 80 mL H_2_O, 25 °C, N_2_ | | 0.78 | 0.145%  (350 nm) | 0.14% | This work |  |
| 2025 | MoO_3–_*_x_* | AM 1.5G lamp, AM 1.5 | 3 g catalyst, 700 mL H_2_O, 25 °C, N_2_ | | 1.260 | 0.95%  (650 nm) | 0.3% | [8] |  |
| 2021 | Fe-S_2_-Mo | 70 W tungsten lamp, *λ* ≥ 400 nm | 1 mg catalyst, 10 mL H_2_O, 270 °C, N_2_ | | 2.1 | 37.1%  (432 nm) | 0.24% | [16] |  |
| 2018 | P-doped g-C_3_N_4_-NVs | 2000 W xenon lamp, *λ* > 420 nm | 200 mg catalyst, 100 mL H_2_O, 30 °C, N_2_ | | N/A | 1%  (420 nm) | 0.1% | [17] |  |
| 2022 | Ti-Bi_2_O_3_ | 300 W xenon lamp, AM 1.5 | 150 mg catalyst, 150 mL H_2_O, 25 °C, N_2_ | | 0.509 | 2.21%  (350 nm) | 0.095% | [18] |  |
| 2022 | MoO_3–_*_x_* | 300 W xenon lamp, AM 1.5 | 200 mg catalyst, 80 mL H_2_O, 25 °C, N_2_ | | 0.123 | 1.24%  (808 nm) | 0.057% | [1] |  |
| 2020 | BiOCl-OVs | 300 W xenon lamp, AM 1.5 | 200 mg catalyst, 100 mL H_2_O, 30 °C, N_2_ | | N/A | N/A | 0.05% | [19] |  |
| 2020 | Ru-V_S_-CoS/CN | 300 W xenon lamp, AM 1.5 | 25 mg catalyst, 50 mL H_2_O, 25 °C, N_2_ | | 0.438 | 1.28%  (400 nm) | 0.042% | [20] |  |
| 2020 | Au/C_3_N_4_-NV | 300 W xenon lamp, *λ* > 420 nm | 50 mg catalyst, 20 mL ethanol, 80 ml H_2_O, 25 °C, N_2_ | | 0.783 | 0.64%  (550 nm) | 0.032% | [21] |  |
| 2018 | Mo-W_18_O_49_ | 300 W xenon lamp, AM 1.5 | 10 mg catalyst, 10 mL H_2_O (1 mM Na_2_SO_3_), 25 °C, N_2_ | | 0.196 | 0.33%  (400 nm) | 0.028% | [22] |  |
| 2021 | 3%-S/Cu | 300 W xenon lamp, AM 1.5 | 10 mg catalyst, 20 mL H_2_O, 25 °C, N_2_ | | 0.157 | 0.042%  (490 nm) | 0.023% | [23] |  |
| 2017 | JRC-TIO-6 (rutile) | 300 W mercury lamp, *λ* > 280 nm | 200 mg catalyst, 200 mL H_2_O, 40 °C, N_2_ | | N/A | 0.7%  (350 nm) | 0.02% | [24] |  |
| 2021 | 2.3% Mn-WO_3_ | 300 W xenon lamp, AM 1.5 | 10 mg catalyst, 20 mL H_2_O, 25 °C, N_2_ | | 0.425 | 0.18%  (450 nm) | 0.019% | [25] |  |
| 2020 | Cu-ZnAl-LDH | 300 W xenon lamp, AM 1.5 | 5 mg catalyst, 100 mL H_2_O, 25 °C, N_2_ | | 0.110 | 1.77%  (265 nm) | 0.014% | [26] |  |
| 2022 | BVO/SV-ZIS | 300 W xenon lamp, *λ* > 400 nm | 50 mg catalyst, 200 mL H_2_O, 25 °C, N_2_ | | 0.081 | N/A | 0.012% | [27] |  |
| 2017 | Au/(BiO)_2_CO_3_ | 300 W xenon lamp, AM 1.5 | 20 mg catalyst, 20 mL H_2_O, 25 °C, N_2_ | | 0.038 | N/A | 0.006% | [28] |  |
| 2018 | g-C_3_N_4_ after KOH etching | 300 W xenon lamp, AM 1.5 | 20 mg catalyst, 150 mL CH_3_OH, 25 °C, N_2_ | | 3.632 | 21.5%  (420 nm) | N/A | [29] |  |
| 2022 | GSCe | 6 W LED lamp | 20 mg catalyst, 20 mL H_2_O, 25 °C, N_2_ | | 0.110 | 9.25 %  (365 nm) | N/A | [30] |  |
| 2021 | GDY@CoO*_x_*QD | 300 W xenon lamp, AM 1.5 | 30 mL 0.1 M Na_2_SO_4_ in H_2_O, 20 °C, N_2_ | | 19.583 | 8.73 %  (500 nm) | N/A | [31] |  |
| 2022 | Ru-SA/H*_x_*MoO_3–_*_y_* | 300 W xenon lamp, *λ* > 420 nm | 15 mg catalyst, 25 °C, N_2_ and H_2_ | | 4.000 | 6%  (650 nm) | N/A | [32] |  |
| 2016 | CdS:MoFe protein | 405 nm diode, 25 mW·cm^−2^ | 300 μL solution of 16.7 nM catalyst, 500 mM HEPES, 25 °C, N_2_ | | 0.019 | 3.30%  (405 nm) | N/A | [33] |  |
| 2017 | Bi_5_O_7_Br-OV | 300 W xenon lamp, *λ* > 400 nm | 25 mg catalyst, 100 mL H_2_O, 25 ºC, N_2_ | | 1.380 | 2.30%  (420 nm) | N/A | [34] |  |
| 2020 | Gd-IHEP-8 | 300 W xenon lamp, AM 1.5 | 20 mg catalyst, 100 mL H_2_O, 25 ºC, N_2_ | | 0.220 | 2.25%  (365 nm) | N/A | [35] |  |
| 2019 | Fe-BiOCl nanosheets | 300 W xenon lamp, AM 1.5 | 10 mg catalyst, 20 mL H_2_O, 25 ºC, N_2_ | | 1.022 | 1.80%  (420 nm) | N/A | [36] |  |
| 2019 | Bi_3_O_4_Br | 300 W xenon lamp, AM 1.5 | 50 mg catalyst, 50 mL H_2_O, 30 °C, N_2_ | | 0.051 | 1.59%  (400 nm) | N/A | [37] |  |
| 2021 | Au@UiO-66/PTFE membrane | 300 W xenon lamp, *λ* > 400 nm | Membrane, 80 mL H_2_O (0.5 M K_2_SO_4_), 25 °C, N_2_ | | 0.360 | 1.54%  (520 nm) | N/A | [38] |  |
| 2019 | Au/P25 | 300 W xenon lamp, AM 1.5 | 5 mg catalyst, ethanol 1 M, 50 mL H_2_O, 20 °C, N_2_ | | 1.020 | 0.93%  (550 nm) | N/A | [39] |  |
| 2019 | Fe^3+^/g-C_3_N_4_ | 300 W xenon lamp, *λ* > 420 nm | 20 mg catalyst, 10 mL ethanol, 40 mL H_2_O, 25 °C, N_2_ | | 0.125 | 0.86%  (420 nm) | N/A | [40] |  |
| 2018 | Au/TiO_2_-OV | 300 W xenon lamp, *λ* > 420 nm | 100 mg catalyst, 8 mL ethanol, 72 mL H_2_O, 25 °C, N_2_ | | 0.131 | 0.82%  (550 nm) | N/A | [41] |  |
| 2019 | Cu-TiO_2_ | 300 W xenon lamp, AM 1.5 | 20 mg catalyst, 20 mL H_2_O, 25 °C C, N_2_ | | 0.079 | 0.74%  (380 nm) | N/A | [42] |  |
| 2016 | Bi_2_MoO_6_ | 300 W xenon lamp, *λ* > 420 nm | 50 mg catalyst, 100 mL H_2_O, 25 °C, air | | 1.3 | 0.73%  (500 nm) | N/A | [43] |  |
| 2013 | Diamond | 450 W high pressure mercury/ xenon lamp, *λ* > 190 nm | 0.1 wt% catalyst, H_2_O, 25 °C, N_2_ | | 0.001 | ~0.6%  (211.5 nm) | N/A | [44] |  |
| 2021 | Fe-TiO_2_/Au | 300 W xenon lamp, *λ* > 420 nm | 100 mg catalyst, 80 mL H_2_O, 25 °C, N_2_ | | 0.050 | 0.39%  (600 nm) | N/A | [45] |  |
| 2019 | SmOCl | 300 W xenon lamp, AM 1.5 | 10 mg catalyst, 20 mL H_2_O, 25 °C, N_2_ | | 0.426 | 0.32%  (420 nm) | N/A | [46] |  |
| 2021 | 5-FTNFs | 300 W xenon lamp, AM 1.5 | 5 mg catalyst, 15 mL H_2_O, 25 °C, N_2_ | | 0.064 | ~0.32%  (380 nm) | N/A | [47] |  |
| 2019 | AuRu_0.31_ | 300 W xenon lamp, AM 1.5 | 0.2 mg catalyst, 3 mL H_2_O, 25 °C, N_2_ | | 0.101 | 0.021%  (350 nm) | N/A | [48] |  |
| 2020 | Bi_5_O_7_Br-40 | 300 W xenon lamp, *λ* > 400 nm | 25 mg catalyst, 100 mL H_2_O, 25 °C, N_2_ | | 12.72 | N/A | N/A | [49] |  |
| 2019 | *m*CNN | 300 W xenon lamp, *λ* > 400 nm | 10 mg catalyst, 40 mL ethylene glycol, 25 °C, N_2_ | | 3.420 | N/A | N/A | [50] |  |
| 2021 | GDY@Fe-B | 300 W xenon lamp, *λ* > 420 nm | 100 mL Na_2_SO_4_ solution, H_2_O, 25 °C, N_2_ | | 1.916 | N/A | N/A | [51] |  |
| 2023 | g-C_3_N_4_-TiO_2_ | 300 W xenon lamp, *λ* > 420 nm | 50 mg catalyst, 50 mL H_2_O, 25 °C, N_2_ | | 1.06 | N/A | N/A | [52] |  |
| 2021 | TiO_2_-Au-BiOI | 300 W xenon lamp, AM 1.5 | 10 mg catalyst, 100 mL H_2_O, 30 °C, N_2_ | | 0.535 | N/A | N/A | [53] |  |
| 2018 | SiW_12_/K-C_3_N_4_ | 300 W xenon lamp, *λ* < 420 nm | 50 mg catalyst, 200 mL H_2_O, 25 °C, N_2_ | | 0.353 | N/A | N/A | [54] |  |
| 2024 | Chiral Au@CeO2 | Linear or circular polarized light (808 nm) | 5.5 mg catalyst, 100 mL H_2_O, 25 mL methanol, 25 °C, N_2_ | | 0.175 | N/A | N/A | [55] |  |
| 2022 | NJUZ-1 | 300 W xenon lamp | 20 mg catalyst, 60 mL H_2_O, 25 °C, N_2_ | | 0.140 | N/A | N/A | [56] |  |

**Table** **S8.** ICP-MS results of the photocatalysts.

| Photocatalyst | Element | Content  (mg g^−1^) | Mass ratio  (Ag/(Ag+Mo)) | Molar ratio  (Ag/(Ag+Mo)) |
| --- | --- | --- | --- | --- |
| MoO_3–_*_x_*/5% Ag | Mo (1st) | 454.71 | 5.24% | 4.69% |
|  | Ag (1st) | 25.14 |  |  |
|  | Mo (2nd) | 464.13 | 5.29% | 4.73% |
|  | Ag (2nd) | 25.91 |  |  |
|  | Mo (3rd) | 448.88 | 5.31% | 4.75% |
|  | Ag (3rd) | 25.19 |  |  |
| MoO_3–_*_x_*/10% Ag  (before reaction) | Mo (1st) | 487.80 | 10.74% | 9.66% |
|  | Ag (1st) | 58.67 |  |  |
|  | Mo (2nd) | 468.83 | 11.00% | 9.90% |
|  | Ag (2nd) | 57.94 |  |  |
|  | Mo (3rd) | 468.25 | 10.91% | 9.82% |
|  | Ag (3rd) | 57.34 |  |  |
| MoO_3–_*_x_*/10% Ag  (after reaction) | Mo (1st) | 474.46 | 10.29% | 9.25% |
|  | Ag (1st) | 54.40 |  |  |
|  | Mo (2nd) | 462.70 | 10.41% | 9.37% |
|  | Ag (2nd) | 53.78 |  |  |
|  | Mo (3rd) | 481.94 | 9.94% | 8.94% |
|  | Ag (3rd) | 53.21 |  |  |

**Table** **S9.** Calculated water absorption of the various systems at different wavelengths.

| Wavelength (nm) | 800 | 900 | 980 | 1064 | 1200 |
| --- | --- | --- | --- | --- | --- |
| Powder system (~2.1 cm) (%) | 3.64 | 23.95 | 64.50 | 29.04 | 93.04 |
| 1 mm thickness film system (%) | 0.18 | 1.30 | 4.81 | 1.62 | 11.92 |
| 0.1 mm thickness film system (%) | 0.02 | 0.13 | 0.49 | 0.16 | 1.26 |
| 2.5 μm thickness film system (%) | 4.4×10^−4^ | 3.3×10^−3^ | 0.01 | 4.1×10^−3^ | 0.03 |
| 250 nm thickness film system (%) | 4.4×10^−5^ | 3.3×10^−4^ | 1.2×10^−3^ | 4.1×10^−4^ | 3.2×10^−3^ |

Take the determination of the water absorption at 980 nm in the powder system (*l*_powder_ = 2.1 cm) and 1 mm thickness film system (*l*_film_ = 1 mm) as an example. The measured light absorbance *A* of pure water in a 1 cm thickness cuvette (*l* = 1 cm) at 980 nm was 0.2142. According to the Beer–Lambert Law and Equation (3),

$$A_{\mathrm{powder}}=\varepsilon l_{\mathrm{powder}}c=A\frac{l_{\mathrm{powder}}}{l}=0.2142\times\frac{2.1}{1}=0.4498$$

$$A_{\mathrm{film}}=\varepsilon l_{\mathrm{film}}c=A\frac{l_{\mathrm{film}}}{l}=0.2142\times\frac{0.1}{1}=0.02142$$

$$\%A_{\mathrm{powder}}=\left( 1-{0.1}^{A_{\mathrm{powder}}} \right)\times100\%=\left( 1-{0.1}^{0.4498} \right)\times100\%=64.50\%$$

$$\%A_{\mathrm{film}}=\left( 1-{0.1}^{A_{\mathrm{film}}} \right)\times100\%=\left( 1-{0.1}^{0.02142} \right)\times100\%=4.81\%$$

**Table** **S10.** Effect of the height of the film above the water surface on the photocatalytic performance.

| Water volume  (mL) | Height of the film above  the water surface (mm) | NH_3_ yield  (mmol g_cat_^−1^ h^−1^) | SCCE  (%) |
| --- | --- | --- | --- |
| 3.7873 | 3 | 0 | 0 |
| 4.5450 | 3 | 0.02 (± 0.002) | 0.01 (± 0.001) |
| 5.3037 | 3 | 0.07 (± 0.005) | 0.04 (± 0.003) |
| 6.0876 | 2 | 0.07 (± 0.004) | 0.05 (± 0.003) |
| 6.6439 | 1.5 | 0.08 (± 0.004) | 0.05 (± 0.002) |
| 7.1972 | 1 | 0.09 (± 0.004) | 0.06 (± 0.003) |
| 7.7501 | 0.5 | 0.07 (± 0.002) | 0.05 (± 0.001) |
| 8.3063 | 0 | 0.04 (± 0.005) | 0.03 (± 0.003) |
| 9.4115 | 0 | 0.04 (± 0.004) | 0.02 (± 0.003) |
| 10.5201 | 0 | 0.03 (± 0.004) | 0.02 (± 0.003) |
| 11.6272 | 0 | 0.03 (± 0.004) | 0.02 (± 0.002) |
| 12.7378 | 0 | 0.03 (± 0.004) | 0.02 (± 0.002) |

**Table** **S11.** Effect of the photocatalyst loading amount on the photocatalytic performance.

| Photocatalyst loading  amount (mg) | NH_3_ yield  (mmol g_cat_^−1^ h^−1^) | SCCE  (%) |
| --- | --- | --- |
| 10 | 0.25 (± 0.02) | 0.02 (± 0.002) |
| 20 | 0.28 (± 0.03) | 0.04 (± 0.004) |
| 40 | 0.30 (± 0.03) | 0.10 (± 0.01) |
| 60 | 0.31 (± 0.02) | 0.14 (± 0.01) |
| 80 | 0.31 (± 0.008) | 0.22 (± 0.006) |
| 100 | 0.30 (± 0.02) | 0.25 (± 0.01) |
| 120 | 0.30 (± 0.01) | 0.28 (± 0.01) |
| 140 | 0.27 (± 0.02) | 0.34 (± 0.02) |
| 160 | 0.28 (± 0.02) | 0.35 (± 0.02) |
| 200 | 0.25 (± 0.01) | 0.39 (± 0.02) |
| 240 | 0.23 (± 0.02) | 0.41 (± 0.03) |

A thin water layer not only significantly reduces the light attenuation by water but also accelerates the reaction kinetics of nitrogen mass transfer. Air can be directly used as the feeding gas. Here we would like to verify that N_2_ in air is sufficient to support the PCNF reaction.

As the reaction was conducted in aqueous solutions, H_2_O should be in excess. We assume that nitrogen molecules in air are insufficient to support the occurrence of the reaction. The consumed N_2_ by the photocatalytic N_2_ fixation reaction cannot be efficiently provided by the diffusion process from air. The reaction is limited by the diffusion.

The NH_3_ yield (120 mg photocatalyst) was

$$y_{NH_{3}}=0.298 \mathrm{mmol} g_{\mathrm{cat}}^{-1} h^{-1}$$

For 120 mg photocatalyst, the N_2_ consumption rate was

$$r_{N_{2}}={\frac{1}{2}y}_{NH_{3}}m_{\mathrm{cat}}=\frac{1}{2}\times0.298\times0.12=0.018 \mathrm{mmol} h^{-1}=0.005 \mu mol s^{-1}$$

The transition from convection to diffusion is gradual upon approaching the photocatalyst surface, but it is convenient to model this situation as two distinct regions: a convectively supplied reservoir of N_2_, and a diffusive boundary layer between this reservoir and the photocatalyst surface. We assume that the diffusive boundary layer is located in the adsorbed water layer. The reactant concentration *n*_A_ just over the surface is always lower than that in the reservoir *n*_∞_. This concentration gradient drives the reactant diffusion flux *J*_A_. The edge of the concentration boundary layer in which this diffusion occurs, *δ*_n_, is arbitrarily but usually defined as the plane at which the reactant concentration drops by 1% from *n*_∞_

*n*_∞_ = 3.5 × 10^−2^ mol L^−1^, 99%*n*_∞_ = 3.45 × 10^−2^ mol L^−1^

The diffusion flux toward the surface of an individual photocatalyst nanosphere *J*_A_ can be calculated from the following formula

$J_{A}=-D\frac{{99\%n}_{\infty}-n_{A}}{\delta_{n}}$ (22)

where *D* is the diffusion coefficient at the atmospheric pressure and a given temperature. In our work, the value of *D* for N_2_ in water at 25 ℃ and the atmospheric pressure was 2.00×10^−5^ cm^2^ s^−1^.

As the reaction is assumed to be diffusion-controlled, all diffused N_2_ molecules would be consumed at the surface of the photocatalyst nanosphere (*n*_A_ = 0). The reaction rate of an individual nanosphere can be calculated to be

$r=-J_{A}\times4\pi R^{2}$ (23)

where *R* is the radius of the nanosphere. The reaction rate of the entire solution is

$r_{\mathrm{total}}=r\times the number of the nanospheres$ (24)

In our work, the average size of the nanospheres was ~550 nm. We assume that these nanospheres are dispersed evenly in the reaction solution. The density *ρ* of MoO_3_ (4.69 g cm^−3^) can represent that of the MoO_3–_*_x_*/10% Ag nanospheres.

For 120 mg MoO_3–_*_x_*/10% Ag nanospheres, the number of the nanospheres = $\frac{V_{\mathrm{total}}}{V_{\mathrm{individual}}}=\frac{\frac{m}{\rho}}{\frac{4\pi R^{3}}{3}}$ = $\frac{\frac{{120\times10}^{-3}}{4.69}}{\frac{4\pi\times\left( 550/2\times{10}^{-7} \right)^{3}}{3}}$ = 2.94 × 10^11^, where *V*_total_ is the total volume of the nanospheres, *V*_individual_ is the volume of an individual nanosphere, and *m* is the mass of the nanosphere sample added in the reaction solution.

The thickness of the boundary layer (*δ*_n_) can be calculated from Equations (22), (23), and (24) to be *δ*_n_ = 385.56 cm. If *δ*_n_ was larger than 385.56 cm, the reaction would be diffusion-controlled and air would be insufficient to support the photocatalytic reaction. However, the measured *δ*_n_ is only ~250 nm (<<385.56 cm), which means that air is well qualified to be employed as the feeding gas.

**Table** **S12.** Measured AQEs of the MoO_3–_*_x_*/10% Ag powder and MoO_3–_*_x_*/10% Ag/PVA solar absorber film under the optimal conditions.

|  | MoO_3–_*_x_*/10% Ag powder | | | | MoO_3–_*_x_*/10% Ag/PVA solar absorber film | | | |
| --- | --- | --- | --- | --- | --- | --- | --- | --- |
| Wavelength (nm) | NH_3_ produced  (μmol L^−1^ h^−1^) | Light power density  (mW cm^−2^) | Light power  *P* (mW) | AQE  (%) | NH_3_ produced  (μmol L^−1^ h^−1^) | Light power density  (mW cm^−2^) | Light power  *P* (mW) | AQE  (%) |
| 350 | 3.89 | 1.5 | 61.08 | 0.145 | 0.74 | 1.1 | 9.99 | 0.169 |
| 380 | 10.73 | 4.3 | 175.10 | 0.129 | 2.95 | 4.2 | 38.13 | 0.163 |
| 420 | 27.57 | 10.2 | 415.34 | 0.126 | 7.97 | 10.5 | 95.33 | 0.159 |
| 475 | 36.17 | 12.4 | 504.93 | 0.121 | 10.69 | 12.4 | 112.58 | 0.159 |
| 520 | 45.31 | 13.7 | 557.86 | 0.125 | 13.03 | 13.5 | 122.57 | 0.163 |
| 550 | 43.52 | 12.4 | 504.93 | 0.125 | 12.92 | 12.5 | 113.49 | 0.165 |
| 600 | 47.41 | 12.2 | 496.78 | 0.127 | 14.51 | 12.0 | 108.95 | 0.178 |
| 650 | 57.06 | 13.3 | 541.58 | 0.129 | 17.20 | 13.3 | 120.75 | 0.175 |
| 700 | 58.97 | 13.3 | 541.58 | 0.124 | 18.31 | 13.3 | 120.75 | 0.173 |
| 808 | 7.73 | / | 63.14 | 0.121 | 12.33 | / | 71.13 | 0.171 |
| 980 | 2.81 | / | 52.75 | 0.118 | 15.12 | / | 74.69 | 0.172 |

**References**

[1] Bai, H. Y., Lam, S. H., Yang, J. H., Cheng, X. Z., Li, S. S., Jiang, R. B., Shao, L., & Wang, J. F., A Schottky-barrier-free Plasmonic Semiconductor Photocatalyst for Nitrogen Fixation in a “One-stone-two-birds” Manner, 2022, Adv. Mater., 34, 2104226, <https://advanced.onlinelibrary.wiley.com/doi/10.1002/adma.202104226>

[2] Bai, H. Y., Hu, J. T., Lam, S. H., Guo, Y. Z., Zhu, X.-M., Yang, Z., & Wang, J. F., Turning Dielectric MoO_3_ Nanospheres from White to Black through Doping for Efficient Solar Seawater Desalination, 2022, ACS Mater. Lett., 4, 1584, <https://pubs.acs.org/doi/10.1021/acsmaterialslett.2c00402>

[3] Huang, H. N., Shi, R., Li, Z. H., Zhao, J. Q., Su, C. L., & Zhang, T. R., Triphase Photocatalytic CO_2_ Reduction over Silver-decorated Titanium Oxide at a Gas–Water Boundary, 2022, Angew. Chem. Int. Ed., 61, e202200802, <https://onlinelibrary.wiley.com/doi/10.1002/anie.202200802>

[4] Spagnol, V., Sutter, E., Debiemme-Chouvy, C., Cachet, H., & Baroux, B., EIS Study of Photo-induced Modifications of Nano-columnar TiO_2_ Films, 2009, Electrochim. Acta, 54, 1228, <https://www.sciencedirect.com/science/article/pii/S0013468608010839>

[5] Lajaunie, L., Boucher, F., Dessapt, R., & Moreau, P., Strong Anisotropic Influence of Local-field Effects on the Dielectric Response of α-MoO_3_, 2013, Phys. Rev. B, 88, 115141, <https://journals.aps.org/prb/abstract/10.1103/PhysRevB.88.115141>

[6] Zhao, Y. X., Wu, F., Miao, Y. X., Zhou, C., Xu, N., Shi, R., Wu, L.-Z., Tang, J. W., & Zhang, T. R., Revealing Ammonia Quantification Minefield in Photo/Electrocatalysis, 2021, Angew. Chem. Int. Ed., 60, 21728, <https://onlinelibrary.wiley.com/doi/full/10.1002/anie.202108769>

[7] Safavi, A., & Ensafi, A. A., Kinetic Spectrophotometric Determination of Hydrazine, 1995, Anal. Chim. Acta, 300, 307, <https://www.sciencedirect.com/science/article/pii/000326709400383W>

[8] Wang, J. H., Ran, G. L., Gao, J. Y., Li, D., Waterhouse, G. I. N., Shi, R., Zhang, W. K., Tang, J. W., Wu, L.-Z., Zhao, Y. X., & Zhang, T. R., Solar-driven Conversion of Nitrogen and Water to Solid Fertilizer in an Outdoor 1 m^2^ Panel Reactor, 2025, Adv. Mater., 37, 2420199, <https://advanced.onlinelibrary.wiley.com/doi/10.1002/adma.202420199>

[9] Peter, L. M., Wijayantha, K. G. U., & Tahir, A. A., Kinetics of Light-driven Oxygen Evolution at α-Fe_2_O_3_ Electrodes, 2012, Faraday Discuss., 155, 309, <https://pubs.rsc.org/en/content/articlelanding/2012/fd/c1fd00079a>

[10] Zheng, J.-Y., Bao, S.-H., Guo, Y., & Jin, P., Natural Hydrophobicity and Reversible Wettability Conversion of Flat Anatase TiO_2_ Thin Film, 2014, ACS Appl. Mater. Interfaces, 6, 1351, <https://pubs.acs.org/doi/10.1021/am404470e>

[11] Azimi, G., Dhiman, R., Kwon, H.-M., Paxson, A. T., & Varanasi, K. K., Hydrophobicity of Rare-rarth Oxide Ceramics, 2013, Nat. Mater., 12, 315, <https://www.nature.com/articles/nmat3545>

[12] Mallamace, F., Broccio, M., Corsaro, C., Faraone, A., Majolino, D., Venuti, V., Liu, L., Mou, C.-Y., & Chen, S.-H., Evidence of the Existence of the Low-density Liquid Phase in Supercooled, Confined Water, 2007, Proc. Natl. Acad. Sci. U. S. A., 104, 424, <https://www.pnas.org/doi/10.1073/pnas.0607138104>

[13] Wagner, R., Benz, S., Möhler, O., Saathoff, H., Schnaiter, M., & Schurath, U., Mid-infrared Extinction Spectra and Optical Constants of Supercooled Water Droplets, 2005, J. Phys. Chem. A, 109, 7099, <https://pubs.acs.org/doi/10.1021/jp051942z>

[14] Dey, A., Mondal, S. I., Sen, S., Ghosh, D., & Patwari, G. N., Electrostatics Determine Vibrational Frequency Shifts in Hydrogen Bonded Complexes, 2014, Phys. Chem. Chem. Phys., 16, 25247, <https://pubs.rsc.org/en/content/articlelanding/2014/cp/c4cp04617j>

[15] Molybdic acid, [https://zh.wikipedia.org/zh-hans/%E9%92%BC%E9%85%B8 (2010)](https://zh.wikipedia.org/zh-hans/%E9%92%BC%E9%85%B8%20(2010))

[16] Zheng, J. W., Lu, L. L., Lebedev, K., Wu, S., Zhao, P., McPherson, I. J., Wu, T.-S., Kato, R., Li, Y. Y., Ho, P.-L., Li, G. C., Bai, L. L., Sun, J. H., Prabhakaran, D., Taylor, R. A., Soo, Y.-L., Suenaga, K., & Tsang, S. C. E., Fe on Molecular-Layer MoS_2_ as Inorganic Fe-S_2_-Mo Motifs for Light-Driven Nitrogen Fixation to Ammonia at Elevated Temperatures, 2021, Chem Catal., 1, 162, <https://www.sciencedirect.com/science/article/pii/S2667109321000099>

[17] Shiraishi, Y., Shiota, S., Kofuji, Y., Hashimoto, M., Chishiro, K., Hirakawa, H., Tanaka, S., Ichikawa, S., & Hirai, T., Nitrogen Fixation with Water on Carbon-nitride-based Metal-free Photocatalysts with 0.1% Solar-to-Ammonia Energy Conversion Efficiency, 2018, ACS Appl. Energy Mater., 1, 4169, <https://pubs.acs.org/doi/10.1021/acsaem.8b00829>

[18] Wu, P. F., Wang, T. Y., Xue, Q., Wang, M. K., Zhong, R. H., Hu, J., Chen, Z., Wang, D. J., & Xue, G. L., Regulating Electronic Structure in Bi_2_O_3_ Architectures by Ti Mediation: A Strategy for Dual Active Sites Synergistically Promoting Photocatalytic Nitrogen Hydrogenation, 2022, ChemSusChem, 15, e202200297, <https://chemistry-europe.onlinelibrary.wiley.com/doi/10.1002/cssc.202200297>

[19] Shiraishi, Y., Hashimoto, M., Chishiro, K., Moriyama, K., Tanaka, S., & Hirai, T., Photocatalytic Dinitrogen Fixation with Water on Bismuth Oxychloride in Chloride Solutions for Solar-to-Chemical Energy Conversion, 2020, J. Am. Chem. Soc., 142, 7574, <https://pubs.acs.org/doi/10.1021/jacs.0c01683>

[20] Yuan, J. L., Yi, X. Y., Tang, Y. H., Liu, M. J., & Liu C. B., Efficient Photocatalytic Nitrogen Fixation: Enhanced Polarization, Activation, and Cleavage by Asymmetrical Electron Donation to N≡N Bond, 2020, Adv. Funct. Mater., 30, 1906983, <https://advanced.onlinelibrary.wiley.com/doi/full/10.1002/adfm.201906983>

[21] Guo, Y. Z., Yang, J. H., Wu, D. H., Bai, H. Y., Yang, Z., Wang, J. F., & Yang, B. C., Au Nanoparticle-embedded, Nitrogen-deficient Hollow Mesoporous Carbon Nitride Spheres for Nitrogen Photofixation, 2020, J. Mater. Chem. A, 8, 16218, <https://pubs.rsc.org/en/content/articlelanding/2020/ta/d0ta03793a>

[22] Zhang, N., Jalil, A., Wu, D. X., Chen, S. M., Liu, Y. F., Gao, C., Ye, W., Qi, Z. M., Ju, H. X., Wang, C. M., Wu, X. J., Song, L., Zhu, J. F., & Xiong Y. J., Refining Defect States in W_18_O_49_ by Mo Doping: A Strategy for Tuning N_2_ Activation towards Solar-driven Nitrogen Fixation, 2018, J. Am. Chem. Soc., 140, 9434, <https://pubs.acs.org/doi/10.1021/jacs.8b02076>

[23] Xin, Y., Wang, S. M., Yuan, H. B., Hou, T. T., Zhu, W. K., Liu, Y. X., Yao, Y., Zhang, W. H., Liang, S. Q., & Wang, L. B., Atomic-Level Insights into the Activation of Nitrogen *via* Hydrogen-Bond Interaction toward Nitrogen Photofixation, 2021, Chem, 7, 2118, <https://www.sciencedirect.com/science/article/pii/S2451929421001686>

[24] Hirakawa, H., Hashimoto, M., Shiraishi, Y., & Hirai, T., Photocatalytic Conversion of Nitrogen to Ammonia with Water on Surface Oxygen Vacancies of Titanium Dioxide, 2017, J. Am. Chem. Soc., 139, 10929, <https://pubs.acs.org/doi/10.1021/jacs.7b06634>

[25] Zhang, Y. D., Hou, T. T., Xu, Q., Wang, Q. Y., Bai, Y., Yang, S. K., Rao, D. W., Wu, L. H., Pan, H. B., Chen, J. F., Wang, G. M., Zhu, J. F., Yao, T., & Zheng, X. S., Dual-metal Sites Boosting Polarization of Nitrogen Molecules for Efficient Nitrogen Photofixation, 2021, Adv. Sci., 8, 2100302, <https://advanced.onlinelibrary.wiley.com/doi/10.1002/advs.202100302>

[26] Zhang, S., Zhao, Y. X., Shi, R., Zhou, C., Waterhouse, G. I. N., Wu, L.-Z., Tung, C.-H., & Zhang, T. R., Efficient Photocatalytic Nitrogen Fixation over Cu*^δ^*^+^-Modified Defective ZnAl-Layered Double Hydroxide Nanosheets, 2020, Adv. Energy Mater., 10, 1901973, <https://advanced.onlinelibrary.wiley.com/doi/10.1002/aenm.201901973>

[27] Zhang, G. H., Yuan, X. X., Xie, B., Meng, Y., Ni, Z. M., & Xia, S. J., S Vacancies Act as a Bridge to Promote Electron Injection from Z-Scheme Heterojunction to Nitrogen Molecule for Photocatalytic Ammonia Synthesis, 2022, Chem. Eng. J., 433, 133670, <https://www.sciencedirect.com/science/article/pii/S138589472105244X>

[28] Xiao, C. L., Hu, H., Zhang X. Y., & MacFarlane, D. R., Nanostructured Gold/Bismutite Hybrid Heterocatalysts for Plasmon-enhanced Photosynthesis of Ammonia, 2017, ACS Sustainable Chem. Eng., 5, 10858, <https://pubs.acs.org/doi/full/10.1021/acssuschemeng.7b02788?src=recsys>

[29] Li, X. M., Sun, X., Zhang, L., Sun, S. M., & Wang, W. Z., Efficient Photocatalytic Fixation of N_2_ by KOH-treated g-C_3_N_4_, 2018, J. Mater. Chem. A, 6, 3005, <https://pubs.rsc.org/en/content/articlelanding/2018/ta/c7ta09762j>

[30] Liu, S. X., Teng, Z. Y., Liu, H., Wang, T. Y., Wang, G. X., Xu, Q., Zhang, X. Y., Jiang, M., Wang, C., Huang, W., & Pang, H., A Ce-UiO-66 Metal–Organic Framework-based Graphene-embedded Photocatalyst with Controllable Activation for Solar Ammonia Fertilizer Production, 2022, Angew. Chem. Int. Ed., 61, e202207026, <https://onlinelibrary.wiley.com/doi/10.1002/anie.202207026>

[31] Liu, Y. X., Xue, Y. R., Hui, L., Yu, H. D., Fang, Y., He, F., & Li, Y. L., Porous Graphdiyne Loading CoO_x_ Quantum Dots for Fixation Nitrogen Reaction, 2021, Nano Energy, 89, 106333, <https://www.sciencedirect.com/science/article/pii/S2211285521005887>

[32] Yin, H. B., Chen, Z., Peng, Y., Xiong, S. C., Li, Y. D., Yamashita, H., & Li, J. H., Dual Active Centers Bridged by Oxygen Vacancies of Ruthenium Single-Atom Hybrids Supported on Molybdenum Oxide for Photocatalytic Ammonia Synthesis, 2022, Angew. Chem. Int. Ed., 61, e202114242, <https://onlinelibrary.wiley.com/doi/full/10.1002/anie.202114242>

[33] Brown, K. A., Harris, D. F., Wilker, M. B., Rasmussen, A., Khadka, N., Hamby, H., Keable, S., Dukovic, G., Peters, J. W., Seefeldt, L. C., & King, P. W., Light-driven Dinitrogen Reduction Catalyzed by a CdS:Nitrogenase MoFe Protein Biohybrid, 2016, Science, 352, 448, <https://www.science.org/doi/10.1126/science.aaf2091>

[34] Wang, S. Y., Hai, X., Ding, X., Chang, K., Xiang, Y. G., Meng, X. G., Yang, Z. X., Chen, H., & Ye, J. H., Light-switchable Oxygen Vacancies in Ultrafine Bi_5_O_7_Br Nanotubes for Boosting Solar-driven Nitrogen Fixation in Pure Water, 2017, Adv. Mater., 29, 1701774, <https://advanced.onlinelibrary.wiley.com/doi/full/10.1002/adma.201701774>

[35] Hu, K.-Q., Qiu, P.-X., Zeng, L.-W., Hu, S.-X., Mei, L., An, S.-W., Huang, Z.-W., Kong, X.-H., Lan, J.-H., Yu, J.-P., Zhang, Z.-H., Xu, Z.-F., Gibson, J. K., Chai, Z.-F., Bu, Y.-F., & Shi, W.-Q., Solar-driven Nitrogen Fixation Catalyzed by Stable Radical-containing MOFs: Improved Efficiency Induced by a Structural Transformation, 2020, Angew. Chem. Int. Ed., 59, 20666, <https://onlinelibrary.wiley.com/doi/10.1002/anie.202009630>

[36] Zhang, N., Li, L. G., Shao, Q., Zhu, T., Huang, X. Q., & Xiao, X. H., Fe-doped BiOCl Nanosheets with Light-Switchable Oxygen Vacancies for Photocatalytic Nitrogen Fixation, 2019, ACS Appl. Energy Mater. 2, 8394, <https://pubs.acs.org/doi/10.1021/acsaem.9b01961>

[37] Di, J., Xia, J. X., Chisholm, M. F., Zhong, J., Chen, C., Cao, X. Z., Dong, F., Chi, Z., Chen, H. L., Weng, Y.-X., Xiong, J., Yang, S.-Z., Li, H. M., Liu, Z., & Dai, S., Defect-tailoring Mediated Electron–Hole Separation in Single-unit-cell Bi_3_O_4_Br Nanosheets for Boosting Photocatalytic Hydrogen Evolution and Nitrogen Fixation, 2019, Adv. Mater., 31, 1807576, <https://advanced.onlinelibrary.wiley.com/doi/full/10.1002/adma.201807576>

[38] Chen, L.-W., Hao, Y.-C., Guo, Y., Zhang, Q. H., Li, J. N., Gao, W.-Y., Ren, L. T., Su, X., Hu, L. Y., Zhang, N., Li, S. W., Feng, X., Gu, L., Zhang, Y.-W., Yin, A.-X., & Wang, B., Metal-Organic Framework Membranes Encapsulating Gold Nanoparticles for Direct Plasmonic Photocatalytic Nitrogen Fixation, 2021, J. Am. Chem. Soc., 143, 5727, <https://pubs.acs.org/doi/10.1021/jacs.0c13342>

[39] Bu, T.-A., Hao, Y.-C., Gao, W.-Y., Su, X., Chen, L.-W., Zhang, N., & Yin A.-X., Promoting Photocatalytic Nitrogen Fixation with Alkali Metal Cations and Plasmonic Nanocrystals, 2019, Nanoscale, 11, 10072, [https://pubs.rsc.org/en/content/articlelanding/2019/nr/c9nr02502b#:~:text=Here%2C%20we%20report%20that%20alkali%20metal%20cations%20%28Li,and%20Ag%29%20can%20sensitize%20photocatalysts%20under%20visible%20light.](https://pubs.rsc.org/en/content/articlelanding/2019/nr/c9nr02502b%23:~:text=Here%2C%20we%20report%20that%20alkali%20metal%20cations%20%28Li,and%20Ag%29%20can%20sensitize%20photocatalysts%20under%20visible%20light.)

[40] Yao C. K., Wang, R., Wang, Z. S., Lei, H., Dong, X. P., & He, C. Z., Highly Dispersive and Stable Fe^3+^ Active Sites on 2D Graphitic Carbon Nitride Nanosheets for Efficient Visible-light Photocatalytic Nitrogen Fixation, 2019, J. Mater. Chem. A, 7, 27547, <https://pubs.rsc.org/en/content/articlelanding/2019/ta/c9ta09201c>

[41] Yang, J. H., Guo, Y. Z., Jiang, R. B., Qin, F., Zhang, H., Lu W. Z., Wang, J. F., & Yu, J. C., High-efficiency “Working-in-tandem” Nitrogen Photofixation Achieved by Assembling Plasmonic Gold Nanocrystals on Ultrathin Titania Nanosheets, 2018, J. Am. Chem. Soc., 140, 8497, <https://pubs.acs.org/doi/10.1021/jacs.8b03537>

[42] Zhao Y. X., Zhao, Y. F., Shi, R., Wang, B., Waterhouse, G. I. N., Wu, L.-Z., Tung, C.-H., & Zhang, T. R., Tuning Oxygen Vacancies in Ultrathin TiO_2_ Nanosheets to Boost Photocatalytic Nitrogen Fixation up to 700 nm, 2019, Adv. Mater., 31, 1806482, <https://advanced.onlinelibrary.wiley.com/doi/10.1002/adma.201806482>

[43] Hao, Y. C., Dong, X. L., Zhai, S., Ma, H. C., Wang, X. Y., & Zhang, X. F., Hydrogenated Bismuth Molybdate Nanoframe for Efficient Sunlight-driven Nitrogen Fixation from Air, 2016, Chem. Eur. J., 22, 18722, <https://pubmed.ncbi.nlm.nih.gov/27865005/>

[44] Zhu, D., Zhang, L. H., Ruther, R. E., & Hamers, R. J., Photo-illuminated Diamond as a Solid-state Source of Solvated Electrons in Water for Nitrogen Reduction, 2013, Nat. Mater., 12, 836, <https://www.nature.com/articles/nmat3696>

[45] Yang, J. H., Bai, H. Y., Guo, Y. Z., Zhang, H., Jiang, R. B., Yang, B. C., Wang, J. F., & Yu, J. C., Photodriven Disproportionation of Nitrogen and Its Change to Reductive Nitrogen Photofixation, 2021, Angew. Chem. Int. Ed., 60, 927, <https://onlinelibrary.wiley.com/doi/10.1002/anie.202010192>

[46] Hou, T. T., Guo, R. H., Chen, L. L., Xie, Y. C. Z., Guo, J. S., Zhang, W. H., Zheng, X. S., Zhu, W. K., Tan, X. P., & Wang, L. B., Atomic-level Insights in Tuning Defective Structures for Nitrogen Photofixation over Amorphous SmOCl Nanosheets, 2019, Nano Energy, 65, 104003, <https://www.sciencedirect.com/science/article/pii/S2211285519307104>

[47] Bo, Y. N., Wang, H. Y., Lin, Y. X., Yang, T., Ye, R., Li, Y., Hu, C. Y., Du, P. Y., Hu, Y. G., Liu, Z., Long, R., Gao, C., Ye, B. J., Song, L., Wu, X. J., & Xiong, Y. J., Altering Hydrogenation Pathways in Photocatalytic Nitrogen Fixation by Tuning Local Electronic Structure of Oxygen Vacancy with Dopant, 2021, Angew. Chem. Int. Ed., 60, 16085, <https://onlinelibrary.wiley.com/doi/10.1002/anie.202104001>

[48] Hu C. Y., Chen, X., Jin, J. B., Han,Y., Chen, S. M., Ju, H. X., Cai, J., Qiu, Y. R., Gao, C., Wang, C. M., Qi, Z. M., Long, R., Song, L., Liu, Z., & Xiong, Y. J., Surface Plasmon Enabling Nitrogen Fixation in Pure Water through a Dissociative Mechanism under Mild Conditions, 2019, J. Am. Chem. Soc., 141, 7807, <https://pubs.acs.org/doi/10.1021/jacs.9b01375>

[49] Li, P. S., Zhou, Z. A., Wang, Q., Guo, M., Chen, S. W., Low, J. X., Long, R., Liu, W., Ding, P. R., Wu, Y. Y., & Xiong, Y. J., Visible-light-driven Nitrogen Fixation Catalyzed by Bi_5_O_7_Br Nanostructures: Enhanced Performance by Oxygen Vacancies, 2020, J. Am. Chem. Soc., 142, 12430, <https://pubs.acs.org/doi/10.1021/jacs.0c05097>

[50] Wang W. K., Zhang, H. M., Zhang, S. B., Liu, Y. Y., Wang, G. Z., Sun, C. H., & Zhao, H. J., Potassium-ion-assisted Regeneration of Active Cyano Groups in Carbon Nitride Nanoribbons: Visible-light-driven Photocatalytic Nitrogen Reduction, 2019, Angew. Chem. Int. Ed., 58, 16644, <https://onlinelibrary.wiley.com/doi/10.1002/anie.201908640>

[51] Fang, Y., Xue, Y. R., Hui, L., Yu, H. D., & Li, Y. L., Graphdiyne@Janus Magnetite for Photocatalytic Nitrogen Fixation, 2021, Angew. Chem. Int. Ed., 60, 3170, <https://onlinelibrary.wiley.com/doi/10.1002/anie.202012357>

[52] Kwon, N. H., Park, J., Jin, X. Y., Kim, S.-J., Kim, H., & Hwang, S.-J., Defect-regulated Two-dimensional Superlattice of Holey g‑C_3_N_4_–TiO_2_ Nanohybrids: Contrasting Influence of Vacancy Content on Hybridization Impact and Photocatalyst Performance, 2023, ACS Nano, 17, 23732, <https://pubs.acs.org/doi/10.1021/acsnano.3c07566>

[53] Yu, X. J., Qiu, H. R., Wang, Z., Wang, B., Meng, Q. N., Sun, S. D., Tang, Y. F., & Zhao, K., Constructing the Z-scheme TiO_2_/Au/BiOI Nanocomposite for Enhanced Photocatalytic Nitrogen Fixation, 2021, Appl. Surf. Sci., 556, 149785, <https://www.sciencedirect.com/science/article/pii/S0169433221008618>

[54] Xiao, C. L., Zhang, L., Wang, K. F., Wang, H. P., Zhou, Y. Y., & Wang, W. Z., A New Approach to Enhance Photocatalytic Nitrogen Fixation Performance *via* Phosphate-bridge: A Case Study of SiW_12_/K-C_3_N_4_, 2018, Appl. Catal. B, 239, 260, <https://www.sciencedirect.com/science/article/pii/S0926337318307343>

[55] Wang, F., Yang, W. M., Ding, Q., Xing, X. H., Xu, L. G., Lin, H. W., Xu, C. L., & Li, S., Chiral Au@CeO_2_ Helical Nanorods with Spatially Separated Structures for Polarization-Dependent N_2_ Photofixation, 2025, Angew. Chem. Int. Ed., 64, e202415031, <https://onlinelibrary.wiley.com/doi/full/10.1002/anie.202415031>

[56] Xiong, Y., Li, B., Gu, Y. M., Yan, T., Ni, Z. G., Li, S. H., Zuo, J.-L., Ma, J., & Jin, Z., Photocatalytic Nitrogen Fixation under an Ambient Atmosphere Using a Porous Coordination Polymer with Bridging Dinitrogen Anions, 2023, Nat. Chem., 15, 286, <https://doi.org/10.1038/s41557-022-01088-8>
